# Chickpea shows genotype-specific nodulation responses across soil nitrogen environment and root disease resistance categories

**DOI:** 10.1186/s12870-021-03102-6

**Published:** 2021-07-01

**Authors:** Krista L. Plett, Sean L. Bithell, Adrian Dando, Jonathan M. Plett

**Affiliations:** 1 Hawkesbury Institute for the Environment, Western Sydney University, Penrith, NSW Australia; 2 New South Wales Department of Primary Industries, Tamworth, NSW Australia; 3 New South Wales Department of Primary Industries, Elizabeth Macarthur Agricultural Institute, Menangle, NSW Australia

**Keywords:** Genotype specificity, Soil N environment, Rhizobia, Nodulation regulation, Nodule nitrogen production, Transcriptomic analysis, Root disease resistance

## Abstract

**Background:**

The ability of chickpea to obtain sufficient nitrogen via its symbiotic relationship with *Mesorhizobium ciceri* is of critical importance in supporting growth and grain production. A number of factors can affect this symbiotic relationship including abiotic conditions, plant genotype, and disruptions to host signalling/perception networks. In order to support improved nodule formation in chickpea, we investigated how plant genotype and soil nutrient availability affect chickpea nodule formation and nitrogen fixation. Further, using transcriptomic profiling, we sought to identify gene expression patterns that characterize highly nodulated genotypes.

**Results:**

A study involving six chickpea varieties demonstrated large genotype by soil nitrogen interaction effects on nodulation and further identified agronomic traits of genotypes (such as shoot weight) associated with high nodulation. We broadened our scope to consider 29 varieties and breeding lines to examine the relationship between soilborne disease resistance and the number of nodules developed and real-time nitrogen fixation. Results of this larger study supported the earlier genotype specific findings, however, disease resistance did not explain differences in nodulation across genotypes. Transcriptional profiling of six chickpea genotypes indicates that genes associated with signalling, N transport and cellular localization, as opposed to genes associated with the classical nodulation pathway, are more likely to predict whether a given genotype will exhibit high levels of nodule formation.

**Conclusions:**

This research identified a number of key abiotic and genetic factors affecting chickpea nodule development and nitrogen fixation. These findings indicate that an improved understanding of genotype-specific factors affecting chickpea nodule induction and function are key research areas necessary to improving the benefits of rhizobial symbiosis in chickpea.

**Supplementary Information:**

The online version contains supplementary material available at 10.1186/s12870-021-03102-6.

## Background

The majority of legume and pulse crops are able to supplement their nitrogen (N) nutrition by forming a symbiotic association with N-fixing rhizobial bacteria. Farmers utilize this symbiotic relationship with rhizobia to counter nutrient limitations in soils as these bacteria can provide up to 97% of a plants total N requirements [1]. Further, because these symbioses are responsible for returning significant N into agricultural soils globally [2], they reduce the need for costly fertiliser applications. Unlike other diazotrophic bacteria that fix N while living freely in the soil, rhizobia must first colonise plant roots, forming specialised root organs called nodules. The formation of these nodules is controlled both by a series of abiotic cues and a number of important signalling events between the bacteria and the plant host. Chickpea (*Cicer arietinum*) is a legume of major economic value for food production, with chickpeas consistently being the second or third largest pulse crop grown around the world with nearly 11 million hectares in production [3]. Despite the formation of a functional symbiotic relationship between chickpea and the rhizobial species *Mesorhizobium ciceri*, N-fixation in chickpeas is limited as compared to other pulses. Field studies show that the chickpea-*M. ciceri* symbiosis often only fixes sufficient N to support plant growth with little or no enhancement of soil N concentrations after harvest [4, 5]. In addition, competition between inoculated and endemic but less efficient *Mesorhizobium* strains, as well as soil environment effects both affect successful symbiosis with chickpea [6]. Therefore, a better understanding of the factors that could improve the benefit of this symbiotic association would be advantageous in agriculture.

Abiotic factors such as soil moisture, pH and plant nutrient availability in soils have been identified as affecting nodule formation and N-fixation. With regards to soil nutrients, chickpea nodulation and N-fixation responses are highly dependent on plant available N concentrations in soils. For example, the chickpea var. Tyson, which receives between 60–80% of its N requirements from symbiotic fixation at soil nitrate concentrations between 20–90 kg nitrate/ha, shows a logarithmic decline to less than 25% dependency on its bacterial partner at > 125 kg nitrate/ha [7]. This research suggests that if chickpea crops are planted in moderate to high nitrogen containing agricultural soils, this negative feedback of nitrogen on N-fixation will lead to greater crop reliance on soil N. This will limit the ability of chickpea to enhance soil N for a following crop and reduces the sustainability of this particular legume in agriculture rotations. Therefore, it will become increasingly desirable to identify or breed genotypes that are less sensitive to high levels of plant-available N in soils to maintain the ability to form and acquire N from rhizobial symbiosis.

Understanding how genotype supports nodulation and N-fixation in chickpea is also important to maximise the benefit of N-fixation and reduce reliance on nitrogenous fertilisers. Surprisingly there have been few large-scale studies comparing chickpea nodulation and nitrogen production across multiple genotypes, with a recent review on this topic only citing two studies [8]. One study compared three American and three Middle Eastern chickpea varieties while the second compared 39 chickpea lines from a USDA collection [9, 10]. This latter study demonstrated that genotype vigour, as indicated by total plant biomass, was one of the most important factors affecting N-fixation, accounting for one third of the variation in N-fixation across the accessions [10]. This vigour was considered to be related to a larger plant biomass providing a stronger N sink [10]. Genotypes bred to exhibit increased disease resistance may also affect rhizobial symbiosis in chickpea. The productivity and profitability of chickpea crops are undermined each year by a range of root diseases, including Phytophthora root rot (PRR; caused by *Phytophthora medicaginis*) and Fusarium wilt (FW, caused by *Fusarium oxysporum* f.sp. *ciceris*). While the breeding of elite disease-resistant varieties is one of the most effective means used to combat diseases, especially root diseases [11, 12], genotype-based differences in nodulation and the proportion of plant-N derived from fixation were shown between PRR susceptible and moderately resistant chickpea varieties [13]. These differences were attributed to increases in the general basal defense processes of the moderately resistant varieties that, inadvertently, also compromised mutualist symbiosis [13]. Therefore, plant genotypes that support rhizobial symbiosis in chickpea need to be further investigated.

Until recently, sufficient genetic tools have not been available in chickpea to refine our understanding of the molecular bases of nodulation, but the release of 1 full and 29 re-sequenced genomes of chickpea [14] can facilitate the identification of genes related to the establishment of nodulation, and help understand how these interact with disease resistance response pathways, nitrogen perception and transport. The initial steps of nodule formation have been well studied in model legumes such as *Medicago truncatula* and *Lotus japonicus* [15]. From these systems, the general model is that upon perception of rhizobia, plant secondary metabolites, dominated by flavonoid compounds, are secreted by the host root and taken up by rhizobia leading to the production of secreted bacterial Nod-factors [16, 17]. These bacterial signals are perceived by host plant LysM receptor-like kinases, which induce a signalling cascade in the plant root called the Common Symbiosis Signalling Pathway (CSSP) and the induction of plant hormone pathways including cytokinin and auxin [18, 19]. Interruptions in these signalling steps can reduce the efficiency of nodulation or halt it altogether. Legumes also have a feedback signalling loop to control nodulation as, while an increased number of nodules may improve host plant N, excessive numbers of nodules negatively impact plant health due to the energy costs to the plant of both nodule formation and maintenance. Called the Autoregulation Of Nodulation (AON) feedback mechanism (reviewed by Ferguson et al. [20]), this pathway is regulated both by internal factors to the plant as well as by abiotic factors, particularly soil plant-available N [21–23]. It is likely that as we expand the number of model legume systems studied, that further genetic factors supporting nodulation will be found and, as such, the factors that affect these signalling events are an area of active research globally.

In this current study, we sought to further understand how *Mesorhizobium ciceri* (CC1192 [24]) inoculated chickpea genotypes differed in nodulation and nodule function. First, we consider how external factors (i.e. plant-available N) interact with genotype, as well as how intrinsic factors such as quantitative and qualitative disease resistance may alter nodulation potential. Using transcriptomic profiling of a core set of high- and low-nodulating chickpea genotypes, we then sought to understand what differential gene transcription supports high nodulation. Altogether, the results of this study inform our greater understanding of how host genotype and gene regulation affect symbiotic nodule formation and function and is an important step forward in identifying what genetic factors enable chickpea to benefit from rhizobia.

## Results

### Nodule formation in chickpea varies with soil conditions in a genotype-specific manner

Six genotypes of chickpea (Sonali, PBA HatTrick, Jimbour, Moti, Kyabra and Yorker) were grown in soils with varying plant-available nitrogen (N): paddock soil with nitrate-based fertilizer (highest plant-available N; HN), paddock soil without N fertilizer (moderate plant-available N; MN) and paddock soil cut with sand (2 parts sand to 1 part soil; lowest plant-available N; LN). We found that chickpea genotype significantly interacted with soil N treatment for nodule formation (*P* < 0.001; Table 1a). The differential impact of genotype on how soil N treatment affected nodulation was seen in varieties such as PBA HatTrick, which had no significant change in nodule numbers across the N treatments, whereas others like Moti and Yorker had significantly more nodules in MN compared to HN soil. Genotype root dry weight values also significantly interacted with soil N treatment (*P* < 0.001). PBA HatTrick had a higher root dry weight than Yorker in both LN and HN soil, but in the MN soil Yorker had a higher root dry weight than PBA HatTrick (Table 1b). Similarly, a genotype by soil N interaction was observed for nodule numbers normalised for differences in root dry weight (*P* < 0.05; Table 1c) whereby all genotypes had significant increases in the number of nodules/mg root from the HN to the MN soil. Conversely, nodule numbers/mg root significantly decreased from the MN to the LN soil treatments for all genotypes with the exception of Jimbour and Sonali, indicating genotype specific thresholds of soil plant available N required to support optimal nodulation in chickpea.

**Table 1:** a) Number of nodules per plant b) root dry weight (g) and c) number of nodules per g of root tissue from the six genotype experiment with three soil nitrogen treatments: high nitrogen (HN, paddock soil + N), medium nitrogen (MN, paddock soil no N) and low nitrogen (LN, sand:soil 2:1). Measurements taken 6 weeks post-inoculation with rhizobia. Standard error of the differences among means (SED) and least significant difference (LSD) values included

**Variety/media**	LN	MN	HN
	Sand:Soil	Paddock -N	Paddock + N
**a) Number of Nodules**			
**Jimbour**	23.3	49.3	16.0
**Kyabra**	24.7	40.0	33.7
**Moti**	35.0	64.3	34.0
**PBA HatTrick**	17.0	27.0	13.3
**Sonali**	28.7	42.3	22.3
**Yorker**	24.7	89.0	18.3
**SED**	7.90		
**LSD**	16.03		
**b) Root dry weight, mg**			
**Jimbour**	130.0	244.1	243.7
**Kyabra**	166.8	155.4	271.9
**Moti**	201.0	254.1	217.5
**PBA HatTrick**	293.3	134.9	295.0
**Sonali**	163.8	185.1	156.6
**Yorker**	182.8	300.6	187.7
**SED**	35.94		
**LSD**	73.4		
**c) Number Nodules/mg root**			
**Jimbour**	0.22	0.21	0.07
**Kyabra**	0.10	0.25	0.12
**Moti**	0.18	0.25	0.16
**PBA HatTrick**	0.09	0.20	0.05
**Sonali**	0.17	0.23	0.14
**Yorker**	0.14	0.30	0.10
**SED**	0.034		
**LSD**	0.070		

**Fig. 1:**
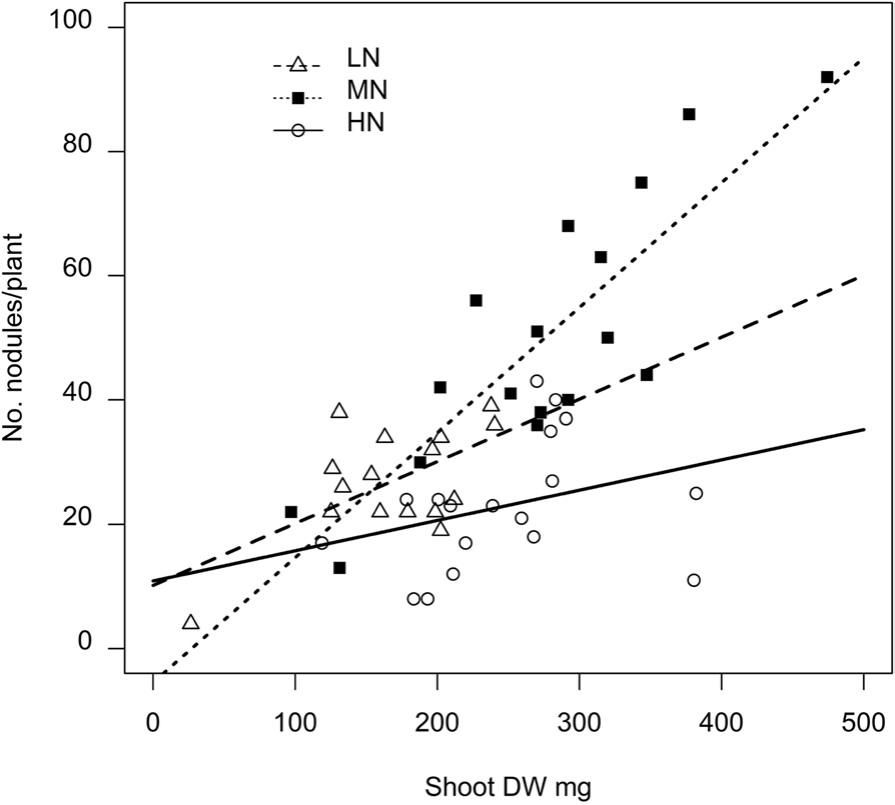
Chickpea shoot dry weight and soil N environment account for the number of nodules per plant. Linear regression plot of shoot dry weight (DW) for the six genotypes of chickpea grown with three levels of available N (HN (paddock soil + N), MN (paddock soil—N) and LN (sand:soil 2:1)) as predictors of the numbers of nodules per plant, *r*^2^ = 71.9, standard error of observations 9.97

The relationship between measured plant metrics (root and shoot dry weights, total plant weight and root:shoot ratio) and the number of nodules showed the strongest correlation coefficient (0.640) for shoot dry weight (Supplemental Table SM1). Further regression based evaluation, however, showed that shoot dry weight alone was not a strong predictor of nodule number (r^2^ 39.5), but that shoot dry weight in conjunction with soil N as factors accounted for a substantial amount of the variation in nodule numbers (r^2^ 71.9; Fig. 1). The N treatment with the smallest slope incline was the HN treatment, suggesting that nodule number is less crucial to plant dry weight in well fertilised conditions, while the MN treatment slope had the steepest incline.

### Genotype dependent nodule formation in chickpea is not significantly related to root disease resistance rating

**Fig. 2:**
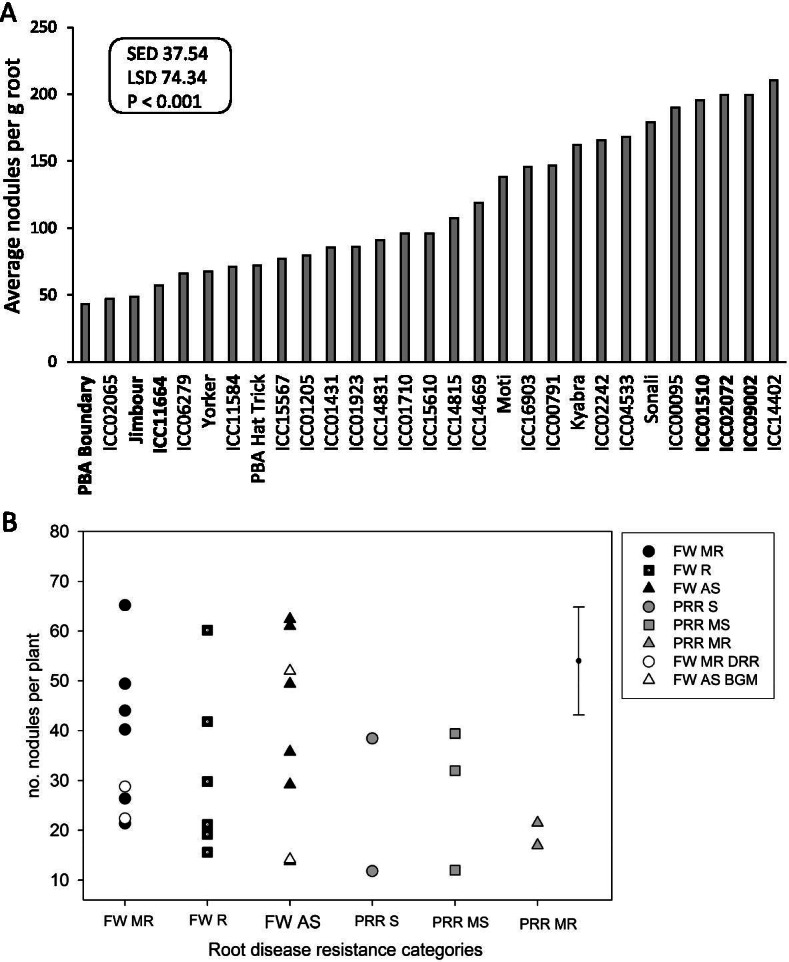
Number of nodules formed per plant varies by genotype but is not linked to root disease resistance categories. **A** Average number of nodules formed per g root for 29 genotypes of chickpea, with SED, LSD and *P* values presented, *N* = 5 per genotype. Genotypes in bold were selected for further transcriptomic study. **B** Genotype root disease resistance categories by the average number of nodules per plant for 22 lines with differing Fusarium wilt (FW) resistance categories (MR moderately resistant, R resistant, AS asymptomatic) and seven lines with differing Phytophthora root rot (PRR) resistance (S susceptible, MS moderately susceptible, MR moderately resistant). Two FW MR genotypes also have resistance to Dry Root Rot (DRR) and two FW AS genotypes also have resistance to Botrytis Grey Mould (BGM). The LSD value of 21.7 is presented as an error bar, *N* = 5. All specific genotypes values in each figure are provided in Supplementary Table SM2

Given the significant effect of chickpea genotype on nodule formation and previous research suggesting that breeding for root disease resistance may impact nodulation [13], we expanded our study to a set of 29 chickpea genotypes sourced from either Australia or India with differing root disease resistance levels and type (i.e. quantitative for PRR vs. qualitative for FW) and grown at a single N level, results for all genotypes and the corresponding root disease resistance categories are presented in Supplemental Table SM2. Similar to earlier experiments with fewer varieties, the average number of nodules per plant differed significantly among chickpea genotypes (Fig. 2a). Comparison of the genotypes according to their respective FW or PRR disease resistance categories showed that for the FW resistance category comparisons (i.e. based on qualitative resistance) there were no significant differences in nodulation parameters and nodule performance (*P* > 0.05; Fig. 2b, Table 2a). Similarly, for the PRR resistance category comparisons (i.e. based on quantitative resistance) there was no evidence for significant differences in nodulation parameters and nodule performance (*P* > 0.05), although it was notable that the PRR moderately resistant group had lower nodule/g root values than the other two categories at the *P* < 0.10 level (Table 2b). Comparisons of the two geographic sources of genotypes showed contrasting trends (Table 2c). For the plant nodulation parameter, the Indian genotypes as a group had significantly more nodules per plant than the Australian bred genotypes (*P* < 0.01), but for the nodule performance results the Australian bred genotypes had significantly higher real-time N-fixation values per nodule than the Indian sourced genotypes (*P* < 0.01). To allow comparison of the range of genotype values in each geographic source group to previous studies, the ratio between the largest and smallest value (L:S ratio) was calculated for each group (Supplemental Table SM2). The Australian and Indian groups had similar a L:S ratio for the number of nodules per plant (values of 3.3 and 4.7, respectively) and nodules/g root (values of 4.1 and 4.2, respectively), but for shoot and root weights the Indian group had double the L:S ratio of the Australian group. Relationships between nodulation and plant growth related variates were evaluated across all 29 genotypes, the strongest correlation (0.568, *P* < 0.05) was between shoot DW and the number of nodules per plant (Supplemental Table SM3).

**Table 2:** Results for disease resistance category and genotype source comparisons for 29 chickpea genotypes for the variates of number of nodules/plant, number of nodules/g root, and ethylene production per nodule (ppm/g) for comparisons of a) Fusarium wilt (FW) disease resistance categories: moderately resistant (MR); resistant (R) and asymptomatic (AS); b) Phytophthora root rot (PRR) disease resistance categories: susceptible (S); moderately susceptible (MS), and moderately resistant (MR); and c) genotype source group of Indian (IND) and Australian genotypes (AUS). *P* values, standard error of differences (SED) and least significant difference (LSD) values

**a) FW groups**						
	**FW MR**	**FW R**	**FW AS**	***P***	**SED**	**LSD**
**No. Nodules**	41.1	30.9	40.9	0.183	6.44	12.79
**No. nodules/g root**	123.4	110.6	129.4	0.596	19.88	39.45
**Ethylene/g nodule**	704	1545	1198	0.135	419.0	831.7
**b) PRR groups**						
	**PRR S**	**PRR MS**	**PRR MR**	***P***	**SED**	**LSD**
**No. Nodules**	25.1	28.9	19.3	0.253	6.24	12.76
**No. nodules/g root**	111.2	121.1	69.9	0.091	25.19	51.51
**Ethylene/g nodule**	1305	3064	2813	0.296	1255.0	2566.8
**c) Source group**						
	**IND**	**AUS**		***P***	**SED**	**LSD**
**No. Nodules**	38.0	25.3		0.006	4.51	8.93
**No. nodules/g root**	121.9	105.2		0.244	14.28	28.24
**Ethylene/g nodule**	1154	2452		0.001	390.5	772.6

### Chickpea genotypes exhibit similar transcriptomic profiles prior to rhizobial exposure

**Fig. 3:**
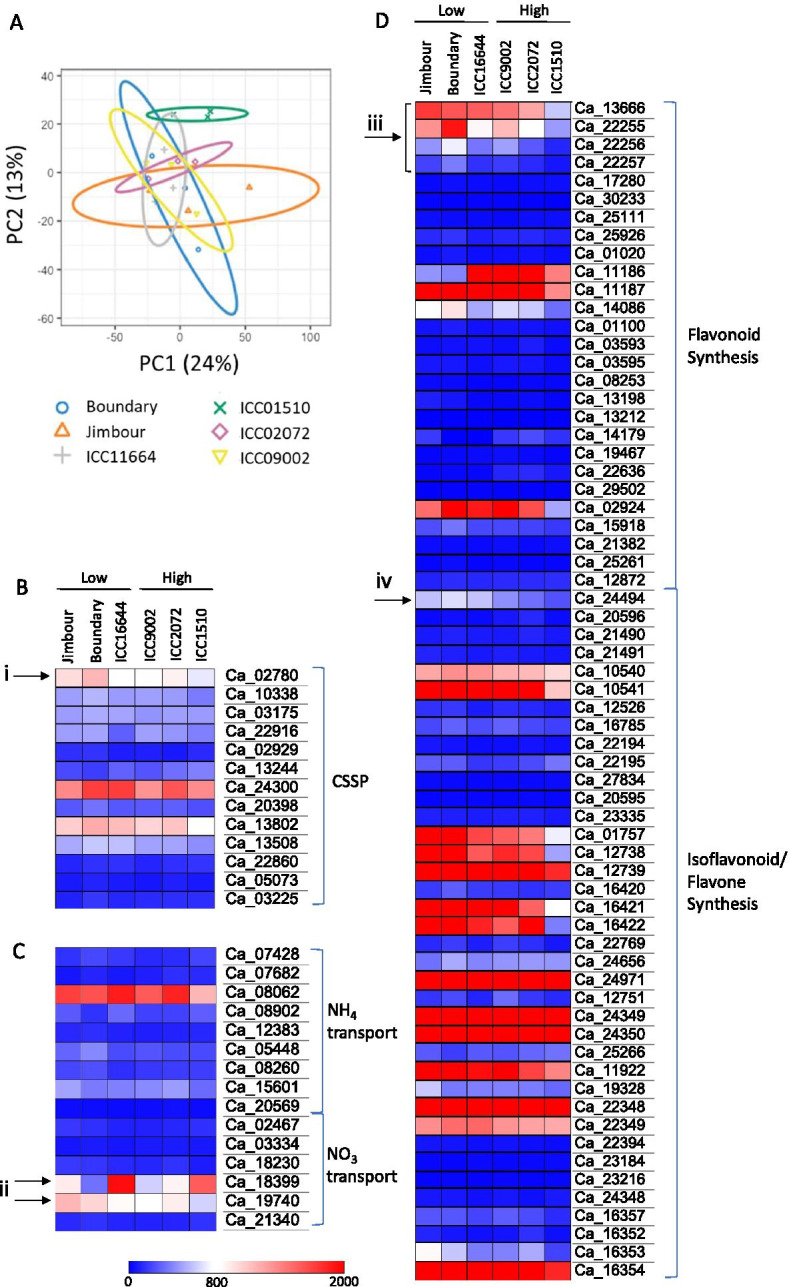
Six chickpea genotypes with differing nodulation ability have similar transcriptomic profiles. **A** PCA plot of normalized and rlog transformed RNAseq count data for the roots of 6 chickpea genotypes grown in sterile soil. 95% confidence ellipses for each genotype are shown. **B**-**D** Normalized gene expression for genes known to be involved in **B** the common symbiotic signaling pathway (CSSP), **C** nitrogen transport and **D** flavonoid biosynthesis pathways in the roots of six chickpea genotypes grown in sterile soil. Genes indicated with arrows are (i) DMI1 (ii) high-affinity nitrate transporters, (iii) chalcone-synthase homologues or (iv) a flavonoid methyltransferase

Six chickpea genotypes evenly split between high (ICC9002, ICC1510, ICC2072) and low (ICC11664, Jimbour, PBA Boundary) levels of nodulation were chosen based on the data above to study the difference in pre-symbiosis relative gene expression. Principle components analysis (PCA) of count data for all expressed genes (normalized and rlog transformed) from roots growing in the absence of rhizobia demonstrated that, with the exception of ICC1510 that partially separated from the group, all tested chickpea genotypes had a similar gene expression profile, with variability between genotypes being similar to that found within replicates (Fig. 3a). Similarly, relative gene expression in three different pathways associated with host rhizobial signalling and the initiation of nodulation—the CSSP, N-sensing/transport pathways, and flavonoid production pathways—was relatively consistent across all genotypes, regardless of each genotype’s nodulation potential (Fig. 3b-d). There are a few exceptions to this general observation, however. The genotypes forming more nodules also have lower levels of expression of chalcone synthase genes (Ca_13666, Ca_22255, Ca_22256, and Ca_22257; Fig. 3d), which are near the top of the flavonoid biosynthetic pathways, and a flavonoid methyltransferase (Ca_24494; Fig. 3d). Flavonoids, while being chemi-attractants for rhizobial bacteria, are also involved in plant defence pathways [25]. The homologue of DMI1 (Ca_02780), part of the CSSP, was most highly expressed in the two varieties forming the fewest nodules (Fig. 3b). Expression of the high affinity nitrate importers Ca_18399 and Ca_19740 (NRT2.1 and NRT1.2 homologues respectively) were also variable across the genotypes (Fig. 3c).

### Alterations to transcription in nodulation pathways following *M. ciceri* exposure do not mirror observed nodulation potentials in chickpea

**Fig. 4:**
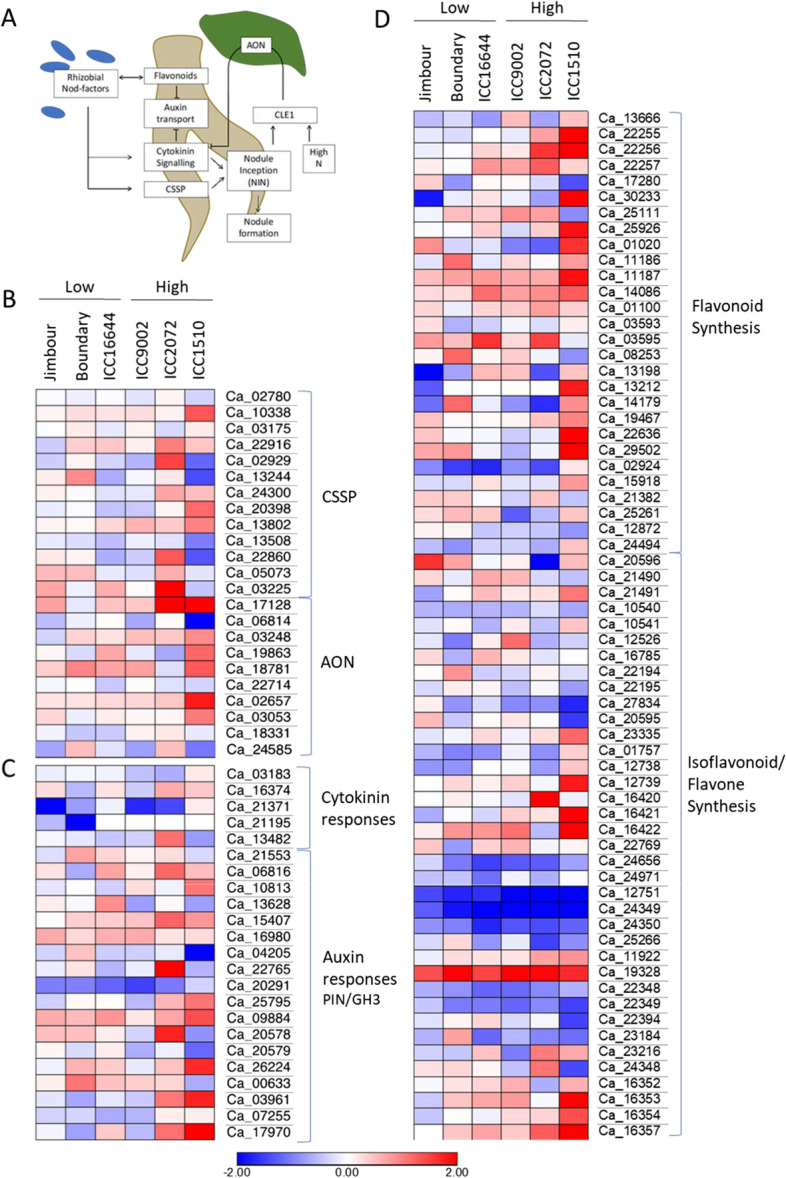
Six chickpea genotypes with differing nodulation ability have inconsistent changes in gene expression in known nodulation pathways after inoculation with rhizobia. **A** Simplified schematic of early signalling events and interactions upon perception of rhizobia by legume roots. **B**-**D** Log2 fold change in gene expression for genes involved in **B** the common symbiotic signalling pathway (CSSP) and autoregulation of nodulation (AON), **C** cytokinin and auxin hormone responses and **D** flavonoid biosynthesis pathways in the roots of six chickpea genotypes 3 days after inoculation with *M. ciceri*

Exposure to *M. ciceri* caused a number of genes to be differentially regulated in the chickpea roots 3 days post inoculation. Generally, ICC1510 was the most responsive genotype to the presence of the rhizobia with 2,383 genes being significantly differentially regulated (log2FC > 1, *p* > 0.05) compared to the sterile controls (Supplemental Figure 1). Early responses of legume roots to rhizobia have been well studied in model systems (Fig. 4a). Using specific genes in the chickpea CSSP and AON pathways, cytokinin and auxin responses, and flavonoid pathways, we considered the change in gene expression in genotypes with high or low nodulation after inoculation with rhizobia, compared to mock inoculated controls (Fig. 4b-d). High nodule forming ICC1510 and ICC2072 showed the most dramatic alterations in gene expression, particularly in the CSSP and AON pathways, however, the gene expression patterns showed high variability across genotypes with little to no link to nodulation potential. For example, Ca_22860, a homologue to Nod factor perception5 (NFR5), is up-regulated in ICC2072, but down-regulated in the two other high nodule forming genotypes ICC1510 and ICC9002. Nodule Inception (NIN; Ca_03225), which is a crucial transcriptional regulator controlling both nodule development and the AON pathway (Fig. 4a), also has inconsistent differential regulation among the genotypes, being upregulated in ICC2072, ICC16644 and Jimbour, but unchanged or downregulated in the other genotypes. Thus, there is no clear transcriptional pattern in the classical nodulation pathways distinguishing the response of high and low nodulation chickpea genotypes to rhizobia at this timepoint.

### Altered expression of genes in signalling, cellular localization and N compound transport characterize high nodulating chickpea varieties

**Fig. 5:**
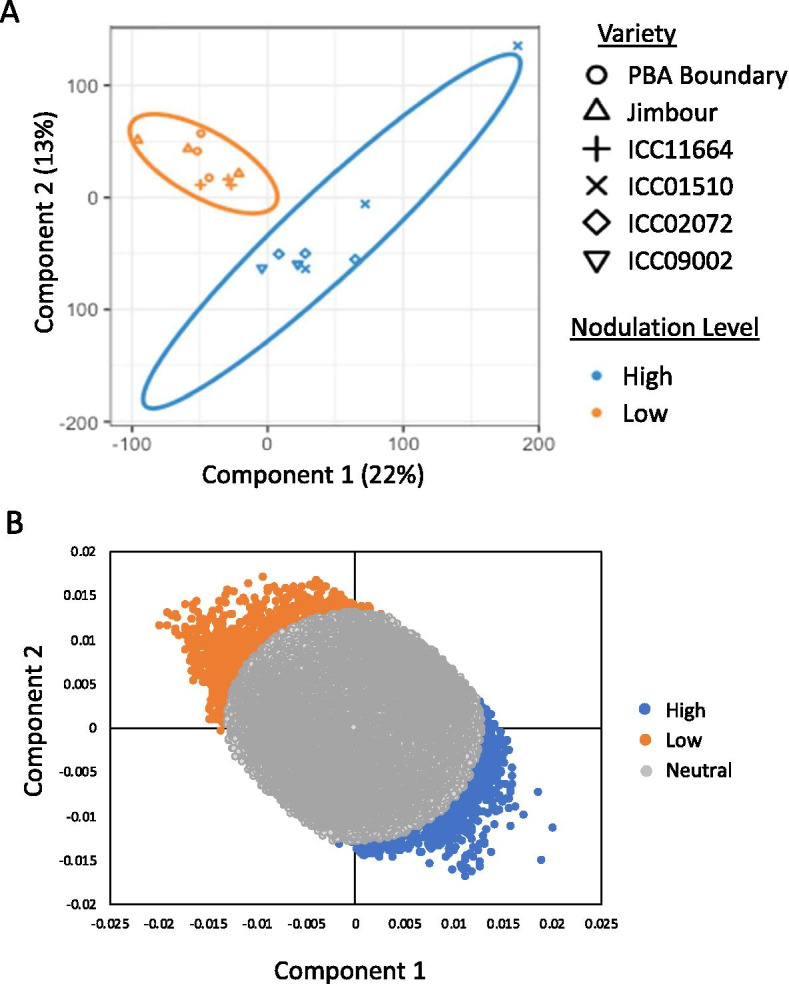
A subset of genes expressed in chickpea roots after inoculation with *Mesorhizobium ciceri* differentiate varieties with high nodule formation from those with low nodule formation. **A** PLS-DA plot of normalized and rlog transformed RNAseq count data for the roots of 6 chickpea genotypes 3 days after inoculation with *M. ciceri,* with groupings assigned based on nodulation ability (high or low). 95% confidence ellipses for each nodulation level are shown. **B** Plot of loadings values from **A** for all expressed genes. Genes having coordinates with *r* > 0.013 were considered to contribute to the separation of high or low nodulation varieties

Given the lack of genes in classical nodulation pathways clearly differentiating the response of high or low nodule forming varieties to rhizobia, we used a supervised method (PLS-DA) to determine those genes whose expression most delineated the two nodulation phenotypes (Fig. 5). This resulted in 533 genes associated with the high nodulation varieties and showing, on average, increased expression in high nodulation genotypes as compared to low nodulation genotypes. An additional 861 genes were associated with low nodulation varieties (Supplemental Table SM4). GO enrichment analysis of these two groups of genes resulted in no significantly enriched GO terms for those genes associated with low nodulation, however, 42 terms were significantly enriched in genes associated with high nodulation (*p* < 0.01). These enriched GO terms generally fall under the categories of signalling, cellular localization, N compound transport and macromolecule or cellular component assembly (Table 3).

**Table 3:** Significantly enriched GO terms (*p* < 0.01) found in the set of 533 genes most differentiating gene expression in high nodulating varieties from low nodulating varieties following inoculation with rhizobia based on PLS-DA analysis

**GO ID**	**Description**	***P* (Fisher)**
**Signalling**		
GO:0023052	signaling	0.00028
GO:0007264	small GTPase mediated signal transduction	2.90E-06
GO:0007154	cell communication	0.00041
GO:0035556	intracellular signal transduction	0.0004
GO:0007165	signal transduction	0.00026
GO:0051716	cellular response to stimulus	0.00164
GO:0050794	regulation of cellular process	0.00616
GO:0050789	regulation of biological process	0.00928
**Cellular localization**		
GO:0051649	establishment of localization in cell	4.60E-05
GO:0034613	cellular protein localization	7.70E-05
GO:0006913	nucleocytoplasmic transport	0.00211
GO:0046907	intracellular transport	4.50E-05
GO:0070727	cellular macromolecule localization	7.70E-05
GO:0051169	nuclear transport	0.00211
GO:0051641	cellular localization	4.90E-05
GO:0033036	macromolecule localization	0.00087
GO:0071702	organic substance transport	0.00246
GO:0006886	intracellular protein transport	7.70E-05
GO:0015031	protein transport	0.0005
GO:0008104	protein localization	0.0005
GO:0045184	establishment of protein localization	0.0005
**N compound transport**		
GO:0071705	nitrogen compound transport	0.00104
GO:0015833	peptide transport	0.00031
GO:0042886	amide transport	0.00031
**Cellular Components**		
GO:0006996	organelle organization	0.00017
GO:0044085	cellular component biogenesis	0.00031
GO:0016043	cellular component organization	0.00061
GO:0071840	cellular component organization or biogenesis	0.00069
GO:0022607	cellular component assembly	0.0002
**Macromolecule assembly**		
GO:0034622	cellular macromolecular complex assembly	2.20E-05
GO:0065003	macromolecular complex assembly	2.70E-05
GO:0006325	chromatin organization	0.0007
GO:0043933	macromolecular complex subunit organization	6.40E-05
GO:0071103	DNA conformation change	4.50E-05
GO:0006323	DNA packaging	2.70E-05
GO:0051276	chromosome organization	0.00025
GO:0034728	nucleosome organization	0.00028
GO:0006333	chromatin assembly or disassembly	0.00028
GO:0006334	nucleosome assembly	0.00028
GO:0065004	protein-DNA complex assembly	0.00028
GO:0031497	chromatin assembly	0.00028
GO:0071824	protein-DNA complex subunit organization	0.00028

## Discussion

Biological N-fixation by rhizobia in legume crops, like chickpea, is important for plant nutrition and a reduced reliance on nitrogenous fertilizers. However, as our study reaffirms, the extent of root nodule formation is dependent both on the genotype of the plant and its abiotic environment [8, 26]. We demonstrate here that nodulation varies in a genotype-dependent manner across 29 genotypes of chickpea, with genotype specific responses to N-availability being demonstrated in an experiment using a smaller set of 6 varieties. We show that nodule number, while linked to plant growth traits such as above ground biomass, is not consistently related to the disease resistance rating of the genotype. This latter observation was most marked in plants known to encode qualitative single pathogen resistance. Finally, using transcriptomic analysis during the early stages of rhizobial colonization, we show that genotypes with opposing levels of nodule formation, while having generally overlapping gene expression profiles, show a sub-set of genes involved in signalling and transport pathways that vary by nodulation level, suggesting a potential role for these genes in chickpea rhizobial symbiosis.

It is well established that endemic *Mesorhizobium* species can affect the extent of nitrogen fixation by inoculated species and the soil environment effects rhizobium populations [6, 27]. We therefore carried out genotype based studies in a sterile non-field environment to provide control over potentially confounding factors. These experiments demonstrated that, despite a strong effect of plant-available N in influencing nodulation in legumes, not all plant genotypes have the same response to changing N levels in the soil. Among the six genotypes tested, the number of nodules differed significantly, both between genotypes and across each of the three soil N levels tested. Similar genotype specific differences in nodulation and N-fixation have previously been reported when considering restricted sets of chickpea genotypes (*n* < 7) [9, 28]. Given the role of this symbiosis in supplementing plant N, it could be assumed that the lowest concentration of plant-available N in the soil would be most favourable to the establishment of rhizobial interaction with the plant host. However, in our study and in others, nodule numbers were found to be highest at moderate levels of N and lowest at either reduced or high N [29]. As the formation of nodules is an energetically expensive investment on the part of the plant, it is possible that at very low soil N concentration, the plant lacks the resources to form large numbers of nodules. The perception of nitrogen status and availability by the plant host is dependent on a number of sensory systems and signalling pathways, which in turn affect pathways necessary for nodulation [30]. For example, the presence of high levels of nitrate in soils can induce the expression of CLE peptides in legume roots, triggering the AON pathway, thereby suppressing nodulation [22, 23]. While we were unable to test these mechanisms, sensitivity differences among chickpea genotypes to soil plant-available N explained our observed genotype by soil N results. Further investigation of these pathways may result in the identification of genotypic markers that predict low plant sensitivity to nitrate and, therefore, may promote high levels of nodulation in agricultural fields with high residual levels of plant-available N.

In our previous work, in which we analysed a small set of moderately PRR resistant varieties, we demonstrated a possible link between quantitative resistance to PRR, lower nodule numbers and N-fixation [13]. This was consistent with other reports of how certain plant genotypes can simultaneously impact pathogenic and mutualistic symbioses [31–37]. However, while the moderately resistant PBA HatTrick again had lower nodulation values than PRR-susceptible Sonali in this study, other PRR susceptible/moderately susceptible varieties such as PBA Boundary formed fewer nodules again. When we considered different genotypes with a range of FW disease resistance categories, we found both exceptional and poor performance of specific genotypes within each resistance category in terms of the number of nodules formed on the root system. This lack of consistent trend between disease resistance and nodulation within the FW genotype set is likely due to the fact that resistance to *Fusarium oxysporum* f.sp. *ciceris* is qualitatively based on a small number of disease-specific genes [12]. Therefore, the likelihood that one of these genes may also negatively affect beneficial rhizobia was low and the finding that there was no consistent pattern between FW resistance and repression of nodulation potential was not unexpected. Together, these findings demonstrate that, while disease resistance may affect nodulation, it is not the only indicator of nodulation potential.

Improved selection methods for high nodulation legumes are required. The association between nodulation, or nodule N-fixation, and agronomic traits such as root weight and shoot weight has led to suggestions that breeders can use these in-direct indicators of N associated traits, to achieve ‘simultaneous improvement’ within a particular legume crop [8, 38]. Simultaneous improvement, defined as the selection of one plant trait linked pleiotropically to multiple valued agronomic phenotypes, as a method for varietal development is undoubtedly highly important, and would improve efficiency in breeding programs. It is also clear, however, that an improved understanding of the genetic basis of nodule formation and enhancement of N-fixation across a broader range of legume crops is required before more targeted selection techniques can easily be adopted during the breeding process. Chickpea, known to have relatively limited genomic diversity [39, 40] (Fig. 4), may seem unlikely to show enough differences in the establishment of rhizobial symbiosis to be a candidate crop where this may be achieved. However, our study using putatively genetically similar FW resistant genotypes, and another by Biabani et al. [10], using genetically diverse chickpea genotypes from 12 countries, both found large genotype based differences in nodulation.

In a consideration of gene expression in known nodulation pathways for genotypes with high or low nodulation potential, most genes were consistently expressed across the genotypes in the absence of rhizobia, however, there were notable differences between the high and low nodulating genotypes. For example, chalcone synthase gene expression was lower in genotypes forming the greatest number of nodules without rhizobia present. Chalcones sit at the top of the flavonoid pathway and play a role both in plant defence and as chemi-attractants for rhizobial bacteria [25] and proper balance of chalcone synthase activity is necessary for nodulation [41–43]. Upon addition of rhizobia, gene expression in known nodulation pathways was not altered in a manner consistent with nodulation potential. While this may, in part, stem from the early time point used, earlier research suggests that chickpea is already responding to rhizobia with altered gene transcription 3 days after inoculation in this system [13, 44]. The expression of Nodule inception (NIN) is down-regulated in two of the high nodulation genotypes, despite commonly being up-regulated upon perception of rhizobia in other model legume systems [45]. This transcription factor, while essential for nodule organogenesis, also sits at the head of the AON pathway and its activity can inhibit nodule initiation [46, 47]. Its activity is also tissue specific with distinct functions in root epidermis versus cortex [45], therefore it is possible that while the expression of the gene is generally down regulated in some genotypes, it could still be locally accumulated in specific tissues. Overall, however, the gene expression patterns seen in model legumes are not consistently reflected in the chickpea roots tested here. Thus, rather than one key pathway across which differential gene regulation describes differences in nodulation between genotypes, cross talk between multiple signalling pathways based on differences in a small set of genes may co-ordinately explain the observed nodulation patterns. Further examination of gene expression following inoculation with rhizobia resulted in a set of 533 genes associated with the high nodulation phenotype. Although we do not know at this time if and how each of these integrate into the known pathways supporting rhizobial symbiosis, they provide new avenues for future research and may provide the necessary markers for simultaneous improvement during the breeding of chickpea.

## Conclusions

Overall, the results of this paper give evidence to the possible scope for using genotypic screens in non-model crops such as chickpea to identify novel factors affecting nodule formation and function. Despite a narrow genetic pool, we have highlighted specific, discrete aspects by which chickpea genotype and transcriptional profiles impact rhizobial symbiosis. In future extending the results here may include consideration of the differences in the flavonoid profile produced by chickpea varieties with high and low nodule formation to identify specific compounds linked to nodule numbers. Further, the pathways controlled by the identified genes associated with the high nodulation should be refined with a larger set of genotypes and considered for their tractability as robust markers of nodulation potential. Together, results proceeding from the work described here as well as these proposed studies will generate the genetic framework necessary for the simultaneous improvement of non-model pulse crops.

## Methods

### Chickpea growth experiments

Six varieties of chickpea, Sonali, PBA HatTrick, Jimbour, Moti, Kyabra, and Yorker, were grown in three different soil types according to the methods of Plett et al. [13]. The source of all seed for the experiments was the New South Wales Department of Primary Industries led Pulse Breeding Australia chickpea breeding program (Tamworth, NSW, Australia). Briefly, seeds of each variety were surface sterilized with 10% bleach prior to planting in sterilized vermiculite. The seeds were allowed to germinate and grow for 2 weeks prior to transplanting into the test soils. Each plant was inoculated upon transplant with 10,000 CFU of *M. ciceri* (CC1192) and grown for another 6 weeks in growth chambers (18/10 °C day/night temperature; 12 h light cycle; BioChambers, Saskatchewan, Canada). The soils used for the experiments were all sterilized by gamma irradiation prior to use and included chickpea paddock soil with N fertilization (HN; 71.8 mg/kg NO_3_ + 7.5 mg/kg NH_4_), chickpea paddock soil without N fertilization (MN; 65 mg/kg NO_3_ + 7.5 mg/kg NH_4_) and paddock soil cut two times with sand (LN; 21.7 mg/kg NO_3_ + 2.5 mg/kg NH_4_). Plant available N was determined at harvest using KCl extraction as per Plett et al. [13]. Plants were fertilized three times after transplanting with an N free fertilizer and HN treatments were given an additional N fertilization treatment (3 × 1 mL of 0.1 M KNO_3_).

In the second series of experiments, 22 Desi lines were selected from International Crops Research Institute for the Semi-Arid Tropics (ICRISAT) mini-core collection of 211 accessions. The selections were based on the disease resistance information, seed colour and size, and origin information from Pande et al. [48]. To minimise potential origin based variation, all ICRISAT lines were chosen to originate from India. In addition only Desi lines and lines that were non-segregating for seed colour were selected. The final selection criteria was for Fusarium wilt resistance, with 6 lines rated as asymptomatic, 6 as moderately resistant and 6 as resistant to Fusarium wilt, and four lines with FW resistance that included multiple disease resistance as shown in Table SM-1. For comparison to the ICRISAT Fusarium wilt lines, from Australian sources, seven Australian bred Desi varieties were selected with differing PRR disease resistance ratings, two susceptible (Sonali, PBA Boundary), three moderately susceptible (Kyabra, Moti, Jimbour) and two moderately resistant (PBA HatTrick and Yorker). The chickpea plants were grown as for the first experiment but in one soil type (Sand:paddock soil 1:1, sterilized by gamma irradiation, 16 mg/kg NO_3_ + 1.2 mg/kg NH_4_) and given N free fertilizer only.

### Chickpea experiment harvest and analysis

At harvest for both experiments, chickpea root systems were rinsed of soil and the number of nodules per root system were counted. About 10–20 nodules were carefully removed from the root system, weighed and placed in a sealed 20-mL GC vial for an acetylene reduction assay to assess nodule activity through the production of ethylene as per Plett et al. [13]. The plant above and below ground portions were separated and dried at 40^0^C prior to weighing for biomass.

### RNA sequencing and analysis

Chickpea seeds from three genotypes forming low numbers of nodules (Jimbour, Boundary, ICC11664) and three genotypes with high nodulation (ICC9002, ICC2072 and ICC1510) were sterilized in 10% bleach, rinsed in sterile water and planted into vermiculite for 2 weeks as above. Seedlings were transplanted to sterilized soil (same as 29 genotype experiment) and acclimatised to the new soil for a day prior to inoculation. Actively growing cultures of *M. ciceri* (~ 10,000 CFU per plant) was applied to half of the chickpeas while a mock solution without rhizobia was applied to the other half. Chickpea roots were harvested and frozen in liquid nitrogen 3 days after addition of live *M. ciceri* cultures or mock controls.

RNA was extracted from the chickpea roots (3 biological replicates per condition) using the Isolate II RNA Plant kit (Bioline, London, U.K.) according to manufacturer’s instructions. RNA libraries were constructed using the SENSE total RNA-Seq library prep kit (Lexogen, Vienna, Austria) and NovaSeq sequencing was performed at the Ramaciotti Centre for Genomics (University of New South Wales, Sydney, Australia). Raw reads from the RNA-sequencing were trimmed and mapped to the *C. arietinum* genome (desi type—ICC4958; [49]) using CLC Workbench v.10. Genes that were not expressed or lowly expressed (< 5 counts on average across each set of 3 replicates) were eliminated from the data set. Raw gene count data was normalized using the DESeq2 package in R (DeSEQ2 v.1.24.0; Love et al. [50]; R v.1.2.1335; [51]). For differential gene expression analysis, log2 fold change was calculated for the rhizobia inoculated chickpea roots as compared to the uninoculated control roots. Adjusted *p*-values were calculated using a Benjamini–Hochberg FDR correction. Genes up- or down- regulated more than 2 times and with *p* < 0.05 were considered significantly differentially regulated. Gene annotations were assigned based on the desi-type chickpea annotation [52].

Identification of genes in specific flavonoid production, nodulation or nitrogen transportation pathways was accomplished either by directly searching the *C. arietinum* genome annotation, or by BLAST analysis of corresponding protein sequences from the model legume *M. truncatula* or from *A. thaliana* against the *C. arietinum* (ICC4958) proteome [same ref as above] (Supplemental Table SM5). Determination of flavonoid biosynthesis enzymes was based off of Gifford et al. [16]. *M. truncatula* and *A. thaliana* protein sequences were obtained from an open commons database [53].

### Statistical analyses

Data from the two chickpea controlled environment experiments were analysed with ANOVA, using an appropriate transformation prior to the analysis, to stabilise the variances. Means comparison tests used the Fisher’s LSD method. Correlations between all variates in both studies were completed, in addition selected variates were compared with linear regression. For the 29-genotype experiment, additional comparisons of values between the Indian and Australian genotype groups were made using the ratio between the highest and lowest value in each group for each variate was calculated and called the largest to smallest (L:S) ratio. All analyses were carried out in Genstat.

Principal components analysis (PCA) and partial least squares discriminate analysis (PLS-DA) was conducted in R using the mixOmics package [54]. Input transcriptomic data was first normalized and rlog transformed using the R DeSEQ2 package. Loadings data for all genes in the PLS-DA analysis were plotted and those genes falling outside of a radius of 0.013 from the origin were considered to be contributing towards the separation of high and low nodulation phenotypes. GO enrichment analyses were conducted in R using the topGO package [55] and GO annotations based on the *C. arietinum* (ICC4958) genome. GO terms were assigned as significantly enriched based on a classic Fisher test (*p* < 0.01). Heat maps of normalized count data and log2 fold change data were generated using the Morpheus online tool [56].

## Supplementary Information

**Additional file 1: Supplemental Figure SM1.** Number of genes significantly differentially expressed in chickpea roots with differing nodulation after inoculation with *Mesorhizobium ciceri*. Number of genes significantly up- (blue) or down- (orange) regulated (log2FC >1, *p* < 0.05) in the roots of six chickpea genotypes three days after inoculation with *M. ciceri *as compared to sterile controls. Average number of nodules formed by plants at harvest are indicated (grey line). **Supplemental Table SM1.** Correlation coefficient results for the six genotype experiment with soil nitrogen treatments for measurements after four weeks of plant height, leaf and branch number, number of nodules, number of nodules per g of root, root dry weight (DW), root to shoot DW ratio, shoot DW and total plant DW. *indicates *P* < 0.05 for a two-sided test of correlations different from zero. **Supplemental Table SM2.** Varietal comparison results for the controlled environment study of 29 chickpea genotypes with a range of root disease resistance ratings, for the variates: number of nodules (no. nod.); root (g.), shoot (g.), total plant (tot. pl, g) dry weights (DW); root:shoot (R:S) DW ratio per plant; number of nodules/g of root (no. nod/g); and nodule ethylene production (eth ppm per g nod). Root disease resistance abbreviations Fusarium wilt (FW), Phytophthora root rot (PRR), resistant (R), asymptomatic (AS), moderately resistant (MR), Dry root rot (DRR), Botrytis grey mould (BGM), moderately susceptible (MS), susceptible (S). *P* values, standard error of differences (SED) and least significant difference (LSD) values from ANOVA are included. For the Indian (ICC lines) and Australian (all non-ICC lines; PRR) lines the ratio between the highest and lowest value (L:S ratio) for each variate is also presented. *Genotypes studied for transcriptomic responses. **Supplemental Table SM3.** Correlation coefficient results for the 29 genotype experiment for measurements of number of nodules, number of nodules per g of root, average weight of nodules, ethylene production per gram of nodule, root dry weight (DW) shoot DW, root:shoot DW ratio and total plant DW. *indicates *P* < 0.05 for a two-sided test of correlations different from zero. **Supplemental Table SM4.** List of chickpea genes contributing to the separation of high and low nodulating chickpea genotypes in PLS-DA analysis (based on loadings coordinates with *r* > 0.013) of gene expression 3 days after inoculation with *Mesorhizobium ciceri*. Loadings coordinates on components 1 and 2 and radius are indicated, along with the gene and GO annotation. **Supplemental Table SM5.** List of chickpea genes and annotations in known pathways for early signalling and responses to rhizobia. Annotations were assigned based on either protein BLAST to known *Medicago truncatula* or *Arabidopsis* homologues if indicated, or gene assignments based on the *Cicer arietinum* (ICC4958) genome. * taken from Plett et al. 2016 [13] **genes identified based on Gifford et al. 2018 [16].

## Data Availability

The transcriptomic datasets generated and analysed during the current study along with count data tables are available at GEO submission number GSE162321: https://www.ncbi.nlm.nih.gov/geo/query/acc.cgi?acc=GSE162321.

## Abbreviations

AON: Autoregulation of nodulation
CFU: Colony Forming Units
CSSP: Common symbiotic signalling pathway
DW: Dry weight
FW: Fusarium wilt
GO: Gene ontology
LSD: Least significant difference
N: Nitrogen
PCA: Principal Components analysis
PLS-DA: Partial Least Squares Discriminate analysis
PRR: Phytophthora root rot
SED: Standard error of difference
