# Supplementary material for: Chickpea shows genotype-specific nodulation responses across soil nitrogen environment and root disease resistance categories
Source: BMC Plant Biol. 2021 Jul 1;21:310. doi: 10.1186/s12870-021-03102-6 (PMC8247157; doi:10.1186/s12870-021-03102-6)
Supplement: Supplementary file 1 — Additional file 1: Supplemental Figure SM1. Number of genes significantly differentially expressed in chickpea roots with differing nodulation after inoculation with Mesorhizobium ciceri. Number of genes significantly up- (blue) or down- (orange) regulated (log2FC >1, p < 0.05) in the roots of six chickpea genotypes three days after inoculation with M. ciceri as compared to sterile controls. Average number of nodules formed by plants at harvest are indicated (grey line). Supplemental Table SM1. Correlation coefficient results for the six genotype experiment with soil nitrogen treatments for measurements after four weeks of plant height, leaf and branch number, number of nodules, number of nodules per g of root, root dry weight (DW), root to shoot DW ratio, shoot DW and total plant DW. *indicates P < 0.05 for a two-sided test of correlations different from zero. Supplemental Table SM2. Varietal comparison results for the controlled environment study of 29 chickpea genotypes with a range of root disease resistance ratings, for the variates: number of nodules (no. nod.); root (g.), shoot (g.), total plant (tot. pl, g) dry weights (DW); root:shoot (R:S) DW ratio per plant; number of nodules/g of root (no. nod/g); and nodule ethylene production (eth ppm per g nod). Root disease resistance abbreviations Fusarium wilt (FW), Phytophthora root rot (PRR), resistant (R), asymptomatic (AS), moderately resistant (MR), Dry root rot (DRR), Botrytis grey mould (BGM), moderately susceptible (MS), susceptible (S). P values, standard error of differences (SED) and least significant difference (LSD) values from ANOVA are included. For the Indian (ICC lines) and Australian (all non-ICC lines; PRR) lines the ratio between the highest and lowest value (L:S ratio) for each variate is also presented. *Genotypes studied for transcriptomic responses. Supplemental Table SM3. Correlation coefficient results for the 29 genotype experiment for measurements of number of nodules, number of nodules [file 12870_2021_3102_MOESM1_ESM.pdf]

Supplementary Material

Chickpea shows genotype-specific nodulation responses across soil nitrogen environment and root disease resistance categories

Krista L Plett, Sean L. Bithell, Adrian Dando, Jonathan M Plett

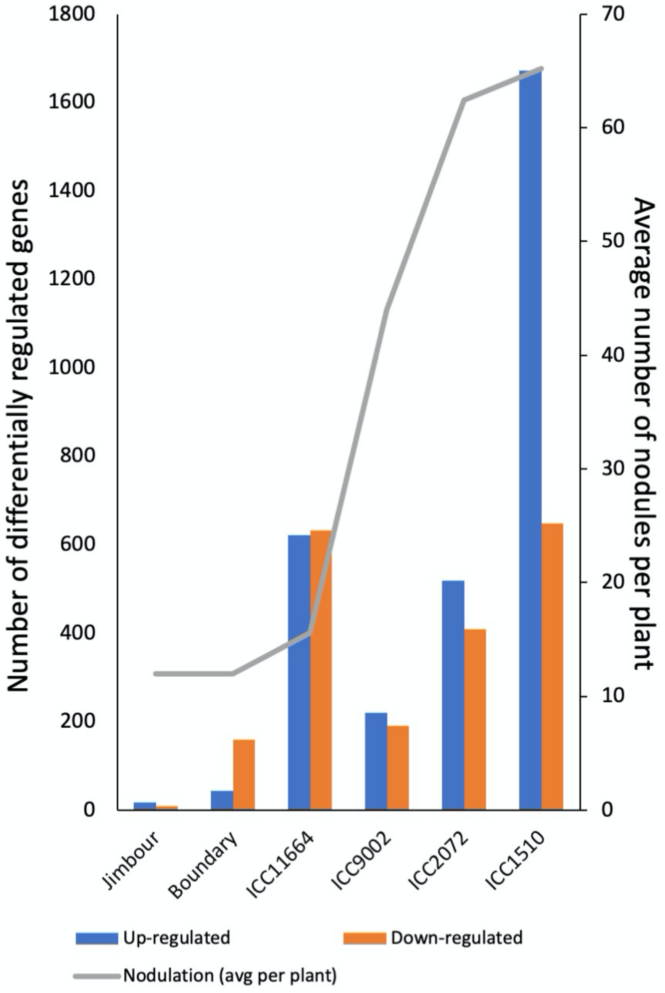

Supplemental Figure 1 Number of genes significantly differentially expressed in chickpea roots with differing nodulation after inoculation with *M. ciceri*. Number of genes significantly up- (blue) or down- (orange) regulated (log2FC >1, $p < 0.05$) in the roots of six chickpea varieties three days after inoculation with *M. ciceri* as compared to sterile controls. Average number of nodules formed by plants at harvest are indicated (grey line).

Supp. Info. section

Table SM1: Correlation coefficient results for the six genotype experiment with soil nitrogen treatments for measurements after four weeks of plant height, leaf and branch number, number of nodules, number of nodules per g of root, root dry weight (DW), root to shoot DW ratio, shoot DW and total plant DW. *indicates P < 0.05 for a two-sided test of correlations different from zero

Height_cm	-								
Leaves	0.5703*	-							
Branches	0.2728	0.4386*	-						
Nodules	0.4162*	0.2314	0.4714*	-					
Nod/g root	0.4719*	0.1462	0.3255*	0.8294*	-				
Root DW	0.1166	0.4217*	0.4616*	0.3883*	-0.113	-			
Root:sh_ratio	-0.659*	-0.6867*	-0.3893*	-0.3829*	-0.4544*	-0.1568	-		
Shoot_DW	0.581*	0.5728*	0.6193*	0.6401*	0.3341*	0.7022*	-0.5814*	-	
Total_DW	0.418*	0.551*	0.5983*	0.5782*	0.1588	0.8943*	-0.4365*	0.9466*	-
	Height_cm	Leaves	Branches	Nodules	Nod/g root	Root DW	Root:sh_ratio	Shoot_DW	

Table SM2 Varietal comparison results for the controlled environment study of 29 chickpea genotypes with a range of root disease resistance ratings, for the variates: number of nodules (no. nod.); root (g.), shoot (g.), total plant (tot. pl, g) dry weights (DW); root:shoot (R:S) DW ratio per plant; number of nodules/g of root (no. nod/g); and nodule ethylene production (eth ppm per g nod). Root disease resistance abbreviations Fusarium wilt (FW), Phytophthora root rot (PRR), resistant (R), asymptomatic (AS), moderately resistant (MR), Dry root rot (DRR), Botrytis grey mould (BGM), moderately susceptible (MS), susceptible (S). P values, standard error of differences (SED) and least significant difference (LSD) values from ANOVA are included. For the Indian (ICC lines) and Australian (all non-ICC lines; PRR) lines the ratio between the highest and lowest value (L:S ratio) for each variate is also presented. *Genotypes studied for transcriptomic responses.

	name	Root disease resistance	no. nod.	root DW	no. nod/g root	shoot DW	tot pl DW	R:S DW ratio	eth ppm per g nod
1	ICC00095	FW R	60.2	0.342	190	0.370	0.712	0.92	2008
2	ICC00791	FW R	41.8	0.346	146.7	0.327	0.674	1.03	1822
3	ICC01205	FW AS	29.2	0.353	79.8	0.316	0.669	1.17	982
4	ICC01431	FW MR	40.2	0.476	85.5	0.438	0.913	1.11	477
5	ICC01510*	FW MR	65.2	0.337	195.6	0.422	0.759	0.80	691
6	ICC01710	FW/DRR MR	28.8	0.356	95.8	0.381	0.737	0.88	1880
7	ICC01923	FW MR	26.4	0.314	86.1	0.366	0.679	0.86	746
8	ICC02065	FW AS	13.8	0.286	47	0.200	0.485	1.46	544
9	ICC02072*	FW AS	62.4	0.289	199.4	0.410	0.699	0.74	3012
10	ICC02242	FW/DRR MR	22.4	0.194	165.8	0.256	0.451	1.20	2489
11	ICC04533	FW AS/BGM	52	0.330	168	0.316	0.646	1.07	1610
12	ICC06279	FW AS/BGM	14.2	0.231	66.1	0.198	0.429	1.15	156

13	ICC09002*	FW MR	44	0.222	199.6	0.254	0.476	0.90	833
14	ICC11584	FW R	21.2	0.156	138	0.240	0.396	0.65	1119
15	ICC11664*	FW R	15.6	0.262	57.2	0.298	0.559	0.88	113
16	ICC14402	FW AS	49.4	0.513	96.1	0.374	0.887	1.37	635
17	ICC14669	FW AS	35.8	0.310	118.8	0.415	0.725	0.77	826
18	ICC14815	FW R	19.2	0.217	107.3	0.271	0.488	0.80	390
19	ICC14831	FW R	29.8	0.321	91.1	0.466	0.786	0.70	855
20	ICC15567	FW MR	21.4	0.299	77.2	0.255	0.554	1.19	841
21	ICC15610	FW MR	49.4	0.513	96.1	0.374	0.887	1.37	635
22	ICC16903	FW AS	61	0.417	145.6	0.564	0.98	0.75	31
23	JIMBOUR*	PRR MS	12	0.260	48.5	0.301	0.561	0.90	4020
24	KYABRA	PRR MS	39.4	0.242	162.3	0.391	0.633	0.64	3845
25	MOTI	PRR MS	32	0.229	138.1	0.313	0.542	0.74	1519
26	PBABOUNDARY*	PRR S	11.8	0.247	43.2	0.300	0.547	0.81	343
27	PBAHATTRICK	PRR MR	17	0.219	72.3	0.278	0.497	0.71	1910
28	SONALI	PRR S	38.4	0.223	179.1	0.343	0.566	0.65	2267
29	YORKER	PRR MR	21.5	0.321	67.5	0.358	0.679	0.90	3716
		SED	10.96	0.0559	37.54	0.0556	0.1006	0.193	1161.9
		LSD	21.71	0.1107	74.34	0.1102	0.1992	0.382	2301
		P	<0.001	<0.001	<0.001	<0.001	<0.001	<0.001	0.002
	ICC L:S ratio		4.7	3.3	4.2	2.8	2.5	2.3	97.2
	PRR L:S ratio		3.3	1.5	4.1	1.4	1.3	1.4	11.7

Table SM3: Correlation coefficient results for the 29 genotype experiment for measurements number of nodules, number of nodules per g of root, average weight of nodules, ethylene production per gram of nodule, root dry weight (DW) shoot DW, root:shoot DW ratio and total plant DW. *indicates $P < 0.05$ for a two-sided test of correlations different from zero.

	No. nodules	No. nod/g root	Av. wgt nodules	Ethylene/g nodule	Root DW	Shoot DW	Root:shoot ratio	
No. nodules	-							
No. nod/g root	0.753*	-						
Av. wgt nodules	0.0441	-0.0579	-					
Ethylene/g nodule	0.0597	0.0545	-0.0466	-				
Root DW	0.4128*	-0.1741*	0.1296	0.1014				
Shoot DW	0.5676*	0.2291*	0.2369*	0.1927*	0.600*	-		
Root:shoot ratio	-0.1271	-0.4087*	-0.1161	-0.0051	0.4188*	-0.372*	-	
Total plant DW	0.5507*	0.0376	0.2067*	0.166	0.8875*	0.9012*	0.0127	-

Supplemental Table SM4 List of chickpea genes contributing to the separation of high and low nodulating chickpea genotypes in PLS-DA analysis (based on loadings coordinates with r > 0.013) of gene expression 3 days after inoculation with M. ciceri. Loadings coordinates on components 1 and 2 and radius are indicated, along with the gene and GO annotation and the average normalised count data for each genotype.

Chickpea Gene ID	Component 1	Component 2	Radius	Gene Annotation	GO Annotation	Normalised Count Data - High Nodulating Genotypes			Normalised Count Data - Low Nodulating Genotypes		
**Low nodulation associated**						ICC01510	ICC02072	ICC09002	ICC11664	Boundary	Jimbour
Ca_16765	-0.0118	0.0066	0.0135	17.6 kDa class II heat shock protein		3.1	0.0	0.0	8.5	6.0	5.7
Ca_23991	-0.0131	0.0071	0.0149	1-aminocyclopropane-1-carboxylate synthase 9	GO:0003824 GO:0009058 GO:0030170	0.0	0.0	5.8	6.8	6.0	9.5
Ca_14260	-0.0115	0.0082	0.0142	26S proteasome regulatory subunit 4 homolog A [Glycine max]		1.1	0.0	6.5	8.0	18.0	4.5
Ca_06743	-0.0165	0.0054	0.0173	2-phosphoglycolate phosphatase 1	GO:0008152 GO:0016791	4.0	10.9	10.6	21.2	27.8	27.0
Ca_06116	-0.0119	0.0111	0.0162	31-kDa RNA binding protein	GO:0000166 GO:0003676	3.1	0.0	0.0	2.8	3.1	5.6
Ca_18763	-0.0026	0.0142	0.0144	4-hydroxy-3-methylbut-2-enyl diphosphate reductase	GO:0019288 GO:0055114	31.0	7.6	0.0	8.4	12.6	12.1
Ca_03643	-0.0123	0.0071	0.0142	AAR2 protein family		6.7	7.8	4.9	6.9	15.5	20.2
Ca_19675	-0.0079	0.0109	0.0135	ABC transporter family protein	GO:0005524 GO:0006810 GO:0016021 GO:0042626 GO:0055085	82.9	54.7	48.9	65.8	103.2	89.2
Ca_26669	-0.0013	0.0130	0.0131	ABC transporter family protein	GO:0005524 GO:0006810 GO:0016021 GO:0042626 GO:0055085	54.0	49.6	22.3	49.6	34.0	41.1
Ca_19189	-0.0110	0.0144	0.0181	ABC transporter family protein (ATP-binding component)	GO:0000166 GO:0005524 GO:0006810 GO:0016021 GO:0016887 GO:0017111 GO:0042626	350.8	287.7	206.7	306.6	424.2	416.6
Ca_21748	-0.0139	0.0057	0.0150	ABC transporter family protein (ATP-binding component)	GO:0000166 GO:0005524 GO:0006810 GO:0016021 GO:0016887 GO:0017111 GO:0042626 GO:0055085	174.0	260.9	241.2	369.0	334.9	331.3
Ca_16529	-0.0039	0.0125	0.0131	ABC transporter-related	GO:0005524 GO:0016020 GO:0016887	407.8	221.1	140.8	209.1	281.3	343.4
Ca_28830	-0.0116	0.0067	0.0134	Acetylornithine deacetylase n=19 Tax=Enterobacteriaceae RepID=E1SI75_PANVC	GO:0005737 GO:0006526 GO:0008152 GO:0008270 GO:0008777 GO:0016787 GO:0050897	0.0	0.0	0.0	1.2	0.0	3.2
Ca_06572	-0.0102	0.0146	0.0178	Acid phosphatase/vanadium-dependent haloperoxidase-related protein		5.4	4.2	0.0	5.0	8.5	7.3
Ca_12068	-0.0037	0.0132	0.0137	actin-related protein 8	GO:0005515	234.8	105.2	129.4	183.7	149.2	163.1
Ca_29111	-0.0117	0.0110	0.0161	adenylate kinase 1	GO:0004017 GO:0005524 GO:0006139 GO:0016776 GO:0019205	2.6	0.0	0.0	7.2	5.5	3.0
Ca_14843	-0.0110	0.0083	0.0138	adiponectin receptor protein 2-like isoform X3 [Glycine max]	GO:0016021	22.6	28.5	18.2	26.9	35.2	36.6
Ca_14029	-0.0118	0.0057	0.0131	ADP-ribosylation factor GTPase-activating protein AGD10		424.0	600.0	514.3	680.6	663.6	667.0
Ca_03343	-0.0046	0.0146	0.0153	aldehyde oxidase 1		330.9	259.5	263.5	284.4	299.0	323.6
Ca_28655	-0.0086	0.0122	0.0149	Aldo/keto reductase n=6 Tax=Enterobacteriaceae RepID=E6WBR3_PANSA		8.8	3.0	0.0	12.8	5.7	5.3
Ca_28720	-0.0154	0.0051	0.0162	Aldoketomutase n=2 Tax=Enterobacteriaceae RepID=U4VZB3_ENTAG		37.9	58.2	41.2	85.2	103.8	92.3
Ca_22897	-0.0104	0.0086	0.0135	aldose 1-epimerase [Glycine max]	GO:0003824 GO:0005975 GO:0016853 GO:0019318 GO:0030246	2.9	0.0	9.0	8.7	6.9	10.2
Ca_06931	-0.0132	0.0004	0.0132	alpha carbonic anhydrase 4	GO:0004089 GO:0006730 GO:0008270	18.3	44.8	47.0	67.9	74.9	63.7
Ca_27126	-0.0128	0.0043	0.0135	alpha/beta hydrolase domain-containing protein 13-like [Glycine max]		4.5	8.3	11.5	12.7	17.5	29.5
Ca_06342	-0.0135	0.0076	0.0155	alpha-amylase-like	GO:0003824 GO:0004556 GO:0005509 GO:0005975	0.0	0.0	0.0	0.0	6.3	6.8
Ca_15554	-0.0110	0.0076	0.0133	aluminum-activated malate transporter 9	GO:0015743	120.1	114.4	137.5	153.9	158.6	129.0
Ca_09267	-0.0124	0.0067	0.0141	amidase 1	GO:0016884	250.1	259.7	242.1	322.5	327.9	276.5
Ca_08260	-0.0073	0.0123	0.0143	ammonium transporter 2	GO:0008519 GO:0015696 GO:0016020 GO:0072488	267.8	136.5	96.9	168.4	317.8	210.2
Ca_05693	-0.0135	0.0018	0.0136	ankyrin repeat-containing protein At5g02620-like isoform X3 [Glycine max]		11.6	13.1	33.8	54.9	53.3	38.3
Ca_25147	-0.0122	0.0099	0.0157	annexin 8	GO:0005509 GO:0005544	84.9	146.0	151.0	252.6	159.9	211.8
Ca_29392	-0.0141	0.0088	0.0166	AP2 domain class transcription factor		0.0	0.0	0.0	3.6	1.7	4.6
Ca_15962	-0.0050	0.0128	0.0138	Arabidopsis phospholipase-like protein (PEARLI 4) family		112.8	76.8	59.1	74.2	92.1	111.0
Ca_28699	-0.0156	0.0078	0.0175	Arginase family protein n=4 Tax=Actinomyces RepID=J3A8Z7_ACTNA	GO:0004053 GO:0006525 GO:0016813 GO:0046872	2.5	5.2	0.0	8.3	12.3	12.5
Ca_30054	-0.0159	0.0131	0.0206	arginine N-methyltransferase	GO:0006479 GO:0008168	2.3	0.0	0.0	5.3	4.0	7.4
Ca_28048	-0.0169	0.0096	0.0195	armadillo repeat only 1	GO:0005488	0.0	0.0	0.0	1.9	5.3	7.0
Ca_29202	-0.0125	0.0085	0.0151	asparagine-tRNA ligase	GO:0000166 GO:0004812 GO:0004816 GO:0005524 GO:0005737 GO:0006418 GO:0006421	6.6	9.2	0.0	9.6	14.1	13.8
Ca_18236	-0.0128	0.0081	0.0152	aspartic proteinase-like protein 2-like isoform X2 [Glycine max]		3.7	0.0	0.0	2.5	6.7	7.0
Ca_06499	-0.0115	0.0071	0.0135	ataxin-10-like [Glycine max]	GO:0005488	150.2	133.0	102.8	135.3	193.9	182.3
Ca_01831	-0.0117	0.0103	0.0156	ATP binding		216.8	248.2	212.9	229.6	304.9	290.6
Ca_03676	-0.0133	0.0098	0.0165	ATP binding / kinase/ protein serine / threonine kinase n=3 Tax=Vitis vinifera RepID=C5DB47_VITVI	GO:0004672 GO:0004674 GO:0005515	238.5	260.3	255.6	362.3	292.9	452.5
Ca_16294	-0.0052	0.0150	0.0159	ATP binding microtubule motor family protein	GO:0003777 GO:0005524 GO:0005871 GO:0007018 GO:0008017	258.4	184.3	147.0	192.6	231.1	219.6
Ca_21763	-0.0138	0.0043	0.0144	ATP binding microtubule motor family protein	GO:0003777 GO:0005524 GO:0005871 GO:0007018 GO:0008017	324.5	399.8	338.9	378.4	494.4	491.4
Ca_29670	-0.0120	0.0109	0.0162	ATP binding microtubule motor family protein	GO:0003777 GO:0005524 GO:0005871 GO:0007018 GO:0008017	5.6	5.0	3.8	8.9	10.0	10.6
Ca_29762	-0.0017	0.0147	0.0148	ATP binding microtubule motor family protein n=1 Tax=Theobroma cacao RepID=UPI00042B89EE	GO:0003777 GO:0005524 GO:0005871 GO:0007018 GO:0008017	11.9	0.0	0.0	7.6	3.5	0.0
Ca_01803	-0.0094	0.0134	0.0164	ATP binding/protein serine/threonine kinase [Glycine max]		113.7	106.6	89.6	118.6	146.3	136.7

Ca_03601	-0.0061	0.0116	0.0131	ATP binding/protein serine/threonine kinase [Glycine max]		432.0	283.8	247.6	260.2	496.9	385.7
Ca_08899	-0.0048	0.0124	0.0133	ATP binding/protein serine/threonine kinase [Glycine max]		606.3	407.7	482.1	718.3	400.1	591.0
Ca_00962	-0.0123	0.0046	0.0131	ATP-binding ABC transporter	GO:0000166 GO:0005524 GO:0016020 GO:0016887 GO:0017111	64.9	51.3	36.8	84.3	97.3	84.5
Ca_25485	-0.0087	0.0112	0.0142	ATP-binding ABC transporter	GO:0000166 GO:0005524 GO:0016020 GO:0016887 GO:0017111	168.0	200.6	123.9	164.0	214.8	213.7
Ca_12213	-0.0165	0.0089	0.0188	ATP-binding cassette transport family protein n=1 Tax=Populus trichocarpa RepID=B9HZ05_POPTR	GO:0005524 GO:0006810 GO:0016021 GO:0016887 GO:0042626 GO:0055085	19.4	39.4	13.7	77.6	52.1	110.5
Ca_03510	-0.0087	0.0158	0.0180	ATP-binding/protein serine/threonine kinase [Glycine max]	GO:0004672	2721.3	1350.1	1545.8	1824.5	2724.9	2486.8
Ca_19486	-0.0074	0.0113	0.0135	ATP-binding/protein serine/threonine kinase [Glycine max]	GO:0004672 GO:0004674 GO:0005524 GO:0006468 GO:0016772	365.0	215.7	371.7	398.7	386.0	371.1
Ca_19911	-0.0092	0.0171	0.0194	ATP-binding/protein serine/threonine kinase [Glycine max]	GO:0004672 GO:0004674 GO:0005524 GO:0006468 GO:0016772	97.8	73.6	58.3	90.3	114.4	91.5
Ca_21561	-0.0046	0.0134	0.0142	ATP-binding/protein serine/threonine kinase [Glycine max]	GO:0004672 GO:0004674 GO:0005524 GO:0006468 GO:0016772	70.0	29.4	45.8	57.8	42.9	57.5
Ca_22276	-0.0037	0.0134	0.0139	ATP-dependent DNA helicase PcrA n=1 Tax=Clostridium sp. D5 RepID=F0YVJ8_9CLOT	GO:0003677 GO:0004003 GO:0005524 GO:0016787	301.8	118.9	144.3	210.4	217.2	175.7
Ca_25938	-0.0156	0.0110	0.0192	ATP-dependent zinc metalloprotease FTSH protein	GO:0000166 GO:0004222 GO:0005524 GO:0006508 GO:0016020 GO:0017111	28.0	29.6	27.2	37.2	42.0	43.6
Ca_08783	-0.0002	0.0132	0.0132	auxilin-like protein 1-like [Glycine max]		656.6	281.0	252.6	319.8	363.4	367.0
Ca_22292	-0.0141	0.0068	0.0156	auxin canalisation protein		6.7	4.8	4.0	15.1	9.0	16.6
Ca_23480	-0.0085	0.0119	0.0146	Auxin efflux carrier family protein	GO:0016021 GO:0055085	179.1	180.0	165.8	219.1	208.1	202.7
Ca_05936	-0.0181	0.0125	0.0219	auxin transport protein (BIG)	GO:0004842 GO:0005515 GO:0008270	1.0	0.0	0.0	6.0	4.0	5.9
Ca_11786	-0.0040	0.0143	0.0149	auxin-responsive protein IAA32-like [Glycine max]	GO:0005634 GO:0006355 GO:0046983	44.5	7.3	4.6	15.7	30.7	11.6
Ca_12153	-0.0033	0.0128	0.0133	B3 domain-containing protein Os01g0905400-like isoform X2 [Glycine max]	GO:0003677	156.1	50.0	42.8	62.2	96.7	93.5
Ca_02906	-0.0125	0.0039	0.0131	B3 domain-containing transcription factor LEC2-like [Glycine max]	GO:0003677	14.1	20.4	19.8	28.3	50.1	37.1
Ca_20994	-0.0120	0.0064	0.0136	BAX inhibitor 1		28.6	34.6	41.2	50.4	96.1	53.4
Ca_00716	-0.0124	0.0072	0.0143	B-cell receptor-associated 31-like	GO:0005783 GO:0006886 GO:0016021	40.6	23.3	22.0	37.4	76.5	75.1
Ca_19282	-0.0051	0.0147	0.0156	BEACH domain-containing protein lvsC-like isoform X7 [Glycine max]	GO:0005515	680.8	140.8	167.5	265.1	297.3	460.6
Ca_05035	-0.0152	0.0059	0.0163	BEL1-like homeodomain protein 8-like isoform X2 [Glycine max]		38.2	47.1	56.9	65.6	123.9	84.3
Ca_09872	-0.0064	0.0115	0.0131	beta glucosidase 11	GO:0004553 GO:0005975	148.7	65.5	124.5	143.1	114.7	130.3
Ca_18663	-0.0057	0.0152	0.0163	beta glucosidase 13	GO:0004553 GO:0005975	90.8	58.3	49.0	81.5	61.0	81.0
Ca_07867	-0.0133	0.0090	0.0160	beta-fructofuranosidase	GO:0004553 GO:0005975	59.0	50.1	65.8	89.0	104.3	86.7
Ca_13878	-0.0069	0.0146	0.0161	beta-galactosidase 16	GO:0004553 GO:0005975	385.0	262.5	237.1	363.2	336.8	369.2
Ca_28689	-0.0112	0.0081	0.0138	Binding-protein-dependent transport systems inner membrane component n=12 Tax=Enterobacteriaceae RepID=E6WB25_PANSA	GO:0005215 GO:0006810 GO:0016020	0.0	0.0	0.0	6.7	0.0	2.3
Ca_28452	-0.0168	0.0110	0.0201	Biofilm development protein YmgB/AriR n=2 Tax=Pantoea RepID=J2UGN8_9ENTR	GO:0042710 GO:0071229	1.9	0.0	0.0	6.1	5.4	7.3
Ca_22107	-0.0005	0.0130	0.0130	BRCA1-associated protein-like [Glycine max]	GO:0008270	72.0	0.0	0.0	4.5	5.3	8.4
Ca_07403	-0.0070	0.0119	0.0138	breast cancer associated RING 1	GO:0003677 GO:0004842 GO:0005515 GO:0005634 GO:0006281 GO:0008270	160.7	184.4	128.4	178.0	177.7	180.0
Ca_19017	-0.0088	0.0109	0.0140	bromodomain-containing protein 9-like [Glycine max]	GO:0005515	332.8	171.9	334.3	295.1	391.2	408.0
Ca_21846	-0.0035	0.0139	0.0143	BTB/POZ domain-containing protein At5g47800-like isoform X1 [Glycine max]		270.4	138.9	155.4	184.6	218.7	191.0
Ca_03284	-0.0040	0.0130	0.0136	bZIP transcription factor	GO:0003700 GO:0006355 GO:0043565	91.3	86.4	62.8	117.3	68.7	91.4
Ca_25179	-0.0042	0.0138	0.0144	c(\2\""	GO:0008152 GO:0016791	1206.7	104.4	266.5	443.7	701.7	411.1
Ca_25181	-0.0035	0.0125	0.0130	c(\2\""	GO:0008152 GO:0016791	907.4	86.7	185.3	288.4	609.6	256.5
Ca_29031	-0.0128	0.0072	0.0146	c(\ABC transporter\""	GO:0004970 GO:0005215 GO:0005234 GO:0006810 GO:0016020	0.0	0.0	0.0	0.0	3.2	4.2
Ca_00432	-0.0096	0.0129	0.0161	c(\ADP\""	GO:0005215 GO:0005743 GO:0006810 GO:0055085	97.7	89.0	58.7	91.1	102.6	135.1
Ca_27415	-0.0119	0.0074	0.0140	c(\alpha-1\""	GO:0000166 GO:0003676 GO:0016866 GO:0030244	3.4	4.8	0.0	8.4	6.1	10.6
Ca_07564	-0.0102	0.0135	0.0169	c(\ATP-dependent RNA helicase\""	GO:0000166 GO:0003676 GO:0004386 GO:0005524 GO:0008026 GO:0017111	456.9	332.7	283.4	1305.2	381.3	406.5
Ca_28582	-0.0156	0.0096	0.0183	c(\Biotin-protein ligase\""		1.3	0.0	0.0	2.9	3.5	5.4
Ca_29562	-0.0111	0.0104	0.0152	c(\CAP (Cysteine-rich secretory proteins\""		0.0	0.0	0.0	6.8	0.0	3.4
Ca_28432	-0.0084	0.0111	0.0139	c(\Carbohydrate kinase\""	GO:0005975 GO:0016301	10.4	5.7	0.0	8.0	9.4	8.5
Ca_06173	-0.0099	0.0094	0.0137	c(\copper ion-binding laccase\""	GO:0005507 GO:0016491 GO:0046274 GO:0048046 GO:0052716 GO:0055114	810.5	573.0	1326.6	1531.6	981.3	1511.2
Ca_01785	-0.0131	0.0048	0.0139	c(\Core-2/I-branching beta-1\""	GO:0008375 GO:0016020	103.1	78.6	203.5	374.9	157.0	352.2
Ca_20720	-0.0033	0.0136	0.0140	c(\cytochrome P450\""	GO:0005506 GO:0016705 GO:0020037 GO:0055114	433.8	105.7	108.9	161.4	276.3	184.4
Ca_19369	-0.0060	0.0150	0.0162	c(\disease resistance protein (TIR-NBS-LRR class)\""	GO:0005515 GO:0006952 GO:0007165 GO:0043531	124.1	71.4	70.0	94.0	107.8	99.0
Ca_26648	-0.0036	0.0126	0.0131	c(\disease resistance protein (TIR-NBS-LRR class)\""	GO:0005515	220.8	139.1	130.0	148.6	174.0	196.7
Ca_15300	-0.0080	0.0121	0.0145	c(\DNA binding protein\""	GO:0003676 GO:0003677 GO:0046983	90.5	82.3	65.7	81.2	108.6	98.7
Ca_28896	-0.0133	0.0084	0.0158	c(\DNA glycosylase/AP lyase\""	GO:0003676 GO:0003677 GO:0003684 GO:0003824 GO:0003906 GO:0006281 GO:0006284	4.1	0.0	0.0	8.8	4.6	7.2
Ca_22631	-0.0134	0.0076	0.0154	c(\formyltetrahydrofolate deformylase\""	GO:0006189 GO:0008864 GO:0009058 GO:0016742	7.8	13.3	7.0	15.6	19.2	23.9
Ca_22357	-0.0045	0.0125	0.0133	c(\fructose-1\""	GO:0005975 GO:0042132 GO:0042578	327.6	216.2	215.9	287.2	375.2	370.1
Ca_22022	-0.0111	0.0095	0.0147	c(\glucan endo-1\""		0.0	0.0	0.0	4.0	1.3	3.6
Ca_29430	-0.0128	0.0039	0.0134	c(\glucan endo-1\""	GO:0004553 GO:0005975	2.5	5.4	5.2	7.2	11.1	8.6

Ca_18302	-0.0144	0.0015	0.0145	c(\glycerol-3-phosphate dehydrogenase\""	GO:0004367 GO:0005737 GO:0005975 GO:0006072 GO:0009331 GO:0016491	19.2	49.1	45.2	58.7	68.3	90.7
Ca_28969	-0.0123	0.0092	0.0153	c(\Marine sediment metagenome DNA\""	GO:0003676 GO:0003677 GO:0004803 GO:0006313 GO:0015074	3.8	7.3	0.0	11.1	5.5	10.1
Ca_10792	-0.0127	0.0051	0.0137	c(\mitochondrial phosphate carrier protein 3\""		15.6	37.1	17.1	29.0	80.1	60.7
Ca_00877	-0.0116	0.0087	0.0145	c(\Mitochondrial protein\""		3.5	0.0	0.0	13.2	15.7	4.6
Ca_28802	-0.0131	0.0088	0.0158	c(\N-acylhomoserine lactone synthase\""	GO:0007165	5.2	0.9	4.5	13.8	5.3	11.5
Ca_05678	-0.0118	0.0056	0.0131	c(\Non-specific lipid-transfer protein\""	GO:0006869 GO:0008289	1.2	1.8	2.8	6.4	10.0	3.8
Ca_17583	-0.0164	0.0110	0.0198	c(\Non-specific lipid-transfer protein\""	GO:0006869 GO:0008289	3.1	0.0	0.0	10.4	5.1	8.2
Ca_18294	-0.0155	0.0076	0.0172	c(\Non-specific lipid-transfer protein\""	GO:0006869 GO:0008289	4.1	0.0	7.5	10.6	14.2	11.3
Ca_21894	-0.0127	0.0053	0.0138	c(\nuclear factor Y\""	GO:0003677 GO:0005622 GO:0005634 GO:0006355 GO:0043565 GO:0046982	4.8	7.7	5.3	12.4	7.8	11.7
Ca_06590	-0.0139	0.0058	0.0151	c(\Nutrient reservoir\""	GO:0045735	1.9	0.0	3.7	8.1	3.9	10.6
Ca_28864	-0.0117	0.0060	0.0131	c(\Oligopeptide ABC transporter\""	GO:0005215 GO:0006810 GO:0016020	3.3	8.4	0.0	11.3	10.0	10.3
Ca_28589	-0.0086	0.0115	0.0144	c(\P pilus assembly protein\""	GO:0007155 GO:0009289	3.3	0.0	0.0	9.1	3.1	0.0
Ca_25971	-0.0107	0.0122	0.0162	c(\Polyprotein\""		34.1	32.6	33.2	53.7	50.5	43.4
Ca_06256	-0.0122	0.0154	0.0196	c(\Retrotransposon protein\""	GO:0003676 GO:0008270	4.4	0.0	0.0	4.3	3.1	7.1
Ca_23869	-0.0118	0.0103	0.0156	c(\Retrotransposon protein\""	GO:0003676 GO:0008270	0.0	0.0	0.0	2.7	2.1	3.0
Ca_24314	-0.0124	0.0057	0.0137	c(\Retrotransposon protein\""		23.4	19.6	17.6	27.8	48.6	39.5
Ca_24392	-0.0024	0.0132	0.0134	c(\Retrotransposon protein\""		73.6	38.9	22.4	34.0	52.0	39.7
Ca_24672	-0.0117	0.0083	0.0144	c(\Retrotransposon protein\""		4.4	0.4	0.0	7.7	4.6	4.2
Ca_24770	-0.0062	0.0159	0.0171	c(\Retrotransposon protein\""		843.4	329.0	413.9	704.2	538.6	551.9
Ca_25080	-0.0121	0.0063	0.0136	c(\Retrotransposon protein\""		3.0	0.0	0.0	3.5	8.8	21.6
Ca_25233	-0.0134	0.0095	0.0164	c(\Retrotransposon protein\""		18.4	19.1	29.9	37.3	43.2	36.5
Ca_25700	-0.0068	0.0136	0.0152	c(\Retrotransposon protein\""	GO:0003676	58.4	46.9	33.0	49.8	57.7	57.8
Ca_25846	-0.0013	0.0140	0.0141	c(\Retrotransposon protein\""	GO:0003676 GO:0015074	130.8	31.8	19.5	59.9	53.3	38.1
Ca_26592	-0.0115	0.0068	0.0134	c(\Retrotransposon protein\""		0.0	0.0	0.0	0.0	12.0	8.3
Ca_28066	-0.0115	0.0063	0.0131	c(\Retrotransposon protein\""		0.0	1.8	0.0	0.0	7.3	5.0
Ca_13239	-0.0166	0.0064	0.0178	c(\ribonuclease II\""		1.4	5.3	3.1	8.2	8.3	10.3
Ca_14053	-0.0137	0.0101	0.0170	c(\serine protease inhibitor\""		5.3	0.0	3.2	7.2	5.2	12.2
Ca_08976	-0.0133	0.0008	0.0133	c(\Serine/Threonine-kinase\""		8.4	10.2	14.5	23.2	22.2	18.5
Ca_10417	-0.0109	0.0091	0.0142	c(\serine/threonine-protein kinase STN8\""		1.4	0.0	0.0	10.4	14.5	0.0
Ca_23627	-0.0138	0.0092	0.0166	c(\Site-specific recombinase\""	GO:0000150 GO:0003677 GO:0006310	4.1	6.3	0.0	6.8	17.3	12.6
Ca_16261	-0.0127	0.0071	0.0145	c(\thiamine thiazole synthase 2\""	GO:0006950 GO:0009228	60.7	43.1	78.0	46.2	314.5	656.6
Ca_13698	-0.0137	0.0103	0.0171	c(\threonyl-tRNA synthetase\""	GO:0000166 GO:0004812 GO:0004829 GO:0005524 GO:0005737 GO:0006418 GO:0006435	71.7	59.7	69.0	85.9	115.0	94.0
Ca_29057	-0.0147	0.0022	0.0149	c(\Transcriptional regulator\""	GO:0003700 GO:0006355	8.0	6.7	9.2	15.1	15.3	24.7
Ca_12058	-0.0043	0.0128	0.0135	c(\transmembrane protein\""		83.0	38.2	35.1	51.0	46.1	65.5
Ca_28502	-0.0152	0.0110	0.0187	c(\Type VI secretion system effector\""		4.1	2.7	0.0	6.2	5.9	7.8
Ca_23988	-0.0134	0.0014	0.0135	c(\ubiquitin-associated/TS-N domain protein\""		12.6	9.0	39.5	49.0	42.5	46.2
Ca_25175	-0.0011	0.0144	0.0145	c(\Ulp1 protease family\""		104.8	28.4	29.3	38.9	50.9	39.4
Ca_04867	-0.0016	0.0142	0.0143	c(\uncharacterized protein LOC102663625\""		93.3	33.7	23.6	38.4	42.0	50.0
Ca_13752	-0.0052	0.0137	0.0146	c(\WEB family protein At5g16730\""		175.9	80.6	101.5	118.7	152.3	135.4
Ca_19269	-0.0128	0.0093	0.0158	c(\WEB family protein At5g16730\""		10.6	8.8	8.9	16.5	11.5	16.2
Ca_13078	-0.0094	0.0091	0.0131	c(\zinc finger CCCH domain protein\""	GO:0005515 GO:0046872	207.6	152.7	158.8	353.0	241.6	191.6
Ca_17507	-0.0163	0.0133	0.0211	c(\zinc finger\""		2.3	0.0	0.0	5.9	9.3	8.1
Ca_03761	-0.0072	0.0138	0.0155	Calcineurin-like metallo-phosphoesterase superfamily protein	GO:0016787	102.3	49.1	38.5	81.5	74.5	70.3
Ca_14183	-0.0066	0.0124	0.0140	Calcium-binding EF-hand family protein n=1 Tax=Theobroma cacao RepID=UPI00042B2CE2	GO:0005509	832.2	303.9	380.0	569.4	523.0	912.4
Ca_06784	-0.0077	0.0153	0.0172	calcium-binding mitochondrial carrier protein SCaMC-1-like [Glycine max]	GO:0005509 GO:0055085	58.0	14.9	10.6	43.8	65.5	25.7
Ca_04616	-0.0084	0.0100	0.0131	calcium-dependent lipid-binding (CaLB domain) family protein		17.9	12.0	7.3	34.5	16.9	16.3
Ca_24006	-0.0139	0.0060	0.0152	calcium-dependent protein kinase 26	GO:0005509	0.0	0.0	2.5	3.1	10.4	4.2
Ca_23029	-0.0171	0.0104	0.0200	Calmodulin binding protein-like		4.7	13.3	13.5	55.7	30.4	32.3
Ca_11686	0.0011	0.0131	0.0131	calmodulin-binding family protein		160.0	80.3	75.6	88.3	98.3	93.9
Ca_13457	-0.0045	0.0131	0.0139	carbonic anhydrase 2	GO:0004089 GO:0008270 GO:0015976	38.0	24.6	16.9	23.1	39.9	29.7
Ca_19071	-0.0161	0.0103	0.0191	CASP-like protein 7 [Glycine max]		92.1	127.8	110.7	154.0	200.6	167.9
Ca_28571	-0.0124	0.0103	0.0161	catalytic LigB subunit of aromatic ring-opening dioxygenase family	GO:0006725 GO:0008198 GO:0008270 GO:0016491 GO:0016701 GO:0055114	1.4	0.0	0.0	6.0	0.0	6.6
Ca_07191	-0.0080	0.0105	0.0132	cathepsin B-like cysteine protease	GO:0004197 GO:0006508 GO:0008234 GO:0050790	586.9	290.9	618.9	917.5	495.9	649.2
Ca_07634	-0.0090	0.0104	0.0138	cation calcium exchanger 4	GO:0016021 GO:0055085	327.4	318.7	353.2	388.8	402.3	385.5
Ca_02616	-0.0130	0.0099	0.0164	CBS domain-containing protein with a domain of unknown function (DUF21)		72.7	29.6	43.3	65.5	103.8	84.0
Ca_24823	-0.0028	0.0129	0.0132	CBS domain-containing protein with a domain of unknown function (DUF21)		118.1	100.0	75.2	118.3	101.3	82.0
Ca_18980	-0.0191	0.0115	0.0223	cell number regulator-like protein		0.0	0.0	0.0	4.8	5.7	6.9
Ca_05153	-0.0135	0.0080	0.0157	Cellular nucleic acid-binding protein n=1 Tax=Medicago truncatula RepID=G7LCT8_MEDTR	GO:0003676 GO:0008270	0.0	1.3	0.0	3.4	6.0	5.7
Ca_24039	-0.0164	0.0139	0.0215	Cellular nucleic acid-binding protein-like protein n=1 Tax=Medicago truncatula RepID=G7L3B2_MEDTR	GO:0003676 GO:0008270	2.5	0.0	0.0	6.0	6.6	8.8
Ca_08575	-0.0137	0.0044	0.0144	cellulose synthase like G3	GO:0008610 GO:0016020 GO:0016491 GO:0016760 GO:0030244 GO:0055114	3.7	9.6	8.3	11.3	13.4	16.1
Ca_17411	-0.0142	0.0063	0.0155	Chaperone DnaJ-domain superfamily protein		25.6	22.1	18.0	37.1	32.0	41.7
Ca_20600	-0.0135	0.0030	0.0138	Chaperone DnaJ-domain superfamily protein		13.8	16.0	59.7	153.6	36.8	175.1
Ca_23494	-0.0038	0.0151	0.0155	chitinase domain-containing protein 1 isoform X2 [Glycine max]	GO:0004553 GO:0004568 GO:0005975 GO:0006032	176.6	119.6	118.6	152.3	144.1	135.6
Ca_00816	-0.0148	0.0053	0.0158	Chloroplast heat shock protein-binding protein n=1 Tax=Coffea canephora RepID=Q1W7A9_COFCA	GO:0005506 GO:0009055 GO:0051536	16.7	26.9	33.9	58.2	45.5	57.1

Ca_10182	-0.0139	0.0104	0.0174	chorismate synthase	GO:0004107 GO:0009073	3.3	0.0	0.0	6.8	9.9	8.8
Ca_05576	-0.0034	0.0156	0.0160	chromodomain-helicase-DNA-binding protein 1-like isoform X2 [Glycine max]	GO:0003676 GO:0003677 GO:0004386 GO:0005524	172.7	84.5	76.1	103.4	125.5	114.8
Ca_09216	-0.0113	0.0080	0.0138	chromodomain-helicase-DNA-binding protein 1-like isoform X2 [Glycine max]	GO:0003677 GO:0005524	4.5	0.0	4.2	5.4	6.4	10.5
Ca_15169	-0.0033	0.0154	0.0157	chromosome-associated kinesin-related	GO:0003777 GO:0005871 GO:0007018	164.0	123.4	100.8	141.4	128.0	133.0
Ca_16627	-0.0002	0.0137	0.0137	cinnamoyl coa reductase 1		40.7	14.8	7.0	19.8	10.6	18.6
Ca_13522	-0.0080	0.0123	0.0147	clathrin coat assembly protein AP180-like [Glycine max]	GO:0005543 GO:0005545 GO:0030118 GO:0030276 GO:0048268	191.9	105.1	75.8	110.9	173.0	233.9
Ca_29205	-0.0148	0.0092	0.0174	Conserved uncharacterized protein n=1 Tax=Erwinia billingiae (strain Eb661) RepID=D8MNC4_ERWBE		0.0	0.0	0.0	4.3	6.7	3.3
Ca_28499	-0.0130	0.0106	0.0168	Conserved uncharacterized protein n=1 Tax=Erwinia billingiae (strain Eb661) RepID=D8MWD8_ERWBE	GO:0003824	0.8	0.0	0.0	38.6	4.1	4.7
Ca_28590	-0.0135	0.0095	0.0165	Curli production assembly/transport component CsgG n=5 Tax=Pseudomonas RepID=I4Y2Z3_9PSED	GO:0015031 GO:0030288 GO:0042597	6.5	5.3	3.2	15.8	9.6	8.6
Ca_10966	-0.0064	0.0120	0.0136	Cyclophilin-like peptidyl-prolyl cis-trans isomerase family protein	GO:0003755 GO:0006457	88.5	61.7	43.3	79.2	70.9	81.1
Ca_28486	-0.0121	0.0067	0.0138	Cyclopropane-fatty-acyl-phospholipid synthase	GO:0008610	0.0	0.0	0.0	2.2	0.0	1.8
Ca_24524	-0.0153	0.0097	0.0181	cysteine-rich receptor-like protein kinase 2-like isoform X4 [Glycine max]		2.7	0.0	0.0	7.1	4.9	11.5
Ca_03112	-0.0162	0.0081	0.0181	Cytochrome P450 superfamily protein	GO:0005506 GO:0016705 GO:0020037 GO:0055114	37.8	43.5	71.6	103.0	124.9	177.1
Ca_07817	-0.0073	0.0144	0.0162	Cytochrome P450 superfamily protein	GO:0005506 GO:0016705 GO:0020037 GO:0055114	247.1	128.6	116.0	169.8	303.7	196.7
Ca_12825	-0.0126	0.0097	0.0159	Cytochrome P450 superfamily protein	GO:0005506 GO:0016705 GO:0020037 GO:0055114	2.7	0.0	3.0	4.7	5.0	6.3
Ca_13315	-0.0085	0.0128	0.0154	Cytochrome P450 superfamily protein	GO:0005506 GO:0016705 GO:0020037 GO:0055114	209.3	115.7	194.5	231.2	192.3	295.1
Ca_15198	-0.0116	0.0089	0.0146	Cytochrome P450 superfamily protein	GO:0005506 GO:0016705 GO:0020037 GO:0055114	85.9	136.7	241.6	441.1	187.2	387.7
Ca_22318	-0.0035	0.0141	0.0145	Cytochrome P450 superfamily protein	GO:0005506 GO:0016705 GO:0020037 GO:0055114	162.8	83.8	66.3	103.6	99.7	118.6
Ca_22857	-0.0135	0.0094	0.0164	Cytochrome P450 superfamily protein	GO:0005506 GO:0016705 GO:0020037 GO:0055114	75.4	62.6	263.5	433.2	205.7	580.2
Ca_23399	-0.0103	0.0121	0.0159	Cytochrome P450 superfamily protein	GO:0005506 GO:0016705 GO:0020037 GO:0055114	39.7	41.1	31.8	47.8	75.2	41.9
Ca_26674	-0.0121	0.0054	0.0133	Cytochrome P450 superfamily protein	GO:0005506 GO:0016705 GO:0020037 GO:0055114	82.1	143.5	248.7	283.6	325.4	415.7
Ca_26828	-0.0140	0.0031	0.0144	Cytochrome P450 superfamily protein	GO:0005506 GO:0016705 GO:0020037 GO:0055114	43.4	67.7	91.4	127.0	115.1	162.2
Ca_27703	-0.0148	0.0072	0.0165	Cytochrome P450 superfamily protein	GO:0005506 GO:0016705 GO:0020037 GO:0055114	15.8	24.0	20.3	30.0	44.0	35.7
Ca_25468	-0.0081	0.0123	0.0147	cytomatrix-like protein		297.2	209.7	177.9	285.3	321.4	248.3
Ca_18079	-0.0146	0.0047	0.0154	DCD (Development and Cell Death) domain protein		16.8	13.2	22.5	29.7	29.2	43.8
Ca_07520	-0.0137	0.0107	0.0173	debranching enzyme 1	GO:0003824 GO:0004553 GO:0005975 GO:0043169	292.5	256.5	437.4	587.1	414.1	683.0
Ca_22567	-0.0037	0.0141	0.0145	dehydration-responsive element-binding protein		12.5	0.0	2.5	5.3	5.7	7.5
Ca_23997	-0.0099	0.0141	0.0172	dehydration-responsive protein RD22		43.0	11.4	23.0	35.7	40.8	52.4
Ca_11419	-0.0158	0.0093	0.0183	delta-1-pyrroline-5-carboxylate synthetase	GO:0008152 GO:0016491 GO:0016620 GO:0055114	3.0	3.5	4.4	6.9	7.9	8.7
Ca_18131	-0.0076	0.0134	0.0154	dentin sialophosphoprotein-like isoform X2 [Glycine max]		156.6	140.3	133.4	174.1	163.2	156.3
Ca_04914	-0.0023	0.0150	0.0152	deoxynucleoside triphosphate triphosphohydrolase SAMHD1 homolog isoform X6 [Glycine max]	GO:0008081 GO:0046872	232.2	140.3	124.2	160.3	182.0	162.3
Ca_22595	-0.0094	0.0124	0.0156	Deoxyribodipyrimidine photo-lyase (DNA photolyase)(Photoreactivating enzyme) n=1 Tax=Methanosaeta harundinacea (strain 6Ac) RepID=G7WMK4_METH6	GO:0003904 GO:0003913 GO:0006281	49.6	28.3	22.2	40.1	51.4	57.0
Ca_26646	-0.0007	0.0131	0.0132	Di-glucose binding with Kinesin motor domain-like protein n=1 Tax=Theobroma cacao RepID=UPI00042B2547	GO:0003676 GO:0008270	277.0	167.4	115.9	167.0	169.9	179.3
Ca_28818	-0.0128	0.0025	0.0131	dihydroorotate dehydrogenase (quinone)	GO:0000271 GO:0003824 GO:0004152 GO:0004158 GO:0006207 GO:0006222 GO:0016020	25.5	23.3	31.3	38.7	34.9	38.2
Ca_20656	-0.0018	0.0147	0.0148	disease resistance family protein / LRR family protein	GO:0005515 GO:0006952 GO:0043531	281.4	113.9	109.8	150.6	154.3	163.5
Ca_09490	-0.0102	0.0107	0.0148	disease resistance protein	GO:0006952 GO:0043531	236.2	262.6	172.2	242.6	321.9	314.4
Ca_23289	-0.0082	0.0159	0.0179	disease resistance protein	GO:0006952 GO:0043531	175.6	112.8	104.2	153.7	167.0	144.7
Ca_17592	-0.0138	0.0008	0.0139	disease resistance protein (TIR-NBS-LRR class)		15.2	27.1	33.0	35.1	60.2	50.6
Ca_20190	0.0027	0.0128	0.0131	Disease resistance protein (TIR-NBS-LRR class) family	GO:0005515 GO:0006952 GO:0007165 GO:0043531	938.8	436.6	410.7	433.5	517.0	486.8
Ca_25800	-0.0021	0.0138	0.0140	Disease resistance protein (TIR-NBS-LRR class) family	GO:0005515 GO:0006952 GO:0007165 GO:0043531	2849.0	991.4	776.5	1213.4	1699.0	1463.0
Ca_19136	0.0012	0.0130	0.0130	DNA ligase 1-like isoform X2 [Glycine max]		286.3	34.8	36.9	56.2	59.7	73.3
Ca_13595	-0.0121	0.0111	0.0165	DNA topoisomerase I	GO:0003677 GO:0003916 GO:0003917	67.8	16.2	5.7	7.7	593.1	438.6
Ca_27017	-0.0118	0.0099	0.0154	DNA/RNA polymerases superfamily protein n=1 Tax=Theobroma cacao RepID=UPI00042B3946		10.4	10.5	4.8	10.4	14.8	14.6
Ca_23174	-0.0050	0.0137	0.0146	DNA-binding storekeeper protein-related transcriptional regulator		93.2	30.9	22.9	46.5	76.6	48.1

Ca_28521	-0.0096	0.0138	0.0168	DNA-binding transcriptional repressor of ribose metabolism n=1 Tax=Photobacterium phosphoreum ANT220 RepID=W9A6N4_PHOPO	GO:0003700	11.4	5.9	6.5	18.7	8.2	11.7
Ca_26486	-0.0144	0.0071	0.0160	DNA-directed RNA polymerase family protein	GO:0003677 GO:0003899 GO:0006351 GO:0032549	4.4	4.0	5.8	8.2	8.2	11.5
Ca_18311	-0.0131	0.0020	0.0133	DNAJ heat shock family protein	GO:0006457 GO:0031072 GO:0051082	14.1	27.5	21.1	50.0	41.0	36.8
Ca_02320	-0.0079	0.0143	0.0164	dnaJ homolog subfamily B member 1-like isoform 1 [Glycine max]		218.4	222.3	186.2	215.6	245.8	268.6
Ca_25449	-0.0139	0.0028	0.0141	Domain of unknown function (DUF313)		10.5	13.8	18.2	21.3	24.8	28.2
Ca_19415	-0.0122	0.0093	0.0153	Dormancy/auxin associated family protein		0.0	0.0	0.0	6.0	4.2	0.0
Ca_13472	-0.0133	0.0064	0.0147	DUF4408 domain protein		19.9	40.0	18.4	35.5	47.4	45.5
Ca_05529	-0.0147	0.0063	0.0160	DUF674 family protein		24.6	37.4	27.7	40.6	42.9	53.8
Ca_26136	-0.0155	0.0078	0.0174	Dynein light chain type 1 family protein	GO:0005875 GO:0007017	0.0	3.4	0.0	4.6	9.1	11.3
Ca_19622	-0.0134	0.0102	0.0168	E3 ubiquitin-protein ligase RGLG2-like isoform X3 [Glycine max]	GO:0005515 GO:0008270	67.4	70.8	65.1	132.5	98.5	115.2
Ca_10038	-0.0142	0.0060	0.0154	enhancer of polycomb-like transcription factor protein	GO:0032777 GO:0035267	142.3	131.7	129.7	174.7	230.7	210.3
Ca_03363	-0.0021	0.0132	0.0134	ERD (early-responsive to dehydration stress) family protein	GO:0016020	583.0	379.0	452.6	520.5	465.5	460.7
Ca_13780	-0.0139	0.0045	0.0146	Ethylene insensitive 3 family protein		22.5	17.8	25.9	39.3	35.6	50.6
Ca_09294	-0.0112	0.0084	0.0140	ethylene responsive element binding factor 5	GO:0003677 GO:0003700 GO:0006355	1.5	0.0	1.0	2.6	14.6	6.0
Ca_04328	-0.0118	0.0067	0.0136	ethylene-responsive transcription factor 12 [Glycine max]	GO:0003677 GO:0003700 GO:0006355	34.3	43.4	28.9	71.1	44.0	65.9
Ca_22742	-0.0035	0.0136	0.0140	ethylene-responsive transcription factor 1-like [Glycine max]	GO:0003677 GO:0003700 GO:0006355	22.9	10.6	14.7	16.4	15.2	19.8
Ca_12887	-0.0132	0.0039	0.0137	ethylene-responsive transcription factor 7-like [Glycine max]	GO:0003677 GO:0003700 GO:0006355	60.9	64.5	60.4	92.8	146.4	113.2
Ca_20027	-0.0138	0.0134	0.0192	ethylene-responsive transcription factor 7-like [Glycine max]	GO:0003677 GO:0003700 GO:0006355	138.5	112.6	87.7	1070.6	1005.2	149.4
Ca_08782	-0.0116	0.0071	0.0136	Eukaryotic aspartyl protease family protein	GO:0004190 GO:0006508	0.0	0.0	0.0	0.0	4.6	2.8
Ca_19963	0.0016	0.0130	0.0131	Eukaryotic aspartyl protease family protein	GO:0004190 GO:0006508	574.9	233.3	221.6	290.9	332.5	249.7
Ca_20521	-0.0102	0.0082	0.0131	evolutionarily conserved C-terminal region 2		5.4	11.2	0.0	14.3	13.5	8.0
Ca_18472	-0.0112	0.0081	0.0138	exocyst subunit exo70 family protein H7	GO:0000145 GO:0006887	512.3	252.2	436.6	599.8	706.7	830.3
Ca_15048	-0.0063	0.0125	0.0140	FASCICLIN-like arabinogalactan 2		29.9	24.0	12.9	23.3	34.6	34.0
Ca_26489	-0.0139	0.0070	0.0155	FBD-associated F-box plant protein		0.0	4.5	0.0	5.0	9.4	7.0
Ca_11096	-0.0045	0.0129	0.0136	F-box family protein	GO:0005515	109.9	48.9	80.1	116.1	66.5	87.0
Ca_29383	-0.0130	0.0072	0.0149	F-box family protein		4.4	1.8	7.3	26.4	61.3	18.8
Ca_20485	-0.0058	0.0120	0.0133	F-box protein	GO:0005515	52.4	15.9	19.8	25.9	43.8	38.0
Ca_30184	-0.0133	0.0040	0.0139	F-box/LRR protein		23.8	25.2	26.6	35.3	40.6	49.6
Ca_29433	-0.0137	0.0047	0.0145	F-box/LRR protein		6.3	7.8	9.3	22.9	46.0	28.6
Ca_16566	0.0002	0.0131	0.0131	F-box/RNI-like superfamily protein	GO:0005515	314.0	147.3	131.9	139.5	193.2	149.7
Ca_23717	-0.0143	0.0086	0.0166	F-box/RNI-like superfamily protein	GO:0005515	5.4	6.5	4.0	8.8	7.1	11.5
Ca_05348	-0.0182	0.0108	0.0212	FKBP-like peptidyl-prolyl cis-trans isomerase family protein	GO:0006457	0.0	0.0	0.0	5.9	6.3	7.9
Ca_28325	-0.0161	0.0070	0.0176	Flagellar motor protein MotA n=3 Tax=Alcaligenes RepID=J0B666_ALCFA	GO:0001539 GO:0003677 GO:0003774 GO:0006810 GO:0006935 GO:0008565 GO:0016020 GO:0044780 GO:0045893 GO:1902208	1.4	4.3	1.9	8.1	8.7	13.7
Ca_26879	-0.0087	0.0114	0.0144	flap endonuclease GEN-like protein	GO:0003677 GO:0003824 GO:0004518 GO:0006281	64.4	54.1	54.6	65.9	72.1	85.9
Ca_23809	-0.0126	0.0058	0.0139	Flavin oxidoreductase / NADH oxidase family protein n=146 Tax=Pseudomonas RepID=T2E7A4_PSEAI	GO:0003824 GO:0010181 GO:0016491 GO:0055114	11.1	16.9	7.2	20.5	22.9	17.0
Ca_13912	-0.0102	0.0097	0.0141	Flavin-containing monooxygenase family protein	GO:0016491 GO:0055114	48.8	33.8	33.2	50.6	62.9	53.6
Ca_26889	-0.0116	0.0089	0.0146	flocculation protein FLO11-like isoform X4 [Glycine max]		0.0	0.0	0.0	9.6	0.0	3.9
Ca_04977	-0.0134	0.0093	0.0163	folylpolyglutamate synthase-like isoform X2 [Glycine max]	GO:0004326 GO:0005524 GO:0009058 GO:0009396	0.3	0.5	3.4	16.4	4.9	6.4
Ca_01574	-0.0136	0.0067	0.0152	Gag polyprotein n=1 Tax=Cicer arietinum RepID=Q949L5_CICAR		2.9	4.4	0.0	9.2	8.8	7.4
Ca_18681	-0.0131	0.0073	0.0150	Gag polyprotein n=1 Tax=Cicer arietinum RepID=Q949L5_CICAR		3.6	0.0	0.0	2.8	8.2	9.8
Ca_24682	-0.0131	0.0060	0.0144	Gag polyprotein n=1 Tax=Cicer arietinum RepID=Q949L5_CICAR		2.7	0.0	3.6	4.8	16.2	11.1
Ca_24955	-0.0150	0.0096	0.0178	Gag polyprotein n=1 Tax=Cicer arietinum RepID=Q949L5_CICAR		0.0	0.0	0.0	3.4	4.0	4.5
Ca_26096	-0.0114	0.0071	0.0134	Gag polyprotein n=1 Tax=Cicer arietinum RepID=Q949L5_CICAR		0.0	0.0	0.0	3.5	0.0	5.4
Ca_28033	-0.0185	0.0127	0.0224	Gag polyprotein n=1 Tax=Cicer arietinum RepID=Q949L5_CICAR		0.0	0.0	0.0	3.3	3.9	4.7
Ca_28025	-0.0119	0.0056	0.0131	Gag-protease polyprotein-like protein n=1 Tax=Cicer arietinum RepID=Q8H6W2_CICAR	GO:0003676 GO:0008270	0.0	0.8	0.0	0.0	2.5	3.1
Ca_08157	-0.0119	0.0078	0.0143	galactinol synthase 1	GO:0016757	202.0	171.4	242.3	277.0	343.3	310.1
Ca_00106	0.0000	0.0131	0.0131	galactoside 2-alpha-L-fucosyltransferase-like protein	GO:0008107 GO:0016020 GO:0042546	244.7	117.2	105.0	112.5	143.1	138.8
Ca_15771	-0.0089	0.0157	0.0180	GATA type zinc finger transcription factor family protein	GO:0008270	68.1	39.3	30.2	48.7	68.6	77.4
Ca_24989	-0.0086	0.0130	0.0156	GDSL-like Lipase/Acylhydrolase superfamily protein	GO:0006629 GO:0016787 GO:0016788	169.2	123.7	195.1	241.5	217.0	176.9
Ca_02934	-0.0041	0.0129	0.0135	germin-like protein 1	GO:0030145 GO:0045735	159.6	78.0	63.2	73.9	156.9	117.7
Ca_28384	-0.0137	0.0085	0.0162	gluconate 2-dehydrogenase n=1 Tax=Pantoea sp. A4 RepID=UPI00037CC94E		3.6	0.0	0.0	10.3	4.7	10.8
Ca_28642	-0.0152	0.0096	0.0179	glutamate synthase 2	GO:0008152 GO:0016491 GO:0055114	1.4	0.0	0.0	4.7	2.0	5.9

Ca_20445	-0.0198	0.0114	0.0229	glutathione S-transferase F3	GO:0003824 GO:0005515 GO:0005737 GO:0009072	11.5	23.4	20.3	268.0	257.2	244.9
Ca_29236	-0.0129	0.0033	0.0133	glutathione S-transferase F3	GO:0005515	0.0	0.0	4.0	6.4	3.4	7.8
Ca_06711	-0.0116	0.0093	0.0149	Glycerophosphodiester phosphodiesterase GDE1 n=2 Tax=Triticeae RepID=M8BLH1_AEGTA	GO:0006071 GO:0006629 GO:0008081 GO:0008889	4987.1	2057.6	5471.2	5453.2	9171.3	8517.0
Ca_11839	-0.0085	0.0121	0.0148	Glycosyl hydrolase family 10 protein	GO:0004553 GO:0005975 GO:0016798	40.5	25.6	27.2	37.6	56.5	34.2
Ca_25354	-0.0133	0.0092	0.0162	Got1/Sft2-like vescicle transport protein family		1.9	0.0	0.0	5.7	20.3	8.5
Ca_11898	-0.0113	0.0065	0.0131	GTP-binding nuclear protein Ran-3 [Glycine max]	GO:0003924 GO:0005525 GO:0005622 GO:0006184 GO:0006886 GO:0006913 GO:0007165	217.9	310.8	345.2	797.8	425.6	351.5
Ca_13764	-0.0012	0.0142	0.0143	GTP-binding nuclear protein Ran-3 [Glycine max]	GO:0005525 GO:0005622 GO:0006184 GO:0007165 GO:0007264 GO:0015031 GO:0016020	215.9	101.3	89.7	121.7	130.3	123.3
Ca_15971	-0.0013	0.0137	0.0137	GTP-binding nuclear protein Ran-3 [Glycine max]	GO:0005525 GO:0005622 GO:0006184 GO:0007165 GO:0007264 GO:0015031 GO:0016020	112.5	41.8	60.1	64.6	88.7	53.3
Ca_25349	-0.0061	0.0156	0.0167	haloacid dehalogenase-like hydrolase	GO:0008152 GO:0016787	220.8	85.0	78.6	129.4	211.9	155.3
Ca_05160	-0.0145	0.0069	0.0161	haloacid dehalogenase-like hydrolase domain protein		0.0	0.0	0.0	1.8	7.5	5.8
Ca_13528	-0.0066	0.0130	0.0146	HEAT repeat-containing protein 6-like isoform X1 [Glycine max]	GO:0005488	292.5	204.2	189.8	250.8	281.8	244.8
Ca_28932	-0.0119	0.0051	0.0130	Heat shock protein A n=3 Tax=Enterobacteriaceae RepID=Q2NX42_SODGM		11.9	16.4	12.4	23.5	31.1	35.4
Ca_02960	-0.0041	0.0160	0.0165	Heavy metal transport/detoxification superfamily protein	GO:0030001 GO:0046872	253.8	96.2	78.9	162.0	160.8	122.0
Ca_24603	-0.0125	0.0064	0.0141	Heavy metal transport/detoxification superfamily protein	GO:0030001 GO:0046872	6.9	15.5	10.7	36.0	15.9	20.5
Ca_19977	-0.0131	0.0005	0.0131	heme-binding protein 2 [Glycine max]		25.9	21.1	32.5	44.3	61.5	49.9
Ca_05293	0.0007	0.0137	0.0138	histone-lysine N-methyltransferase	GO:0005634 GO:0008270 GO:0018024 GO:0034968	161.1	66.7	48.4	66.0	75.8	69.6
Ca_26411	-0.0130	0.0036	0.0135	histone-lysine N-methyltransferase	GO:0005515 GO:0005634 GO:0008270 GO:0018024 GO:0034968 GO:0042393	0.0	2.2	1.5	3.6	5.2	5.3
Ca_26617	-0.0118	0.0064	0.0135	histone-lysine N-methyltransferase ATXR6-like isoform X1 [Glycine max]	GO:0005515 GO:0008270	0.8	2.7	0.0	4.0	4.0	8.3
Ca_28113	-0.0143	0.0096	0.0172	homeobox-leucine zipper protein ANTHOCYANINLESS 2-like isoform X6 [Glycine max]	GO:0008289	14.0	10.0	8.3	19.2	19.3	19.3
Ca_03079	-0.0093	0.0091	0.0130	HXXXD-type acyl-transferase family protein	GO:0016747	235.2	166.2	337.7	373.0	271.1	363.8
Ca_29434	-0.0132	0.0070	0.0149	HXXXD-type acyl-transferase family protein	GO:0016747	2.1	0.0	0.0	6.8	2.2	6.5
Ca_00901	-0.0144	0.0131	0.0195	hypothetical protein		40.5	34.6	32.0	53.4	49.2	66.7
Ca_22347	-0.0099	0.0103	0.0143	hypothetical protein		19.8	16.0	11.0	24.4	17.5	24.9
Ca_23670	-0.0157	0.0123	0.0199	hypothetical protein		0.0	1.8	0.0	6.1	7.2	5.0
Ca_25405	-0.0124	0.0066	0.0141	hypothetical protein	GO:0003677	36.5	29.6	41.8	82.2	51.3	48.7
Ca_00016	-0.0138	0.0054	0.0148	hypothetical protein		0.0	0.0	1.8	2.0	3.6	7.8
Ca_01557	-0.0123	0.0063	0.0138	hypothetical protein		0.0	4.2	0.0	5.7	4.6	7.9
Ca_03619	-0.0125	0.0068	0.0142	hypothetical protein		8.3	9.8	7.1	16.1	19.3	15.7
Ca_04290	-0.0066	0.0150	0.0164	hypothetical protein		97.3	68.5	68.4	98.0	77.9	102.7
Ca_04627	-0.0122	0.0098	0.0156	hypothetical protein		1.7	0.0	0.0	13.2	4.7	4.6
Ca_05127	-0.0190	0.0131	0.0231	hypothetical protein		0.0	0.0	0.0	10.8	9.0	6.1
Ca_05600	-0.0060	0.0119	0.0133	hypothetical protein		4.8	0.0	0.0	3.6	6.6	0.0
Ca_05962	-0.0166	0.0100	0.0194	hypothetical protein		3.2	0.0	0.0	5.1	9.5	10.2
Ca_11131	-0.0113	0.0096	0.0148	hypothetical protein		20.6	14.1	6.7	19.2	29.5	62.5
Ca_11148	-0.0096	0.0104	0.0142	hypothetical protein		831.4	512.7	303.3	786.8	632.6	1603.0
Ca_11189	-0.0119	0.0074	0.0140	hypothetical protein		2.9	0.0	0.0	6.6	3.3	6.0
Ca_11501	-0.0125	0.0062	0.0139	hypothetical protein		15.4	18.0	14.5	21.8	34.4	28.2
Ca_12280	-0.0120	0.0064	0.0136	hypothetical protein		0.0	0.0	0.0	0.0	6.9	6.6
Ca_12467	-0.0128	0.0039	0.0134	hypothetical protein		1.6	7.5	0.0	8.1	8.5	15.9
Ca_18621	-0.0138	0.0079	0.0159	hypothetical protein		11.5	16.1	10.8	19.5	25.6	25.9
Ca_19596	-0.0152	0.0055	0.0162	hypothetical protein		5.5	3.6	6.9	13.5	12.1	13.3
Ca_19597	-0.0147	0.0102	0.0179	hypothetical protein		1.4	2.6	0.0	5.7	4.2	3.8
Ca_19627	-0.0172	0.0113	0.0206	hypothetical protein		0.0	0.0	0.0	6.9	7.4	6.0
Ca_19674	-0.0128	0.0046	0.0136	hypothetical protein		4.2	8.6	6.1	12.8	20.9	16.6
Ca_20988	-0.0139	0.0014	0.0139	hypothetical protein		5.5	14.0	23.2	28.8	39.5	25.7
Ca_21019	0.0015	0.0136	0.0137	hypothetical protein		96.8	21.9	10.6	19.8	24.9	21.6
Ca_23763	-0.0128	0.0081	0.0151	hypothetical protein		2.9	6.6	9.7	11.1	28.3	53.2
Ca_23933	-0.0117	0.0066	0.0134	hypothetical protein		3.2	6.1	3.3	7.2	10.2	12.8
Ca_24462	-0.0114	0.0077	0.0138	hypothetical protein		7.3	12.7	7.5	8.9	23.6	21.5
Ca_24772	-0.0152	0.0092	0.0177	hypothetical protein		1.4	0.0	2.8	5.3	4.2	7.4
Ca_25273	-0.0122	0.0049	0.0131	hypothetical protein		4.8	7.7	6.3	18.3	11.4	19.2
Ca_25408	-0.0140	0.0050	0.0148	hypothetical protein		4.9	5.3	7.9	10.5	16.2	19.2
Ca_25470	-0.0168	0.0094	0.0192	hypothetical protein		0.0	0.0	0.0	3.4	14.0	8.5
Ca_26005	-0.0153	0.0054	0.0162	hypothetical protein		6.5	12.6	10.6	15.4	15.8	21.4
Ca_26637	-0.0123	0.0056	0.0135	hypothetical protein		6.9	10.4	6.4	15.1	11.2	19.3
Ca_27029	-0.0136	0.0099	0.0168	hypothetical protein		1.1	1.3	3.1	11.1	24.9	13.6
Ca_27291	-0.0128	0.0037	0.0133	hypothetical protein		5.9	7.5	8.3	7.2	19.7	18.1
Ca_27729	-0.0050	0.0142	0.0151	hypothetical protein		383.4	146.5	222.1	338.0	279.8	242.6
Ca_27806	-0.0118	0.0100	0.0155	hypothetical protein		0.0	0.0	0.0	6.8	3.0	0.0
Ca_27856	-0.0114	0.0098	0.0150	hypothetical protein		0.0	1.2	0.0	8.9	0.0	4.3
Ca_28116	-0.0165	0.0098	0.0192	hypothetical protein		2.4	0.5	0.0	4.9	6.6	7.0
Ca_28125	-0.0126	0.0056	0.0138	hypothetical protein		1.8	3.7	3.1	3.4	14.0	9.0
Ca_28234	-0.0051	0.0121	0.0131	hypothetical protein		2.4	0.0	0.0	1.9	2.9	0.0
Ca_29378	-0.0153	0.0072	0.0169	hypothetical protein		2.7	0.0	6.6	19.5	15.3	12.9
Ca_29437	-0.0115	0.0072	0.0135	hypothetical protein		0.0	6.9	0.0	7.0	4.9	8.0
Ca_03395	-0.0121	0.0072	0.0141	inositol hexakisphosphate and diphosphoinositol-pentakisphosphate kinase-like isoform X1 [Glycine max]		0.0	2.4	0.0	4.0	5.5	9.7
Ca_28700	-0.0133	0.0056	0.0144	Inositol monophosphatase family protein	GO:0046854	5.2	4.6	4.1	11.8	9.0	8.8

Ca_19081	-0.0154	0.0061	0.0166	Integral membrane protein n=1 Tax=Beta vulgaris RepID=Q39416_BETVU	GO:0016020 GO:0016021 GO:0022857 GO:0022891 GO:0055085	12.1	11.6	7.2	19.2	28.9	32.6
Ca_29145	-0.0117	0.0060	0.0132	integral membrane TerC family protein	GO:0016021	3.3	4.9	0.0	10.9	4.7	8.9
Ca_09530	-0.0124	0.0128	0.0178	K+ efflux antiporter 6	GO:0006812 GO:0015299 GO:0016021 GO:0055085	4.3	0.0	0.0	5.7	6.0	4.5
Ca_28727	-0.0131	0.0026	0.0133	ketol-acid reductoisomerase	GO:0004455 GO:0008652 GO:0009082 GO:0016491 GO:0016616 GO:0050661 GO:0050662	6.9	7.7	10.5	16.5	14.3	15.7
Ca_13183	-0.0139	0.0018	0.0140	Kinase interacting (KIP1-like) family protein		79.2	101.6	90.4	130.9	150.4	194.9
Ca_05539	-0.0127	0.0108	0.0167	laccase 17	GO:0005507 GO:0016491 GO:0055114	335.7	215.0	208.3	293.4	389.9	423.4
Ca_24884	-0.0004	0.0141	0.0142	laccase 7	GO:0005507 GO:0016491 GO:0055114	272.0	82.3	83.0	120.3	133.0	91.9
Ca_18000	-0.0139	0.0086	0.0164	Lactoylglutathione lyase / glyoxalase I family protein		0.0	1.8	0.0	3.9	3.3	6.4
Ca_18092	-0.0155	0.0076	0.0173	L-ascorbate oxidase homolog [Glycine max]	GO:0005507 GO:0016491 GO:0055114	10.0	14.3	9.6	31.0	31.5	24.0
Ca_14840	-0.0133	0.0011	0.0133	Lecithin:cholesterol acyltransferase family protein	GO:0006629 GO:0008374	54.2	54.7	48.8	82.9	107.7	104.9
Ca_16540	-0.0136	0.0103	0.0170	leguminosin group485 secreted peptide		4.7	2.6	0.0	9.0	15.6	8.3
Ca_23996	-0.0136	0.0057	0.0147	leguminosin group486 secreted peptide		6.8	3.9	8.4	14.6	55.8	16.4
Ca_29160	-0.0139	0.0047	0.0147	Lipid A biosynthesis lauroyl (Or palmitoleoyl) acyltransferase n=4 Tax=Enterobacteriaceae RepID=E6WBT8_PANSA	GO:0009244 GO:0009276 GO:0016021 GO:0016746 GO:0016747	6.7	13.9	10.4	16.2	22.2	16.9
Ca_27784	-0.0132	0.0042	0.0139	LMBR1-like membrane protein		4.5	2.6	2.8	7.3	6.6	8.6
Ca_27376	-0.0143	0.0066	0.0157	long-chain acyl-CoA synthetase 2	GO:0003824 GO:0008152	0.0	0.0	4.1	5.7	4.6	9.0
Ca_16282	-0.0050	0.0140	0.0149	long-chain acyl-CoA synthetase 7	GO:0003824 GO:0008152	885.7	215.0	204.8	241.1	500.5	601.1
Ca_17466	-0.0078	0.0133	0.0154	Low PSII Accumulation 3		125.0	15.9	64.0	100.7	60.1	99.2
Ca_11948	-0.0090	0.0145	0.0171	LRR and NB-ARC domain disease resistance protein	GO:0006952 GO:0043531	212.3	160.0	140.1	181.8	240.0	195.5
Ca_26750	-0.0063	0.0126	0.0141	LRR and NB-ARC domain disease resistance protein		399.7	228.5	218.1	301.5	289.8	457.8
Ca_28761	-0.0162	0.0072	0.0177	lysine-tRNA ligase-like protein	GO:0000166 GO:0004812 GO:0004824 GO:0005524 GO:0005737 GO:0006418 GO:0006430	1.2	0.0	5.0	10.4	6.5	12.8
Ca_03633	-0.0144	0.0045	0.0151	MADS-box transcription factor	GO:0003700 GO:0005634 GO:0006355	11.2	9.6	7.4	19.0	21.3	30.0
Ca_10553	-0.0160	0.0063	0.0172	MADS-box transcription factor family protein	GO:0003677 GO:0046983	4.7	7.6	6.2	9.3	14.3	17.4
Ca_27151	-0.0136	0.0034	0.0140	MADS-box transcription factor family protein		3.1	5.9	4.0	10.7	8.2	10.9
Ca_02990	-0.0120	0.0090	0.0150	Magnesium transporter CorA-like family protein	GO:0016020 GO:0030001 GO:0046873 GO:0055085	1.9	0.0	0.0	3.4	1.3	5.0
Ca_10599	-0.0078	0.0117	0.0140	Major facilitator superfamily protein	GO:0016021 GO:0055085	158.7	131.5	105.3	119.8	184.4	187.8
Ca_28343	-0.0151	0.0104	0.0183	Major facilitator superfamily protein	GO:0016021 GO:0055085	1.8	6.5	3.7	24.6	18.5	11.3
Ca_04585	-0.0060	0.0150	0.0162	MATE efflux family protein	GO:0006855 GO:0015238 GO:0015297 GO:0016020 GO:0055085	84.6	80.0	52.1	73.9	88.2	83.1
Ca_22890	-0.0114	0.0084	0.0142	MATE efflux family protein	GO:0006855 GO:0015238 GO:0015297 GO:0016020 GO:0055085	223.5	211.0	226.7	231.6	295.6	366.6
Ca_10890	-0.0029	0.0130	0.0133	mediator of RNA polymerase II transcription subunit 23		166.1	121.8	98.8	127.1	139.0	144.4
Ca_20599	-0.0164	0.0065	0.0177	mediator of RNA polymerase II transcription subunit 4-like isoform X4 [Glycine max]	GO:0001104 GO:0006357 GO:0016592	406.2	453.2	513.0	1717.9	1017.3	1156.0
Ca_16115	-0.0157	0.0065	0.0170	Membrane transporter D1 n=3 Tax=Andropogoneae RepID=B6U4Q3_MAIZE	GO:0005215 GO:0006810 GO:0016020 GO:0016021 GO:0022857 GO:0022891 GO:0055085	63.2	87.8	47.3	112.4	166.4	163.0
Ca_09511	-0.0110	0.0096	0.0146	Metal transport protein n=1 Tax=Medicago truncatula RepID=Q6VM17_MEDTR	GO:0016020 GO:0030001 GO:0046873 GO:0055085	0.0	0.0	0.0	3.7	3.3	0.5
Ca_08865	-0.0045	0.0134	0.0142	metal-nicotianamine transporter YSL1-like isoform X2 [Glycine max]	GO:0055085	1014.2	496.7	541.9	855.4	695.1	672.7
Ca_28903	-0.0124	0.0109	0.0165	methionyl-tRNA formyltransferase		4.8	6.6	0.0	17.2	7.6	9.1
Ca_20765	-0.0054	0.0160	0.0169	methyl esterase 1		172.9	3.1	0.0	40.4	40.5	42.7
Ca_20480	-0.0141	0.0027	0.0144	Mitochondrial transcription termination factor family protein		8.5	11.6	17.0	17.0	29.4	27.8
Ca_25777	-0.0116	0.0116	0.0164	molybdenum cofactor biosynthesis protein A	GO:0003824 GO:0006777 GO:0019008 GO:0046872 GO:0051536 GO:0051539	57.0	60.0	48.8	72.1	73.4	80.5
Ca_14800	-0.0025	0.0136	0.0138	molybdenum cofactor sulfurase-like [Glycine max]	GO:0003824 GO:0030170	569.3	314.1	287.8	347.9	420.1	438.8
Ca_29218	-0.0153	0.0113	0.0190	Molybdopterin guanine dinucleotide-containing S/N-oxide reductase n=17 Tax=Serratia RepID=A8GGU1_SERP5	GO:0016491 GO:0030151 GO:0055114	3.6	2.6	1.6	7.6	6.7	8.6
Ca_28920	-0.0126	0.0071	0.0144	Molybdopterin-guanine dinucleotide biosynthesis protein B n=3 Tax=Enterobacter RepID=V3I9J0_ENTCL	GO:0003824 GO:0005525 GO:0006777	2.1	0.0	5.4	13.8	4.7	7.0
Ca_28398	-0.0154	0.0113	0.0190	Multidrug transporter n=4 Tax=Enterobacteriaceae RepID=W6JAR3_9ENTR	GO:0006855 GO:0015238 GO:0015297 GO:0016020 GO:0055085	2.2	2.6	0.0	11.0	5.1	7.4
Ca_06446	-0.0075	0.0107	0.0131	myb transcription factor	GO:0003677 GO:0003682	82.9	68.3	68.6	82.7	75.8	98.2
Ca_15359	-0.0089	0.0119	0.0149	myb transcription factor	GO:0003677 GO:0003682	180.5	106.3	139.5	174.4	216.7	204.3
Ca_21006	-0.0139	0.0017	0.0140	myb transcription factor	GO:0003677 GO:0003682	25.9	58.5	39.8	71.1	118.7	101.7
Ca_23386	-0.0151	0.0116	0.0190	MYB transcription factor MYB51 [Glycine max]	GO:0003677	7.7	8.6	4.0	13.5	14.7	13.4
Ca_25372	-0.0134	0.0064	0.0148	myosin 2	GO:0005515	12.4	8.3	9.7	21.3	29.2	21.9
Ca_20646	-0.0033	0.0129	0.0133	myosin-2 heavy chain-like [Glycine max]		161.5	132.4	91.4	149.1	107.5	140.8
Ca_25369	-0.0131	0.0076	0.0151	n=1 Tax=Oryza sativa subsp. japonica RepID=Q7XU62_ORYSJ	GO:0008270	0.0	0.0	0.0	0.0	2.3	3.5
Ca_00935	-0.0152	0.0061	0.0164	NAC domain protein	GO:0003677 GO:0006355	15.2	15.1	18.4	40.5	40.7	34.1
Ca_20365	-0.0102	0.0098	0.0141	NAD(P)-binding Rossmann-fold superfamily protein	GO:0008152 GO:0016491	38.4	40.4	39.3	39.6	51.0	68.3
Ca_20366	-0.0036	0.0135	0.0140	NAD(P)-binding Rossmann-fold superfamily protein	GO:0008152 GO:0016491	86.4	18.5	25.0	37.4	42.3	44.6

Ca_26948	-0.0138	0.0014	0.0139	NAD(P)-binding Rossmann-fold superfamily protein	GO:0003682 GO:0008152 GO:0016491	10.4	20.7	24.0	25.7	46.2	49.6
Ca_12977	-0.0115	0.0063	0.0131	ninja-family protein AFP2-like [Glycine max]		3.5	11.0	4.2	13.7	10.4	11.1
Ca_17328	-0.0142	0.0050	0.0150	nucleoporin NUP188 homolog isoform X1 [Glycine max]		7.0	8.0	7.7	12.8	13.5	14.6
Ca_11821	-0.0130	0.0048	0.0139	Nucleotide-sugar transporter/ sugar porter n=3 Tax=Zea mays RepID=B6UBN6_MAIZE	GO:0000139 GO:0005338 GO:0005351 GO:0008643 GO:0015780 GO:0016021	0.0	0.0	5.4	8.7	3.2	7.3
Ca_28861	-0.0123	0.0063	0.0138	NUDIX hydrolase n=6 Tax=Enterobacteriaceae RepID=E0LZ24_9ENTR	GO:0016787	0.0	0.0	0.0	31.7	11.4	7.4
Ca_24494	-0.0071	0.0128	0.0147	O-methyltransferase family protein	GO:0008168 GO:0008171 GO:0046983	349.0	191.2	296.9	403.4	276.3	346.2
Ca_28528	-0.0116	0.0082	0.0142	Outer membrane transport energization protein ExbB n=2 Tax=Serratia RepID=L0MN77_SERMA	GO:0005215 GO:0006810 GO:0008565 GO:0016020	4.5	2.5	0.0	7.6	44.0	11.5
Ca_28842	-0.0131	0.0080	0.0153	Oxidoreductase domain protein n=5 Tax=Pantoea RepID=E0M2L9_9ENTR	GO:0016491	0.0	0.0	0.0	3.2	0.0	2.4
Ca_30064	-0.0115	0.0084	0.0143	pantothenate kinase 2-like isoform X1 [Glycine max]		0.0	0.0	0.0	22.6	0.0	5.9
Ca_10183	-0.0143	0.0075	0.0161	Para-aminonezoic acid synthase n=2 Tax=Streptomyces RepID=M3E6S7_9ACTO	GO:0009058 GO:0009396 GO:0016833	5.0	7.8	9.8	21.6	18.1	19.0
Ca_15778	-0.0157	0.0092	0.0182	Pathogenesis-related thaumatin superfamily protein		4.8	14.0	7.0	19.6	85.3	101.2
Ca_18039	-0.0147	0.0021	0.0148	PEBP (phosphatidylethanolamine-binding protein) family protein		3.1	7.3	15.7	19.0	17.8	23.0
Ca_12162	-0.0095	0.0135	0.0165	pectinesterase family protein	GO:0005618 GO:0030599 GO:0042545	13.3	11.0	7.5	13.1	15.7	14.9
Ca_25688	-0.0112	0.0099	0.0149	pectinesterase family protein	GO:0005618 GO:0030599 GO:0042545	2.1	1.8	0.0	4.1	3.1	6.3
Ca_01895	-0.0094	0.0138	0.0167	pectinesterase/pectinesterase inhibitor-like [Glycine max]		1370.6	1100.8	1367.7	1639.5	1981.0	1864.2
Ca_02181	-0.0140	0.0070	0.0157	pentatricopeptide (PPR) repeat-containing protein	GO:0005515	10.7	10.7	5.5	17.6	15.9	20.3
Ca_00071	-0.0179	0.0104	0.0207	Pentatricopeptide repeat (PPR) superfamily protein	GO:0005515	0.0	0.0	0.0	7.0	8.7	9.4
Ca_01376	-0.0040	0.0144	0.0149	Pentatricopeptide repeat (PPR) superfamily protein	GO:0005515	123.5	86.3	55.3	77.2	111.3	94.6
Ca_01585	-0.0045	0.0130	0.0138	Pentatricopeptide repeat (PPR) superfamily protein	GO:0005515	240.5	205.6	146.7	198.2	234.4	207.0
Ca_02449	-0.0151	0.0075	0.0169	Pentatricopeptide repeat (PPR) superfamily protein	GO:0005515	103.7	91.6	107.6	174.2	142.3	150.3
Ca_05095	-0.0104	0.0128	0.0165	Pentatricopeptide repeat (PPR) superfamily protein	GO:0005515	68.1	63.4	45.1	71.3	81.7	77.5
Ca_05286	-0.0020	0.0137	0.0138	Pentatricopeptide repeat (PPR) superfamily protein		63.4	29.6	16.6	28.6	36.9	36.2
Ca_06674	-0.0134	0.0095	0.0164	Pentatricopeptide repeat (PPR) superfamily protein	GO:0005515	209.2	210.5	211.0	283.2	330.5	378.0
Ca_08561	-0.0072	0.0113	0.0134	Pentatricopeptide repeat (PPR) superfamily protein	GO:0005515	97.6	51.6	41.6	72.5	69.6	100.7
Ca_10709	-0.0105	0.0079	0.0132	Pentatricopeptide repeat (PPR) superfamily protein	GO:0005515	154.4	170.8	122.1	149.3	219.2	225.2
Ca_18203	-0.0153	0.0051	0.0161	Pentatricopeptide repeat (PPR) superfamily protein	GO:0005515	698.4	519.9	1277.3	1421.2	7000.1	8049.6
Ca_21252	-0.0127	0.0046	0.0135	Pentatricopeptide repeat (PPR) superfamily protein		0.7	0.0	8.1	11.6	6.6	8.2
Ca_22340	-0.0069	0.0111	0.0131	Pentatricopeptide repeat (PPR) superfamily protein		101.3	74.4	67.3	74.5	134.0	102.8
Ca_22627	-0.0045	0.0145	0.0152	Pentatricopeptide repeat (PPR) superfamily protein	GO:0005515	217.6	88.4	58.2	124.5	146.4	107.4
Ca_23901	-0.0126	0.0077	0.0148	Pentatricopeptide repeat (PPR) superfamily protein	GO:0005515	2.4	3.7	1.8	4.0	14.2	14.4
Ca_27739	-0.0117	0.0068	0.0135	Pentatricopeptide repeat (PPR) superfamily protein		25.7	31.7	13.4	31.3	38.0	60.3
Ca_29577	-0.0121	0.0070	0.0140	Pentatricopeptide repeat (PPR) superfamily protein	GO:0005515	1.1	0.0	0.0	3.8	12.3	7.6
Ca_27543	-0.0119	0.0102	0.0156	Pentatricopeptide repeat (PPR) superfamily protein		0.0	0.0	0.0	10.1	7.8	0.0
Ca_01614	-0.0028	0.0128	0.0131	Pentatricopeptide repeat (PPR-like) superfamily protein	GO:0005515	295.8	155.7	182.9	255.1	218.7	185.9
Ca_11875	-0.0101	0.0123	0.0160	Pentatricopeptide repeat (PPR-like) superfamily protein		5.4	4.0	1.8	10.0	10.6	5.6
Ca_24428	-0.0112	0.0078	0.0136	Pentatricopeptide repeat (PPR-like) superfamily protein	GO:0005515	7.0	3.8	3.4	9.9	11.5	11.1
Ca_26805	-0.0022	0.0135	0.0137	Pentatricopeptide repeat (PPR-like) superfamily protein	GO:0005515	27.7	0.0	3.2	8.1	7.7	12.9
Ca_27579	-0.0129	0.0049	0.0138	Pentatricopeptide repeat (PPR-like) superfamily protein		0.0	0.0	0.0	0.0	5.2	6.4
Ca_27774	-0.0120	0.0089	0.0150	Pentatricopeptide repeat (PPR-like) superfamily protein	GO:0005515	1.5	0.0	0.0	5.4	3.4	7.3
Ca_29950	-0.0102	0.0091	0.0137	PENTATRICOPEPTIDE REPEAT 596		0.0	0.0	0.0	5.8	1.7	0.0
Ca_26711	-0.0102	0.0093	0.0138	pentatricopeptide repeat-containing protein At5g66520-like [Glycine max]		2.2	0.0	0.0	0.0	5.3	4.8
Ca_16150	-0.0028	0.0133	0.0136	peptide transporter 1	GO:0005215 GO:0006810 GO:0016020	219.4	78.8	62.1	83.5	147.9	96.1
Ca_29048	-0.0142	0.0032	0.0146	peptidyl-prolyl cis-trans isomerase n=1 Tax=Enterobacteriaceae bacterium LSJC7 RepID=UPI000378BF66	GO:0003755 GO:0006457 GO:0015031 GO:0016853 GO:0030288 GO:0042277 GO:0042710 GO:0043165 GO:0050821 GO:0051082	7.1	8.1	13.1	22.1	16.9	19.1
Ca_17392	-0.0134	0.0060	0.0147	Peroxidase superfamily protein	GO:0004601 GO:0006979 GO:0020037 GO:0055114	28.4	21.6	31.3	38.7	75.2	56.6
Ca_28757	-0.0108	0.0083	0.0136	Peroxiredoxin n=1 Tax=Halorhodospira halochloris str. A RepID=W8KGT6_HALHR	GO:0006950	1.5	1.6	0.0	8.7	0.0	6.8
Ca_02231	-0.0044	0.0139	0.0145	Peroxisomal membrane 22 kDa (Mpv17/PMP22) family protein	GO:0016021	439.6	293.4	300.4	296.8	359.4	506.9

Ca_24962	0.0013	0.0137	0.0137	PHD-finger protein	GO:0005515 GO:0008270	352.5	158.8	158.1	191.3	183.7	170.0
Ca_16923	-0.0060	0.0127	0.0140	phosphate transporter 1	GO:0005215 GO:0006810 GO:0016020 GO:0016021 GO:0022857 GO:0055085	1008.4	529.8	481.6	677.3	747.8	962.6
Ca_20068	-0.0014	0.0149	0.0150	phosphate transporter PHO1 homolog 1-like isoform 1 [Glycine max]	GO:0016021	824.6	200.2	155.6	251.1	211.2	421.7
Ca_25665	-0.0132	0.0036	0.0137	Phosphate-responsive 1 family protein		12.0	10.5	25.1	30.1	50.2	39.9
Ca_27837	-0.0040	0.0137	0.0143	phosphoenolpyruvate carboxylase 4	GO:0003824 GO:0006099 GO:0008964 GO:0015977	7.3	0.0	0.0	6.7	4.8	0.0
Ca_11910	-0.0142	0.0101	0.0174	phosphopantothenoylcysteine decarboxylase subunit SIS2-like [Glycine max]		1.7	0.0	0.0	5.8	13.1	8.7
Ca_14497	-0.0035	0.0146	0.0150	photosystem II reaction center PsbP family protein	GO:0005509 GO:0009523 GO:0009654 GO:0015979 GO:0019898	128.0	22.1	24.4	55.9	52.9	51.3
Ca_00468	-0.0066	0.0119	0.0136	p-hydroxybenzoic acid efflux pump subunit aaeB n=1 Tax=Medicago truncatula RepID=G7IHP1_MEDTR		37.1	14.8	24.4	29.2	33.1	34.1
Ca_19388	-0.0129	0.0103	0.0165	PIF1-like helicase	GO:0000723 GO:0003678 GO:0006281	8.6	12.7	4.6	27.5	18.9	11.9
Ca_05973	-0.0146	0.0069	0.0162	pirin		10.1	15.7	12.3	20.7	21.4	27.8
Ca_23610	-0.0121	0.0110	0.0164	Plant calmodulin-binding protein-related	GO:0005516	2.8	2.6	0.0	3.7	5.3	6.6
Ca_24276	-0.0143	0.0080	0.0164	Plant protein of unknown function (DUF946)		2.2	0.0	0.0	3.3	6.8	7.5
Ca_21729	-0.0137	0.0143	0.0198	Plant self-incompatibility protein S1 family		3.5	0.0	0.0	3.6	4.8	4.4
Ca_03337	-0.0128	0.0098	0.0161	plant/MEB5-like protein		136.2	104.8	112.9	148.0	143.3	179.8
Ca_01807	-0.0038	0.0126	0.0132	plasma-membrane associated cation-binding protein 1	GO:0046658 GO:0051716	192.6	144.5	101.9	118.8	175.6	180.8
Ca_11291	-0.0134	0.0117	0.0178	pleiotropic drug resistance 11	GO:0000166 GO:0005524 GO:0016020 GO:0016887 GO:0017111	130.5	119.2	112.3	126.2	182.8	182.8
Ca_15028	-0.0103	0.0113	0.0153	pleiotropic drug resistance 12	GO:0000166 GO:0005524 GO:0016020 GO:0016887 GO:0017111	302.5	232.0	178.7	269.6	610.0	308.2
Ca_22817	-0.0111	0.0087	0.0141	P-loop containing nucleoside triphosphate hydrolases superfamily protein	GO:0008146	90.5	88.2	112.5	123.5	129.0	138.8
Ca_02418	-0.0166	0.0080	0.0184	Pollen Ole e 1 allergen and extensin family protein	GO:0005615	22.1	9.8	21.9	58.2	39.1	58.7
Ca_15491	-0.0094	0.0098	0.0136	poly(A) polymerase 1	GO:0003723 GO:0004652 GO:0005634 GO:0016779 GO:0031123 GO:0043631	107.6	81.7	110.7	130.5	129.8	125.7
Ca_11942	-0.0122	0.0064	0.0137	polygalacturonase 4	GO:0004650 GO:0005975	9.9	6.8	8.5	16.1	15.1	21.0
Ca_04655	-0.0110	0.0109	0.0155	polygalacturonase QRT3-like [Glycine max]		2.7	8.3	2.1	13.1	9.9	9.5
Ca_11972	-0.0137	0.0038	0.0142	polygalacturonase-like [Glycine max]	GO:0004650 GO:0005975	21.3	35.8	38.1	51.7	56.7	53.0
Ca_26840	-0.0139	0.0042	0.0145	Polymerase n=1 Tax=Medicago truncatula RepID=G7I2J6_MEDTR		21.1	25.5	23.3	43.2	65.7	44.3
Ca_05817	-0.0077	0.0126	0.0147	polyvinylalcohol dehydrogenase-like protein		319.7	160.9	208.3	242.0	280.1	335.5
Ca_24089	-0.0133	0.0074	0.0152	probable 2-oxoglutarate/Fe(II)-dependent dioxygenase-like [Glycine max]	GO:0016491 GO:0016706 GO:0055114	4.7	11.8	6.6	17.3	28.4	17.2
Ca_25458	-0.0131	0.0020	0.0132	probable 2-oxoglutarate/Fe(II)-dependent dioxygenase-like [Glycine max]	GO:0016491 GO:0016706 GO:0055114	22.5	31.1	37.7	62.3	42.8	47.9
Ca_19970	-0.0138	0.0015	0.0139	probable calcium-binding protein CML16 [Glycine max]	GO:0005509	8.6	17.4	35.0	60.8	45.6	43.5
Ca_03287	-0.0130	0.0049	0.0139	probable calcium-binding protein CML20 [Glycine max]	GO:0005509 GO:0005515	19.0	29.1	22.0	31.3	58.6	42.5
Ca_04878	-0.0089	0.0101	0.0134	probable galacturonosyltransferase-like 9-like [Glycine max]	GO:0016757	131.9	114.2	103.2	112.6	150.7	166.7
Ca_21428	-0.0038	0.0131	0.0137	probable glycerophosphoryl diester phosphodiesterase 3-like [Glycine max]	GO:0003824 GO:0005975 GO:0006071 GO:0006629 GO:0008081 GO:0008889 GO:0030246	533.6	341.9	274.3	312.8	458.7	470.9
Ca_08216	-0.0037	0.0146	0.0151	probable pectinesterase/pectinesterase inhibitor 21-like [Glycine max]	GO:0004857 GO:0005618 GO:0030599 GO:0042545	114.2	53.1	38.5	66.3	72.2	66.7
Ca_24502	-0.0136	0.0045	0.0144	probable pectinesterase/pectinesterase inhibitor 21-like [Glycine max]	GO:0005618 GO:0030599 GO:0042545	5.6	4.7	12.2	13.7	23.0	17.4
Ca_10286	-0.0105	0.0094	0.0141	probable polygalacturonase-like [Glycine max]	GO:0004650 GO:0005975	4.6	5.4	0.0	5.9	11.7	9.3
Ca_29129	-0.0155	0.0077	0.0173	Prolipoprotein diacylglyceryl transferase n=19 Tax=Enterobacteriaceae RepID=D4GLM9_PANAM	GO:0003824 GO:0009249 GO:0016020 GO:0016310 GO:0016757 GO:0016772 GO:0042158	5.8	14.3	6.6	32.8	37.5	18.7
Ca_17215	-0.0149	0.0121	0.0193	Proteasome maturation factor UMP1		44.4	32.0	30.7	505.7	46.0	330.9
Ca_01354	-0.0109	0.0092	0.0142	protein arginine methyltransferase 10	GO:0005737 GO:0006479 GO:0008168 GO:0008276	4.8	1.7	0.0	5.4	5.9	6.4
Ca_07118	-0.0030	0.0132	0.0135	protein gar2-like [Glycine max]		77.0	20.9	24.9	36.4	37.6	39.4
Ca_22562	-0.0152	0.0055	0.0162	protein HAPLESS 2-like [Glycine max]		51.8	66.9	58.5	112.7	82.3	98.3
Ca_25547	-0.0145	0.0083	0.0167	protein kinase [Glycine max]	GO:0004672 GO:0005524 GO:0006468 GO:0016772	4.0	3.7	0.0	9.0	8.8	11.6
Ca_03291	-0.0144	0.0073	0.0161	protein kinase 2A	GO:0004672 GO:0004674 GO:0005524 GO:0006468 GO:0016772	319.3	334.7	460.9	694.9	416.9	786.8
Ca_06456	-0.0081	0.0109	0.0136	protein kinase family protein		1064.8	707.5	936.7	1137.5	938.6	1168.4
Ca_06548	-0.0097	0.0101	0.0140	Protein kinase family protein	GO:0004672 GO:0004674 GO:0005524 GO:0006468 GO:0016772	505.1	463.6	142.7	547.7	539.3	441.2
Ca_00225	-0.0066	0.0125	0.0142	Protein kinase superfamily protein	GO:0004672 GO:0005524 GO:0006468 GO:0016772 GO:0016998	114.8	98.9	79.0	102.7	153.0	112.1
Ca_02244	-0.0028	0.0127	0.0131	Protein kinase superfamily protein	GO:0004672 GO:0004674 GO:0005524 GO:0006468 GO:0016772	459.4	106.5	120.3	125.6	241.5	239.3
Ca_03381	-0.0127	0.0060	0.0140	Protein kinase superfamily protein	GO:0004672 GO:0005524 GO:0006468 GO:0016772	0.0	0.0	0.0	0.0	4.5	5.0
Ca_09953	-0.0061	0.0123	0.0137	Protein kinase superfamily protein	GO:0004672 GO:0004674	135.2	96.4	93.3	116.0	125.5	120.6
Ca_17724	-0.0017	0.0132	0.0133	Protein kinase superfamily protein	GO:0004672 GO:0005524 GO:0006468 GO:0016772	76.3	37.1	43.3	49.4	59.1	50.8
Ca_20674	-0.0120	0.0068	0.0138	Protein kinase superfamily protein	GO:0004672 GO:0004674 GO:0005524 GO:0006468 GO:0016772	150.6	96.3	105.5	147.7	156.1	190.3

Ca_23504	-0.0051	0.0140	0.0149	Protein kinase superfamily protein	GO:0004672 GO:0004674 GO:0005524 GO:0006468 GO:0016772	360.1	230.1	319.5	359.4	319.6	312.9
Ca_23655	-0.0128	0.0099	0.0162	Protein kinase superfamily protein	GO:0004672 GO:0006468 GO:0016772	3.9	11.2	0.0	10.0	11.8	12.3
Ca_26010	-0.0026	0.0142	0.0144	Protein kinase superfamily protein	GO:0004672 GO:0004674 GO:0005524 GO:0006468 GO:0016772	953.9	449.1	454.9	526.6	599.2	735.4
Ca_12964	-0.0097	0.0117	0.0152	protein NYNRIN-like [Glycine max]	GO:0003676 GO:0015074	26.6	18.2	15.5	36.1	17.3	44.7
Ca_11363	-0.0119	0.0060	0.0134	protein NYNRIN-like [Glycine max]		1.8	0.0	0.0	0.0	9.0	7.7
Ca_21124	-0.0145	0.0064	0.0159	Protein of unknown function (DUF1442)		3.4	11.9	18.1	63.2	21.1	40.6
Ca_07229	-0.0154	0.0052	0.0163	Protein of Unknown Function (DUF239)		22.6	35.1	30.7	63.5	53.4	50.6
Ca_14819	-0.0011	0.0136	0.0136	Protein of Unknown Function (DUF239)		119.8	67.1	61.1	75.9	80.7	79.2
Ca_14821	-0.0035	0.0127	0.0132	Protein of Unknown Function (DUF239)		60.7	23.4	17.3	33.4	43.1	32.1
Ca_09512	-0.0057	0.0144	0.0155	Protein of unknown function (DUF3511)		31.0	24.8	12.3	26.8	21.0	33.3
Ca_17173	0.0000	0.0133	0.0133	Protein of unknown function (DUF506)		423.1	147.1	123.9	148.4	225.1	152.6
Ca_05246	-0.0114	0.0120	0.0165	Protein of unknown function (DUF688)		42.9	34.4	20.5	50.9	53.8	61.5
Ca_11153	-0.0132	0.0029	0.0135	Protein phosphatase 2C family protein	GO:0003824	16.8	31.4	21.2	33.2	39.3	42.6
Ca_22740	0.0016	0.0133	0.0134	protein phosphatase methylesterase 1-like [Glycine max]	GO:0003824 GO:0006482 GO:0052689	188.2	46.9	49.8	70.9	59.0	64.0
Ca_15809	-0.0047	0.0129	0.0137	protein SCARECROW-like [Glycine max]		140.9	94.6	79.9	88.6	162.7	104.0
Ca_00615	-0.0090	0.0099	0.0133	protein serine/threonine kinases		2328.6	1960.5	2165.9	2312.0	2720.3	2974.2
Ca_16079	-0.0056	0.0131	0.0143	protein serine/threonine kinases	GO:0004672 GO:0004674 GO:0005524 GO:0006468 GO:0016772 GO:0030246	356.5	267.4	279.8	316.3	347.6	326.8
Ca_19286	-0.0092	0.0117	0.0149	protein serine/threonine phosphatases	GO:0004672 GO:0004674 GO:0005524 GO:0006468 GO:0016772	50.1	67.9	38.7	65.0	80.1	56.1
Ca_27061	-0.0135	0.0012	0.0135	pumilio 2		21.8	54.5	28.3	68.2	70.7	100.1
Ca_08192	-0.0087	0.0117	0.0146	pumilio-family RNA-binding repeatprotein	GO:0003723 GO:0005488	40.4	18.8	17.4	25.0	41.6	42.7
Ca_06610	-0.0007	0.0137	0.0137	purple acid phosphatase 8-like [Glycine max]	GO:0016787	456.1	102.9	130.1	158.6	192.3	193.4
Ca_21103	-0.0143	0.0048	0.0151	putative axial regulator YABBY 2-like isoform X3 [Glycine max]		7.7	8.4	8.2	13.7	16.0	14.3
Ca_17649	-0.0061	0.0137	0.0150	putative glycerol-3-phosphate transporter 1-like isoform X2 [Glycine max]	GO:0005215 GO:0006810 GO:0016021 GO:0055085	1134.9	370.1	644.5	673.3	973.7	904.2
Ca_20291	-0.0113	0.0147	0.0186	putative indole-3-acetic acid-amido synthetase GH3.9		484.6	263.2	331.4	563.7	666.0	743.2
Ca_06249	-0.0097	0.0087	0.0130	putative ribonuclease H protein At1g65750-like [Glycine max]	GO:0003676 GO:0004523	10.1	7.5	5.5	10.8	12.6	10.0
Ca_25029	-0.0125	0.0062	0.0140	pyruvate decarboxylase-2		3.7	4.1	3.6	7.1	8.1	13.1
Ca_17118	-0.0141	0.0051	0.0150	RAB GTPase activator protein n=11 Tax=Brassicaceae RepID=B3H765_ARATH	GO:0005097 GO:0032313	21.8	25.0	19.3	31.4	28.6	38.2
Ca_12446	-0.0041	0.0138	0.0144	rab3 GTPase-activating protein catalytic subunit-like isoform X1 [Glycine max]	GO:0005097	200.4	147.2	148.4	203.8	153.4	172.0
Ca_08827	-0.0125	0.0039	0.0131	RAN binding protein 1	GO:0046907	14.0	2.4	25.9	17.2	63.4	160.3
Ca_22098	-0.0109	0.0072	0.0131	Rap1-interacting factor 1 amine-terminal protein	GO:0005488	518.5	662.4	469.4	637.6	713.8	953.2
Ca_13105	-0.0124	0.0102	0.0161	receptor kinase 2	GO:0004672 GO:0004674 GO:0005524 GO:0006468 GO:0016772	200.9	121.9	298.0	302.7	426.9	355.0
Ca_20193	-0.0090	0.0107	0.0140	receptor kinase 2	GO:0004672 GO:0004674 GO:0005524 GO:0006468	129.4	103.7	126.7	145.8	136.8	189.3
Ca_15301	-0.0122	0.0050	0.0132	receptor lectin kinase	GO:0004672 GO:0004674	135.9	124.1	147.8	182.8	169.6	227.8
Ca_24226	-0.0087	0.0111	0.0141	receptor-like kinase 1	GO:0004672 GO:0005524 GO:0006468 GO:0016772	102.7	61.9	93.1	121.8	84.1	106.3
Ca_29260	-0.0020	0.0154	0.0155	receptor-like kinase 1	GO:0004672 GO:0005515 GO:0005524 GO:0006468 GO:0016772	32.0	0.0	2.1	5.3	9.6	8.6
Ca_02548	-0.0124	0.0048	0.0133	receptor-like kinase 902	GO:0004672 GO:0005524 GO:0006468 GO:0016772	16.1	16.8	13.6	22.9	35.9	28.2
Ca_23322	-0.0108	0.0119	0.0161	receptor-like protein kinase 1	GO:0004672	66.5	45.1	51.7	81.5	71.0	71.1
Ca_03341	-0.0136	0.0032	0.0140	receptor-like protein kinase 2	GO:0005515	19.9	27.6	16.1	27.5	49.5	50.0
Ca_16674	-0.0121	0.0106	0.0161	receptor-like protein kinase 2-like [Glycine max]	GO:0005515	49.0	30.2	54.2	107.9	59.2	138.9
Ca_21552	-0.0093	0.0092	0.0131	receptor-like protein kinase 2-like [Glycine max]	GO:0005515	77.9	83.2	66.8	83.4	110.2	94.0
Ca_10442	-0.0042	0.0160	0.0165	receptor-like protein kinase 4	GO:0004672 GO:0004674 GO:0005524 GO:0006468 GO:0016772 GO:0030247	174.5	63.9	61.7	88.0	99.0	111.7
Ca_24347	0.0005	0.0134	0.0134	receptor-like protein kinase 4	GO:0004672 GO:0006468 GO:0016772	88.1	35.3	25.4	35.6	41.5	40.6
Ca_15726	0.0005	0.0134	0.0134	Regulator of Vps4 activity in the MVB pathway protein		304.7	151.8	171.3	204.5	169.1	191.6
Ca_27894	-0.0128	0.0087	0.0155	Replicase n=18 Tax=Beet necrotic yellow vein virus RepID=RDRP_BNYVS		6.5	12.2	6.9	16.1	16.8	17.3
Ca_23280	-0.0039	0.0131	0.0137	replication factor C subunit 3	GO:0003677 GO:0006260	176.8	56.5	56.0	90.3	126.4	91.6
Ca_11497	-0.0146	0.0031	0.0149	Retrotransposon Tto1 DNA n=2 Tax=Nicotiana tabacum RepID=Q9ZRJ0_TOBAC		11.7	11.2	12.6	19.6	27.8	21.0
Ca_13088	-0.0053	0.0140	0.0149	retrotransposon-like protein 1-like [Glycine max]		78.1	47.0	25.2	48.5	63.3	59.6
Ca_15004	-0.0023	0.0136	0.0138	Rhamnogalacturonate lyase family protein	GO:0030246	139.8	80.9	78.4	121.1	98.8	87.6
Ca_15368	-0.0147	0.0020	0.0148	Rhodanese/Cell cycle control phosphatase superfamily protein		59.3	74.9	84.9	119.8	115.1	119.6
Ca_00581	-0.0044	0.0125	0.0133	riboflavin kinase/fmn hydrolase	GO:0008152 GO:0008531 GO:0009231 GO:0016787	223.0	70.9	81.5	150.7	198.4	101.1
Ca_02957	-0.0070	0.0137	0.0154	ribosome biogenesis protein TSR3 homolog isoform X2 [Glycine max]		155.3	121.3	129.0	187.6	174.4	120.7
Ca_25005	-0.0088	0.0154	0.0177	ribulose bisphosphate carboxylase/oxygenase activase	GO:0005524	234.5	189.5	151.4	211.3	267.1	207.2
Ca_13490	-0.0139	0.0009	0.0139	RING-H2 finger protein 2B	GO:0005515 GO:0008270	23.4	29.1	35.0	50.3	55.8	56.2
Ca_27171	-0.0109	0.0071	0.0130	RING-H2 zinc finger protein		3.7	1.3	5.2	7.5	4.9	8.3
Ca_06520	-0.0124	0.0038	0.0130	Ripening related protein family		5.4	2.3	7.5	12.6	17.2	9.7
Ca_23612	0.0000	0.0139	0.0139	RNA polymerase-associated protein RTF1 homolog [Glycine max]	GO:0003677 GO:0005634 GO:0006352 GO:0016570	448.6	274.2	189.4	298.6	276.1	227.7
Ca_06666	-0.0138	0.0116	0.0180	RNA-binding protein 38-like isoform X2 [Glycine max]	GO:0000166 GO:0003676	0.7	1.8	0.0	6.3	5.5	6.2
Ca_25957	-0.0106	0.0101	0.0146	RNA-binding region RNP-1 protein		15.6	8.3	11.8	21.4	14.9	20.5

Ca_27931	-0.0125	0.0074	0.0145	RNA-directed DNA polymerase (Reverse transcriptase)	GO:0003723 GO:0003964 GO:0004190 GO:0006278 GO:0006508	0.0	7.5	0.0	3.7	21.4	11.0
Ca_19921	-0.0123	0.0048	0.0132	RNA-directed DNA polymerase homolog [Glycine max]	GO:0003723 GO:0003964 GO:0006278	37.0	67.0	50.4	85.1	73.1	64.4
Ca_26626	-0.0144	0.0009	0.0144	RNA-directed DNA polymerase homolog [Glycine max]	GO:0003723 GO:0003964 GO:0006278	15.6	14.3	24.3	35.1	30.5	37.8
Ca_03616	-0.0139	0.0092	0.0167	RNA-directed DNA polymerase homolog [Glycine max]		0.0	2.3	0.0	4.4	6.6	6.1
Ca_23409	-0.0086	0.0122	0.0150	ROP guanine nucleotide exchange factor 5	GO:0005089	77.4	65.2	47.0	54.3	88.8	115.1
Ca_16438	-0.0135	0.0043	0.0141	Rubber elongation factor protein (REF)		2.2	1.3	4.2	7.2	6.8	11.9
Ca_16262	-0.0051	0.0139	0.0148	S-adenosyl-L-methionine-dependent methyltransferases superfamily protein		128.5	97.3	67.3	103.6	103.9	120.2
Ca_17494	-0.0133	0.0078	0.0154	SAUR-like auxin-responsive protein family		0.0	0.0	0.0	0.0	1.4	1.1
Ca_18602	-0.0077	0.0152	0.0170	scarecrow-like transcription factor PAT1-like [Glycine max]		140.3	36.2	68.6	97.9	90.4	117.6
Ca_26068	-0.0067	0.0149	0.0163	SCP1-like small phosphatase 4	GO:0005515 GO:0016791	264.2	184.5	200.6	215.2	245.4	264.5
Ca_07611	-0.0138	0.0116	0.0180	SEC-C motif protein		0.0	0.0	0.0	7.7	5.0	2.7
Ca_17126	-0.0134	0.0006	0.0134	seed biotin-containing protein SBP65-like [Glycine max]		113.3	155.1	128.8	162.6	188.8	202.0
Ca_02487	-0.0141	0.0087	0.0166	serine carboxypeptidase-like 19	GO:0004185 GO:0006508	146.3	144.7	191.2	270.1	234.6	213.4
Ca_05722	-0.0139	0.0052	0.0148	serine carboxypeptidase-like 20	GO:0004185 GO:0006508	18.8	20.8	17.6	27.6	30.9	46.8
Ca_10189	-0.0065	0.0128	0.0143	serine carboxypeptidase-like 27	GO:0004185 GO:0006508	196.5	140.0	134.5	154.5	211.4	205.5
Ca_18395	-0.0140	0.0065	0.0154	serine carboxypeptidase-like 48	GO:0004185 GO:0006508	33.4	42.9	30.8	48.3	50.7	47.9
Ca_08495	-0.0167	0.0074	0.0183	serine/threonine-protein kinase TIO-like [Glycine max]	GO:0004672 GO:0004674 GO:0005524 GO:0006468 GO:0016772	566.5	661.9	652.7	1023.5	970.7	883.9
Ca_01632	-0.0137	0.0089	0.0164	serine/threonine-protein phosphatase 7 long form homolog [Glycine max]		9.7	7.5	8.0	12.1	14.4	16.4
Ca_04534	-0.0132	0.0085	0.0157	serine/threonine-protein phosphatase 7 long form homolog [Glycine max]		12.0	11.4	12.3	20.2	14.8	22.1
Ca_06192	-0.0135	0.0070	0.0152	serine/threonine-protein phosphatase 7 long form homolog [Glycine max]		24.7	16.0	20.7	41.7	29.4	35.1
Ca_08094	-0.0134	0.0122	0.0181	serine/threonine-protein phosphatase 7 long form homolog [Glycine max]		6.2	0.0	0.0	9.9	17.4	8.1
Ca_11475	-0.0116	0.0096	0.0150	serine/threonine-protein phosphatase 7 long form homolog [Glycine max]		0.0	0.0	0.0	5.7	0.0	2.7
Ca_11929	-0.0145	0.0127	0.0193	serine/threonine-protein phosphatase 7 long form homolog [Glycine max]		86.0	71.1	76.5	154.8	168.5	167.0
Ca_12500	-0.0114	0.0079	0.0138	serine/threonine-protein phosphatase 7 long form homolog [Glycine max]		0.0	0.0	0.0	1.7	0.0	3.8
Ca_23864	-0.0145	0.0100	0.0177	serine/threonine-protein phosphatase 7 long form homolog [Glycine max]		3.5	0.0	3.0	4.8	8.5	6.1
Ca_26211	-0.0133	0.0062	0.0147	serine/threonine-protein phosphatase 7 long form homolog [Glycine max]		0.0	0.0	0.0	0.0	5.3	7.1
Ca_26966	-0.0006	0.0131	0.0131	serine/threonine-protein phosphatase 7 long form homolog [Glycine max]		180.0	62.3	62.6	80.6	88.6	91.8
Ca_27670	-0.0150	0.0109	0.0185	serine/threonine-protein phosphatase 7 long form homolog [Glycine max]		0.0	0.0	0.0	2.7	3.6	4.6
Ca_18272	-0.0122	0.0066	0.0139	serine/threonine-protein phosphatase 7 long form homolog [Glycine max]		1.1	0.0	0.0	0.0	6.0	6.3
Ca_17409	-0.0045	0.0162	0.0168	shikimate O-hydroxycinnamoyltransferase [Glycine max]	GO:0016747	122.2	60.0	63.7	77.2	93.2	91.4
Ca_20833	-0.0152	0.0070	0.0167	short-chain dehydrogenase/reductase	GO:0008152 GO:0016491	14.9	12.4	14.5	18.3	33.9	31.6
Ca_29006	-0.0105	0.0088	0.0137	short-chain dehydrogenase/reductase	GO:0008152 GO:0016491	0.0	0.0	0.0	5.0	1.9	0.0
Ca_28657	-0.0146	0.0077	0.0165	short-chain dehydrogenase-reductase B	GO:0008152 GO:0016491	4.5	4.9	0.0	10.8	7.7	13.4
Ca_11147	-0.0039	0.0167	0.0172	SIGNAL PEPTIDE PEPTIDASE-LIKE 5	GO:0004190 GO:0016021	299.0	93.2	73.3	123.9	173.0	170.0
Ca_03183	-0.0011	0.0135	0.0135	Signal transduction histidine kinase		865.1	530.9	586.7	661.7	648.3	659.8
Ca_24613	-0.0129	0.0066	0.0145	Sodium Bile acid symporter family	GO:0005515 GO:0006814 GO:0008508 GO:0016020	3.6	1.3	0.0	3.5	8.2	6.7
Ca_09862	-0.0070	0.0128	0.0146	Sodium/calcium exchanger n=2 Tax=Papilionoideae RepID=G7IF47_MEDTR	GO:0016021 GO:0055085	662.5	516.1	546.3	711.8	598.9	759.6
Ca_17723	-0.0134	0.0060	0.0147	SOUL heme-binding family protein		7.1	14.9	5.6	18.8	14.8	21.5
Ca_24326	-0.0117	0.0098	0.0152	spermidine hydroxycinnamoyl transferase-like [Glycine max]	GO:0016747	0.0	0.0	0.0	5.6	2.7	0.0
Ca_13494	-0.0129	0.0155	0.0201	spermidine synthase 1	GO:0003824	24.1	24.8	17.4	36.2	28.0	31.4
Ca_14729	-0.0056	0.0151	0.0161	SPX domain gene 2		495.0	129.7	134.9	197.0	322.8	329.0
Ca_28941	-0.0136	0.0096	0.0166	S-ribosylhomocysteine lyase n=19 Tax=Enterobacteriaceae RepID=U3TXQ3_9ENTR	GO:0003824 GO:0005506 GO:0009372 GO:0043768 GO:0046872	1.0	0.0	0.0	4.9	12.3	5.2
Ca_18212	-0.0107	0.0087	0.0138	stress up-regulated Nod 19 protein		7.8	7.1	8.7	15.2	12.3	11.0
Ca_00729	-0.0139	0.0051	0.0148	strictosidine synthase-like 3	GO:0009058 GO:0016844	12.1	16.7	13.3	26.0	23.0	21.8
Ca_03691	-0.0087	0.0114	0.0144	subtilisin-like serine protease 2	GO:0004252 GO:0006508	182.8	98.6	106.9	146.8	183.9	171.5
Ca_22758	-0.0140	0.0042	0.0146	subtilisin-like serine protease 2	GO:0004252 GO:0006508 GO:0042802 GO:0043086	32.4	69.2	67.7	58.9	370.2	371.9
Ca_23729	-0.0148	0.0083	0.0170	subtilisin-like serine protease 2	GO:0004252 GO:0006508	0.0	0.0	0.0	12.1	3.8	8.1
Ca_21953	-0.0088	0.0105	0.0137	sugar transport protein 7-like [Glycine max]	GO:0005215 GO:0006810 GO:0016020 GO:0016021 GO:0022857 GO:0022891 GO:0055085	5.5	3.6	5.5	11.5	5.7	7.4
Ca_00989	-0.0128	0.0101	0.0163	sugar transporter 11	GO:0016021 GO:0022857 GO:0055085	0.0	0.0	0.0	8.6	8.0	0.0
Ca_13282	-0.0159	0.0064	0.0172	sulfate transporter 1	GO:0008271 GO:0008272 GO:0015116 GO:0016020 GO:0016021 GO:0055085	32.3	44.0	52.7	70.0	78.3	75.8
Ca_16139	-0.0135	0.0052	0.0144	Sulfite exporter TauE/SafE family protein	GO:0016021	66.6	82.2	165.5	288.0	133.9	205.5
Ca_22153	-0.0129	0.0032	0.0133	temperature-induced lipocalin	GO:0005215	17.9	32.9	27.0	45.9	51.7	43.1
Ca_01994	-0.0147	0.0008	0.0147	Tetratricopeptide repeat (TPR)-like superfamily protein	GO:0005515	53.7	68.4	73.5	93.5	92.3	90.7
Ca_16474	-0.0121	0.0057	0.0134	TGACG-motif-binding factor [Glycine max]	GO:0003700 GO:0005515 GO:0006355 GO:0008270 GO:0043565	6.0	6.4	2.2	8.5	20.1	21.7
Ca_25045	-0.0064	0.0113	0.0130	TGACG-sequence-specific DNA-binding protein TGA-2.1-like isoform X1 [Glycine max]	GO:0003700 GO:0006351 GO:0006355 GO:0043565	178.5	45.0	85.9	128.9	107.6	114.3

Ca_28793	-0.0105	0.0096	0.0142	Thioredoxin reductase n=1 Tax=Yersinia enterocolitica LC20 RepID=W8UR19_YEREN	GO:0009055 GO:0015035 GO:0016491 GO:0045454	3.2	0.0	0.0	20.6	3.7	6.9
Ca_28708	-0.0152	0.0082	0.0173	Thymidine kinase	GO:0004797 GO:0005524 GO:0006259	2.2	0.0	0.0	11.4	5.5	9.0
Ca_09751	-0.0044	0.0142	0.0149	topless-related protein 1-like isoform X3 [Glycine max]	GO:0005515	142.5	32.0	37.0	65.2	79.3	62.2
Ca_22106	-0.0130	0.0040	0.0136	topless-related protein 4-like isoform X2 [Glycine max]	GO:0005515 GO:0006355	2.0	5.3	4.2	6.6	9.8	9.6
Ca_13777	-0.0136	-0.0004	0.0136	TPR repeat-containing thioredoxin TTL1-like [Glycine max]	GO:0005515	33.5	40.4	62.9	85.9	82.1	74.3
Ca_16385	-0.0121	0.0053	0.0132	TraB family protein		14.0	13.9	17.2	26.7	22.0	32.5
Ca_27150	-0.0125	0.0066	0.0141	transcription elongation factor (TFIIS) family protein		2.6	0.9	0.0	2.7	6.0	5.5
Ca_20865	-0.0023	0.0145	0.0147	transcription factor	GO:0046983	257.9	131.5	123.6	177.0	153.6	183.9
Ca_20347	-0.0016	0.0130	0.0131	transcription factor bHLH130 isoform X2 [Glycine max]	GO:0046983	212.3	68.1	71.9	86.4	118.8	116.2
Ca_04063	-0.0118	0.0096	0.0152	transcription factor bHLH74-like [Glycine max]	GO:0046983	0.0	0.0	0.0	5.5	4.4	0.0
Ca_21623	-0.0127	0.0041	0.0133	transcription factor CYCLOIDEA-like isoform X3 [Glycine max]		57.7	68.4	49.6	67.5	86.7	93.5
Ca_23584	-0.0003	0.0134	0.0134	transcription factor GTE10-like [Glycine max]	GO:0005515	501.3	367.9	332.6	433.8	392.9	370.7
Ca_10776	-0.0116	0.0069	0.0135	Transcription factor jumonji (jmjC) domain-containing protein	GO:0005515	202.8	182.6	317.2	302.4	384.0	359.6
Ca_25079	-0.0041	0.0144	0.0150	transcription factor ORG2-like protein	GO:0003677 GO:0046983	70.5	35.2	20.3	45.7	53.0	43.8
Ca_09445	-0.0109	0.0071	0.0130	transcription factor-related	GO:0046983	0.0	4.0	0.0	10.3	0.0	8.8
Ca_16726	0.0002	0.0131	0.0131	transcriptional corepressor LEUNIG-like isoform X5 [Glycine max]	GO:0005515	160.2	64.2	49.5	78.0	80.1	83.8
Ca_22109	-0.0161	0.0143	0.0215	Transducin family protein / WD-40 repeat family protein	GO:0005515	1.9	1.7	0.6	8.1	9.1	8.0
Ca_21374	-0.0144	0.0035	0.0148	Transducin/WD40 repeat-like superfamily protein	GO:0005515	13.8	13.8	13.7	19.2	37.7	31.3
Ca_03505	-0.0039	0.0129	0.0134	Transmembrane amino acid transporter family protein		133.1	60.4	61.9	86.4	82.8	102.4
Ca_26197	-0.0065	0.0116	0.0134	Transmembrane amino acid transporter family protein		556.7	427.1	252.4	355.1	600.3	588.8
Ca_28344	-0.0128	0.0059	0.0141	Transmembrane pair domain protein n=5 Tax=Pseudomonas RepID=A4XY20_PSEMY		0.0	0.0	0.0	1.8	1.4	2.9
Ca_10849	-0.0141	0.0082	0.0163	trehalose phosphate synthase	GO:0003824 GO:0005992 GO:0008152	0.0	0.0	2.7	4.4	3.9	5.4
Ca_28424	-0.0147	0.0101	0.0178	tRNA uridine 5-carboxymethylaminomethyl modification enzyme MnmG n=3 Tax=Alcaligenes RepID=J0BGJ3_ALCFA	GO:0008033 GO:0050660	0.8	0.0	0.0	8.4	1.6	3.4
Ca_14732	-0.0032	0.0139	0.0142	ubiquitin carboxyl-terminal hydrolase	GO:0004843 GO:0006511	242.2	160.6	138.2	163.9	194.6	198.4
Ca_03672	-0.0144	0.0042	0.0150	ubiquitin-conjugating enzyme 28	GO:0016881	29.9	47.9	56.0	79.0	64.8	83.0
Ca_09379	-0.0141	0.0118	0.0184	U-box domain-containing protein 4 [Glycine max]	GO:0005488 GO:0005515	0.0	0.0	0.0	4.7	1.4	4.1
Ca_14512	-0.0123	0.0101	0.0159	U-box domain-containing protein 44-like [Glycine max]	GO:0000151 GO:0004842 GO:0005488 GO:0005515 GO:0016567	5.0	4.3	5.5	9.5	8.9	9.5
Ca_09448	-0.0120	0.0075	0.0141	UBX domain-containing protein		0.0	0.0	0.0	0.0	5.0	6.2
Ca_27428	-0.0173	0.0105	0.0202	UBX domain-containing protein	GO:0005515	7.6	5.5	3.4	29.2	54.0	40.0
Ca_16265	-0.0121	0.0065	0.0138	UBX domain-containing protein 1-like isoform X2 [Glycine max]	GO:0005515	0.0	0.0	0.0	7.8	0.8	6.7
Ca_19091	-0.0132	0.0025	0.0134	UDP-glucosyltransferase family protein	GO:0008152 GO:0016758	21.4	36.3	29.8	45.2	62.3	46.4
Ca_04754	-0.0029	0.0128	0.0131	UDP-Glycosyltransferase superfamily protein	GO:0008152 GO:0016758	157.0	42.2	69.2	80.3	105.8	80.1
Ca_06658	-0.0116	0.0091	0.0148	UDP-Glycosyltransferase superfamily protein	GO:0008152 GO:0016758	45.8	54.5	35.5	56.1	55.4	66.2
Ca_16526	-0.0131	0.0150	0.0200	UDP-Glycosyltransferase superfamily protein	GO:0008152 GO:0016758	252.5	187.6	176.2	278.3	287.4	274.9
Ca_22394	-0.0130	0.0019	0.0131	UDP-Glycosyltransferase superfamily protein	GO:0008152 GO:0016758	12.3	34.6	41.4	49.8	46.1	47.3
Ca_26170	-0.0147	0.0080	0.0168	UDP-Glycosyltransferase superfamily protein	GO:0008152 GO:0016758	3.7	2.1	5.9	7.1	7.6	9.7
Ca_28809	-0.0109	0.0122	0.0163	UDP-N-acetylglucosamine 1-carboxyvinyltransferase n=4 Tax=Pantoea RepID=E1SC55_PANVC	GO:0003824 GO:0008760 GO:0016765 GO:0019277	3.4	0.0	0.0	9.7	0.0	5.7
Ca_05340	-0.0117	0.0064	0.0133	uncharacterized mitochondrial protein AtMg00810-like [Glycine max]		10.8	15.3	11.5	18.2	16.6	27.2
Ca_11222	-0.0111	0.0099	0.0149	uncharacterized mitochondrial protein AtMg00810-like [Glycine max]		0.7	0.0	0.0	5.3	0.0	2.0
Ca_11596	-0.0130	0.0040	0.0136	uncharacterized mitochondrial protein AtMg00810-like [Glycine max]		7.7	10.1	14.7	17.1	34.3	16.0
Ca_13049	-0.0107	0.0163	0.0195	uncharacterized mitochondrial protein AtMg00810-like [Glycine max]	GO:0003676	4.5	0.0	0.0	5.9	3.8	3.3
Ca_18096	-0.0127	0.0109	0.0167	uncharacterized mitochondrial protein AtMg00810-like [Glycine max]	GO:0003676 GO:0008270	4.3	0.0	0.0	6.9	5.0	5.4
Ca_18175	-0.0133	0.0126	0.0184	uncharacterized mitochondrial protein AtMg00810-like [Glycine max]	GO:0003676 GO:0008270 GO:0015074	79.1	69.7	65.4	89.5	107.1	118.8
Ca_18206	-0.0037	0.0132	0.0137	uncharacterized mitochondrial protein AtMg00810-like [Glycine max]		63.5	26.2	30.9	41.9	53.9	36.3
Ca_27246	0.0007	0.0133	0.0133	uncharacterized mitochondrial protein AtMg00810-like [Glycine max]		98.0	37.6	23.1	38.2	36.7	38.6
Ca_12618	-0.0056	0.0138	0.0149	uncharacterized mitochondrial protein AtMg00810-like [Glycine max]		42.3	36.0	15.5	39.4	29.9	40.7
Ca_22007	-0.0080	0.0121	0.0145	uncharacterized protein At1g04910-like [Glycine max]		208.6	176.3	159.3	204.4	201.4	218.8
Ca_12059	0.0013	0.0134	0.0134	uncharacterized protein At1g04910-like isoform X1 [Glycine max]		286.8	180.4	150.6	180.6	205.0	181.0
Ca_15289	-0.0075	0.0134	0.0154	uncharacterized protein At1g66480-like [Glycine max]		68.8	35.8	52.3	74.7	81.1	53.7

Ca_02375	-0.0133	0.0026	0.0135	uncharacterized protein At4g15545-like isoform X1 [Glycine max]		17.2	38.4	45.9	68.2	51.5	66.5
Ca_03903	-0.0146	0.0025	0.0148	uncharacterized protein LOC100776307 [Glycine max]		8.8	12.0	13.7	21.2	17.9	23.9
Ca_19801	-0.0127	0.0054	0.0138	uncharacterized protein LOC100776940 isoform X3 [Glycine max]	GO:0008270	32.1	24.8	38.7	54.8	63.1	55.6
Ca_25895	-0.0131	0.0046	0.0139	uncharacterized protein LOC100776940 isoform X4 [Glycine max]		10.4	19.3	33.4	37.1	45.2	52.9
Ca_27125	-0.0063	0.0132	0.0146	uncharacterized protein LOC100778204 isoform X3 [Glycine max]		145.4	89.4	87.3	115.7	109.2	162.0
Ca_19588	-0.0144	0.0096	0.0174	uncharacterized protein LOC100778822 isoform X6 [Glycine max]		2.9	4.0	0.0	6.9	8.0	7.3
Ca_24204	-0.0123	0.0064	0.0138	uncharacterized protein LOC100778822 isoform X6 [Glycine max]		5.2	4.3	1.6	11.4	10.6	12.3
Ca_25460	-0.0135	0.0009	0.0135	uncharacterized protein LOC100778822 isoform X6 [Glycine max]		17.2	14.4	19.8	27.0	46.9	42.7
Ca_27893	-0.0140	0.0010	0.0141	uncharacterized protein LOC100778822 isoform X6 [Glycine max]		14.9	21.1	30.3	33.0	53.8	56.1
Ca_04195	-0.0076	0.0123	0.0144	uncharacterized protein LOC100782725 [Glycine max]		1053.4	191.6	418.3	1007.7	1013.0	626.2
Ca_12271	-0.0070	0.0141	0.0157	uncharacterized protein LOC100784968 isoform X1 [Glycine max]		129.9	92.5	95.5	143.9	101.8	128.5
Ca_15505	-0.0046	0.0124	0.0132	uncharacterized protein LOC100785837 [Glycine max]		113.4	55.3	53.5	64.0	82.2	98.1
Ca_27588	-0.0111	0.0100	0.0150	uncharacterized protein LOC100786695 [Glycine max]		10.5	3.5	3.1	7.7	13.5	10.8
Ca_23506	-0.0033	0.0135	0.0139	uncharacterized protein LOC100788333 isoform X5 [Glycine max]		205.3	100.0	84.2	109.0	154.8	123.7
Ca_18417	-0.0074	0.0122	0.0143	uncharacterized protein LOC100790938 isoform X1 [Glycine max]		276.0	245.2	247.2	278.1	323.1	262.3
Ca_03400	-0.0115	0.0070	0.0134	uncharacterized protein LOC100791776 [Glycine max]	GO:0003676 GO:0008270 GO:0015074	9.3	12.0	9.5	19.4	14.0	39.7
Ca_11648	-0.0146	0.0064	0.0159	uncharacterized protein LOC100793937 [Glycine max]		1.8	3.8	8.9	14.9	11.4	12.7
Ca_17350	-0.0065	0.0135	0.0149	uncharacterized protein LOC100794949 isoform X1 [Glycine max]		97.8	43.3	58.5	86.7	79.4	70.8
Ca_18962	-0.0135	0.0142	0.0196	uncharacterized protein LOC100796351 isoform X1 [Glycine max]		54.4	44.8	41.6	61.5	72.6	79.6
Ca_26725	-0.0138	0.0104	0.0172	uncharacterized protein LOC100798035 [Glycine max]		4.8	4.4	0.0	7.8	11.2	9.9
Ca_17580	-0.0126	0.0076	0.0147	uncharacterized protein LOC100798851 [Glycine max]		3.3	3.9	0.0	5.3	15.0	7.5
Ca_17021	-0.0149	0.0080	0.0169	uncharacterized protein LOC100800312 [Glycine max]		20.5	24.3	17.7	30.0	45.7	46.1
Ca_02287	-0.0042	0.0140	0.0146	uncharacterized protein LOC100800379 isoform X1 [Glycine max]		127.6	41.2	35.1	63.8	71.2	78.9
Ca_01633	-0.0023	0.0134	0.0136	uncharacterized protein LOC100800409 isoform X7 [Glycine max]		94.2	52.1	46.8	60.0	75.3	60.2
Ca_10920	-0.0118	0.0092	0.0149	uncharacterized protein LOC100801654 [Glycine max]		0.0	0.0	0.0	5.6	7.1	0.0
Ca_17357	-0.0163	0.0076	0.0179	uncharacterized protein LOC100803657 isoform X1 [Glycine max]		38.6	47.1	39.1	69.4	113.8	91.2
Ca_18461	-0.0074	0.0110	0.0132	uncharacterized protein LOC100803817 [Glycine max]		113.2	67.1	68.2	109.0	111.0	89.8
Ca_12242	-0.0136	0.0059	0.0148	uncharacterized protein LOC100805509 isoform X2 [Glycine max]	GO:0006508 GO:0008234	8.9	13.4	12.2	18.1	27.2	20.7
Ca_26704	-0.0139	0.0083	0.0162	uncharacterized protein LOC100807255 isoform X9 [Glycine max]		4.8	2.6	6.4	11.4	10.4	12.1
Ca_15136	-0.0082	0.0101	0.0130	uncharacterized protein LOC100807554 isoform X1 [Glycine max]		209.9	159.0	116.1	157.2	225.2	241.3
Ca_26589	-0.0135	0.0038	0.0140	uncharacterized protein LOC100808366 isoform X3 [Glycine max]	GO:0003676 GO:0008270	144.6	141.3	137.4	207.8	276.4	217.9
Ca_08580	-0.0029	0.0160	0.0162	uncharacterized protein LOC100809482 isoform X5 [Glycine max]		159.6	70.7	65.3	94.8	115.6	86.7
Ca_10122	-0.0050	0.0125	0.0134	uncharacterized protein LOC100811297 isoform X3 [Glycine max]		90.0	55.1	39.4	90.3	56.9	72.7
Ca_30247	-0.0104	0.0096	0.0142	uncharacterized protein LOC100812827 [Glycine max]		0.7	0.0	0.0	0.0	3.8	2.4
Ca_06202	-0.0123	0.0049	0.0133	uncharacterized protein LOC100816817 isoform X3 [Glycine max]		63.3	96.4	72.7	94.8	129.0	152.2
Ca_18714	-0.0032	0.0135	0.0139	uncharacterized protein LOC100817734 [Glycine max]		259.3	201.8	156.1	231.5	189.7	221.5
Ca_04023	-0.0159	0.0097	0.0186	uncharacterized protein LOC100817991 [Glycine max]		2.5	0.0	0.0	5.7	4.7	7.0
Ca_18455	-0.0090	0.0119	0.0150	uncharacterized protein LOC100819317 isoform X1 [Glycine max]		159.0	103.3	139.4	167.3	135.3	172.9
Ca_04380	-0.0106	0.0136	0.0173	uncharacterized protein LOC100820019 isoform X4 [Glycine max]		7.0	1.7	3.5	7.8	6.9	8.0
Ca_12405	-0.0134	0.0023	0.0136	uncharacterized protein LOC100820019 isoform X4 [Glycine max]		14.5	16.1	23.8	34.8	33.5	31.4
Ca_19259	-0.0125	0.0058	0.0137	uncharacterized protein LOC100820019 isoform X4 [Glycine max]		8.7	4.7	12.4	15.6	30.5	11.5
Ca_10449	-0.0123	0.0102	0.0160	uncharacterized protein LOC100820019 isoform X5 [Glycine max]		4.1	6.0	3.1	7.0	15.9	12.2
Ca_08103	-0.0164	0.0112	0.0198	uncharacterized protein LOC100820117 [Glycine max]		0.0	0.0	0.0	3.5	7.4	5.7
Ca_09150	-0.0147	0.0066	0.0161	uncharacterized protein LOC100820117 [Glycine max]		6.3	12.8	14.4	21.3	39.2	25.1
Ca_18134	-0.0128	0.0060	0.0141	uncharacterized protein LOC100820117 [Glycine max]		0.0	0.0	0.0	0.0	4.5	4.9
Ca_24867	-0.0121	0.0066	0.0138	uncharacterized protein LOC100820117 [Glycine max]		0.0	0.0	0.0	0.0	3.5	5.1

Ca_27510	-0.0104	0.0127	0.0164	uncharacterized protein LOC100820117 [Glycine max]		5.4	0.0	0.0	5.6	8.7	6.1
Ca_25569	-0.0134	0.0064	0.0149	uncharacterized protein LOC102659949 [Glycine max]		2.1	2.8	0.0	6.3	6.8	9.7
Ca_10077	-0.0039	0.0135	0.0140	uncharacterized protein LOC102660937 [Glycine max]		188.2	80.6	105.9	140.5	113.1	135.9
Ca_27652	-0.0128	0.0056	0.0140	uncharacterized protein LOC102660961 [Glycine max]	GO:0003676 GO:0008270	12.4	15.7	12.0	19.5	46.6	32.9
Ca_12928	-0.0127	0.0096	0.0160	uncharacterized protein LOC102661713 [Glycine max]		3.1	0.0	0.0	6.3	14.4	5.6
Ca_27525	-0.0145	0.0067	0.0159	uncharacterized protein LOC102662165 [Glycine max]		14.8	29.0	27.7	36.8	42.8	35.0
Ca_09157	-0.0153	0.0050	0.0161	uncharacterized protein LOC102662333 [Glycine max]		58.0	57.0	54.2	83.4	105.4	104.8
Ca_22933	-0.0137	0.0081	0.0159	uncharacterized protein LOC102662399 [Glycine max]	GO:0003676 GO:0008270	2.7	6.0	4.0	6.7	11.5	8.3
Ca_01260	-0.0134	0.0048	0.0142	uncharacterized protein LOC102662869 [Glycine max]	GO:0003676 GO:0008270	21.2	12.7	13.2	27.8	23.7	34.1
Ca_10824	-0.0119	0.0074	0.0140	uncharacterized protein LOC102663104 [Glycine max]		0.0	0.0	0.0	0.0	2.8	5.9
Ca_17167	0.0007	0.0138	0.0138	uncharacterized protein LOC102663354 isoform X6 [Glycine max]		101.5	23.9	24.9	36.1	32.5	31.1
Ca_30034	-0.0130	0.0141	0.0192	uncharacterized protein LOC102663453 [Glycine max]		11.4	13.0	5.5	16.9	18.4	17.7
Ca_27417	-0.0126	0.0038	0.0132	uncharacterized protein LOC102663497 [Glycine max]		4.6	8.3	8.3	11.5	13.2	18.8
Ca_02769	-0.0131	0.0022	0.0132	uncharacterized protein LOC102663500 [Glycine max]		2.9	10.6	7.1	12.1	21.7	11.3
Ca_26317	-0.0122	0.0068	0.0140	uncharacterized protein LOC102663500 [Glycine max]		0.0	0.0	0.0	0.0	21.2	40.5
Ca_19397	-0.0093	0.0135	0.0164	uncharacterized protein LOC102664163 isoform X5 [Glycine max]		135.2	63.0	82.8	148.1	103.7	151.7
Ca_27940	-0.0130	0.0016	0.0130	uncharacterized protein LOC102664163 isoform X7 [Glycine max]		40.4	41.1	38.4	63.5	76.5	77.2
Ca_05624	-0.0168	0.0100	0.0196	uncharacterized protein LOC102664401 [Glycine max]		0.0	0.0	0.0	2.3	5.8	6.8
Ca_18113	-0.0027	0.0134	0.0136	uncharacterized protein LOC102664406 [Glycine max]		42.0	13.5	10.3	17.9	19.3	28.2
Ca_08932	-0.0081	0.0108	0.0134	uncharacterized protein LOC102664583 [Glycine max]		103.2	74.5	66.1	90.5	95.0	102.8
Ca_10389	-0.0140	0.0072	0.0158	uncharacterized protein LOC102664627 isoform X2 [Glycine max]		11.3	13.7	15.7	18.9	35.7	25.6
Ca_12982	-0.0115	0.0064	0.0132	uncharacterized protein LOC102664679 isoform X1 [Glycine max]		0.0	0.0	0.0	0.0	1.5	1.7
Ca_26102	-0.0130	0.0069	0.0147	uncharacterized protein LOC102664679 isoform X1 [Glycine max]		39.5	20.1	58.1	108.0	57.4	69.3
Ca_29910	-0.0115	0.0076	0.0138	uncharacterized protein LOC102664679 isoform X1 [Glycine max]		0.0	0.0	0.0	8.6	0.0	2.9
Ca_01675	-0.0136	0.0081	0.0158	uncharacterized protein LOC102664679 isoform X3 [Glycine max]		3.8	6.1	0.0	9.3	7.7	11.8
Ca_28042	-0.0114	0.0066	0.0132	uncharacterized protein LOC102664679 isoform X3 [Glycine max]		0.0	0.0	0.0	0.0	1.7	3.0
Ca_11010	-0.0147	0.0097	0.0176	uncharacterized protein LOC102664679 isoform X4 [Glycine max]		1.9	0.0	0.0	4.8	3.2	6.4
Ca_17955	-0.0161	0.0058	0.0171	uncharacterized protein LOC102664679 isoform X4 [Glycine max]		6.6	10.7	13.3	22.8	26.6	24.5
Ca_20751	-0.0117	0.0094	0.0150	uncharacterized protein LOC102664679 isoform X4 [Glycine max]		0.0	0.0	0.0	8.3	4.2	0.0
Ca_27375	-0.0054	0.0121	0.0132	uncharacterized protein LOC102664679 isoform X4 [Glycine max]		2.5	0.0	0.0	7.4	0.0	0.0
Ca_26896	-0.0053	0.0136	0.0145	uncharacterized protein LOC102664992 [Glycine max]		47.3	28.6	26.8	38.1	42.2	40.5
Ca_01302	-0.0004	0.0132	0.0132	uncharacterized protein LOC102665201 isoform X3 [Glycine max]		143.5	65.1	71.8	73.1	101.6	75.7
Ca_19302	-0.0069	0.0113	0.0132	uncharacterized protein LOC102666946 isoform X4 [Glycine max]		325.9	221.0	299.6	363.6	291.0	324.8
Ca_26989	-0.0137	0.0074	0.0156	uncharacterized protein LOC102667494 [Glycine max]		0.0	3.0	0.0	6.7	11.8	6.6
Ca_27234	-0.0133	0.0100	0.0167	uncharacterized protein LOC102667494 [Glycine max]		0.4	0.0	0.0	6.2	2.5	7.9
Ca_19945	-0.0120	0.0081	0.0145	uncharacterized protein LOC102667723 [Glycine max]		3.5	3.1	5.7	9.4	10.6	8.5
Ca_17635	-0.0139	0.0011	0.0140	uncharacterized protein LOC102668358 [Glycine max]	GO:0003676 GO:0008270	40.2	55.8	48.3	68.0	70.9	84.8
Ca_19676	-0.0121	0.0075	0.0142	uncharacterized protein LOC102668569 [Glycine max]		0.0	0.0	0.0	0.0	2.6	5.4
Ca_01560	-0.0116	0.0118	0.0166	uncharacterized protein LOC102669390 [Glycine max]		6.2	6.1	0.0	11.1	9.9	6.9
Ca_17679	-0.0140	0.0059	0.0152	uncharacterized protein LOC102669914 [Glycine max]		22.1	23.6	21.5	37.6	43.1	45.0
Ca_00991	-0.0014	0.0130	0.0130	uncharacterized protein LOC102669949 isoform X1 [Glycine max]		103.7	52.2	39.6	53.2	68.6	64.6
Ca_25512	-0.0121	0.0051	0.0132	uncharacterized protein LOC102670184 [Glycine max]	GO:0003676 GO:0008270	0.0	0.0	5.0	10.5	7.1	4.1
Ca_27879	-0.0138	0.0075	0.0157	uncharacterized protein LOC102670333 [Glycine max]		0.8	0.8	3.6	5.5	23.9	9.1
Ca_13003	-0.0152	0.0101	0.0183	uncharacterized protein LOC102670505 [Glycine max]		2.7	2.6	1.2	8.9	5.2	7.6
Ca_28408	-0.0126	0.0070	0.0144	Undecaprenyl-diphosphatase n=11 Tax=Enterobacteriaceae RepID=D4GN59_PANAM	GO:0016020 GO:0016311 GO:0050380	2.2	5.2	0.0	12.8	8.6	10.9
Ca_00045	-0.0181	0.0112	0.0213	Unknown protein		2.6	0.0	0.0	7.2	6.8	10.5

Ca_01107	-0.0148	0.0071	0.0164	Unknown protein		33.4	28.6	22.1	51.3	54.3	47.7
Ca_01178	-0.0162	0.0099	0.0190	Unknown protein		2.4	0.0	0.0	4.4	7.1	9.4
Ca_01229	-0.0101	0.0105	0.0146	Unknown protein		1.9	0.0	0.0	0.0	2.9	3.9
Ca_01442	-0.0155	0.0096	0.0182	Unknown protein		1.4	1.9	0.0	12.3	7.7	10.4
Ca_01485	-0.0098	0.0095	0.0136	Unknown protein		2.6	0.0	0.0	5.2	5.2	0.0
Ca_01548	-0.0096	0.0095	0.0135	Unknown protein		0.0	0.0	0.0	1.3	0.0	1.6
Ca_01710	-0.0128	0.0055	0.0139	Unknown protein		5.2	4.4	3.0	11.4	7.1	11.6
Ca_02745	-0.0135	0.0047	0.0143	Unknown protein		3.4	10.0	11.5	22.0	25.9	26.1
Ca_02747	-0.0129	0.0017	0.0130	unknown protein		5.4	5.2	6.2	8.3	13.8	12.7
Ca_03646	-0.0130	0.0089	0.0158	Unknown protein		2.4	0.0	2.7	7.8	4.2	7.7
Ca_03901	-0.0141	0.0051	0.0150	Unknown protein		0.0	0.0	6.4	8.9	12.4	8.4
Ca_04064	-0.0164	0.0064	0.0176	Unknown protein		2.7	2.7	5.5	14.7	15.5	12.7
Ca_04143	-0.0144	0.0039	0.0150	Unknown protein		2.9	4.7	6.4	9.6	9.8	12.6
Ca_04183	-0.0130	0.0068	0.0147	Unknown protein		0.0	0.0	0.0	0.0	1.6	2.5
Ca_04279	-0.0105	0.0083	0.0134	Unknown protein		0.0	0.0	0.0	8.1	2.7	0.0
Ca_04410	-0.0162	0.0089	0.0185	Unknown protein		3.5	2.6	3.4	11.2	10.0	9.3
Ca_04874	-0.0141	0.0082	0.0163	Unknown protein		2.6	0.0	3.8	11.9	8.5	8.8
Ca_05154	-0.0134	0.0101	0.0168	Unknown protein	GO:0003676 GO:0008270	2.3	0.0	0.0	6.7	4.4	5.2
Ca_05367	-0.0043	0.0126	0.0133	Unknown protein		332.4	148.8	88.2	186.5	145.9	316.7
Ca_05502	-0.0109	0.0086	0.0139	Unknown protein	GO:0003676 GO:0008270	4.6	0.0	0.0	10.0	16.0	7.8
Ca_05810	-0.0134	0.0060	0.0147	Unknown protein		2.8	0.0	5.2	9.9	6.9	7.6
Ca_06244	-0.0135	0.0033	0.0139	Unknown protein		9.7	17.5	15.6	27.4	27.4	35.1
Ca_06248	-0.0117	0.0102	0.0156	Unknown protein		0.6	0.0	0.0	3.0	2.5	0.0
Ca_07557	-0.0138	0.0055	0.0149	Unknown protein		0.0	0.9	0.0	3.8	2.6	4.1
Ca_08059	-0.0139	0.0050	0.0147	Unknown protein		8.7	9.8	8.5	14.9	15.2	14.5
Ca_08120	-0.0113	0.0084	0.0141	Unknown protein		4.6	0.0	5.4	8.6	15.5	5.5
Ca_08237	-0.0166	0.0123	0.0207	Unknown protein		1.1	0.0	0.0	8.2	7.5	5.9
Ca_08400	-0.0142	0.0064	0.0156	unknown protein		8.4	17.1	9.3	20.4	37.4	20.0
Ca_09673	-0.0125	0.0128	0.0179	Unknown protein		6.3	0.0	0.0	6.2	6.6	8.0
Ca_09980	-0.0130	0.0038	0.0136	Unknown protein		1.5	6.8	16.7	16.5	32.3	15.1
Ca_10566	-0.0135	0.0045	0.0142	unknown protein		27.2	34.1	44.4	48.0	70.3	64.5
Ca_10819	-0.0116	0.0142	0.0183	Unknown protein		7.5	0.0	2.7	7.8	11.9	11.0
Ca_10913	-0.0093	0.0105	0.0140	Unknown protein		1.4	3.0	0.0	8.5	1.7	6.9
Ca_11279	-0.0125	0.0048	0.0134	Unknown protein		0.0	0.0	0.0	0.0	3.5	6.0
Ca_11367	-0.0125	0.0092	0.0156	Unknown protein		0.0	0.0	0.0	0.0	4.6	4.5
Ca_11609	-0.0122	0.0075	0.0143	Unknown protein		2.0	0.0	0.0	9.0	0.0	7.4
Ca_11632	-0.0125	0.0085	0.0151	Unknown protein		3.2	1.7	0.0	4.9	3.9	6.8
Ca_11667	-0.0132	0.0022	0.0134	Unknown protein		3.2	8.0	13.7	11.5	22.5	20.0
Ca_12035	-0.0111	0.0077	0.0135	Unknown protein		3.5	4.7	0.0	6.2	5.2	4.8
Ca_12038	-0.0118	0.0085	0.0146	Unknown protein		0.4	0.0	0.0	5.9	6.3	0.0
Ca_12111	-0.0143	0.0081	0.0164	Unknown protein		20.7	16.7	11.2	28.0	29.5	33.8
Ca_12193	-0.0138	0.0012	0.0138	Unknown protein		15.4	28.9	27.6	41.1	41.7	41.7
Ca_12366	-0.0137	0.0074	0.0155	Unknown protein		13.1	12.5	9.7	19.5	23.4	24.5
Ca_12617	-0.0149	0.0072	0.0165	Unknown protein		0.0	1.3	0.0	8.8	8.7	14.8
Ca_12733	-0.0036	0.0163	0.0167	Unknown protein		109.4	29.2	25.5	62.4	55.7	37.4
Ca_12772	-0.0134	0.0049	0.0143	Unknown protein		4.3	2.5	3.4	7.3	8.8	9.6
Ca_12843	-0.0125	0.0112	0.0167	Unknown protein		6.6	0.0	3.7	8.5	6.2	9.7
Ca_13041	-0.0128	0.0101	0.0163	Unknown protein		5.5	3.5	0.0	10.3	4.7	12.1
Ca_13749	-0.0091	0.0121	0.0152	Unknown protein		3.0	0.0	0.0	8.6	7.2	0.0
Ca_14666	-0.0070	0.0157	0.0172	Unknown protein		106.0	82.4	68.6	88.9	98.1	112.1
Ca_15930	-0.0172	0.0112	0.0205	Unknown protein		0.0	0.0	0.0	4.6	3.7	2.3
Ca_16471	-0.0137	0.0011	0.0138	unknown protein		26.0	25.8	35.2	48.0	55.9	54.7
Ca_16571	-0.0056	0.0143	0.0154	unknown protein		15.5	0.0	1.8	6.6	7.7	10.5
Ca_16623	-0.0134	0.0094	0.0163	Unknown protein		0.0	0.0	0.0	4.2	9.1	4.0
Ca_16845	-0.0150	0.0061	0.0162	Unknown protein		5.0	5.4	5.2	11.6	22.6	15.8
Ca_17057	-0.0121	0.0073	0.0141	Unknown protein		4.0	4.4	0.0	6.5	6.5	6.2
Ca_17485	-0.0120	0.0051	0.0130	Unknown protein		1.0	2.4	5.1	11.3	4.0	10.3
Ca_18081	-0.0134	0.0024	0.0136	Unknown protein		3.1	6.7	5.6	13.5	8.7	11.2
Ca_18168	-0.0157	0.0115	0.0195	Unknown protein		0.0	0.0	0.0	8.6	3.3	3.0
Ca_18509	-0.0155	0.0123	0.0198	Unknown protein		1.9	3.1	0.0	4.7	6.6	6.8
Ca_19786	-0.0150	0.0090	0.0175	Unknown protein		0.0	0.0	0.0	7.1	6.3	4.4
Ca_20217	-0.0172	0.0087	0.0193	Unknown protein		4.9	5.2	5.3	20.9	14.4	15.2
Ca_20415	-0.0134	0.0080	0.0156	Unknown protein		0.6	0.0	0.0	2.8	1.6	3.1
Ca_21304	-0.0172	0.0138	0.0221	Unknown protein		2.5	0.0	0.0	8.6	6.6	7.1
Ca_21375	-0.0138	0.0085	0.0162	Unknown protein		7.0	2.3	6.3	20.4	9.2	11.6
Ca_22849	-0.0143	0.0052	0.0152	unknown protein		2.0	3.6	9.9	16.8	20.9	19.4
Ca_23192	-0.0144	0.0089	0.0169	Unknown protein		0.0	0.0	0.0	8.2	1.6	4.9
Ca_23828	-0.0122	0.0050	0.0132	Unknown protein		1.8	2.5	8.4	28.9	6.1	15.3
Ca_23888	-0.0105	0.0100	0.0145	Unknown protein		12.3	12.5	9.6	17.5	14.1	21.4
Ca_23924	-0.0016	0.0133	0.0134	Unknown protein		161.4	14.2	12.8	23.7	36.0	35.0
Ca_23944	-0.0160	0.0098	0.0188	Unknown protein		0.0	0.0	0.0	4.9	7.3	6.9
Ca_23949	-0.0139	0.0098	0.0170	Unknown protein		0.0	1.8	0.0	7.2	3.3	5.8
Ca_24278	-0.0132	0.0055	0.0143	Unknown protein		2.3	0.0	2.8	4.6	6.3	4.2
Ca_24604	-0.0122	0.0049	0.0131	Unknown protein		7.4	7.8	7.4	11.5	34.2	21.6
Ca_24648	-0.0144	0.0061	0.0157	Unknown protein		1.3	1.7	5.7	12.4	6.4	9.6
Ca_24883	-0.0124	0.0084	0.0150	Unknown protein		11.2	23.3	17.5	36.1	19.8	31.4
Ca_24904	-0.0136	0.0089	0.0162	Unknown protein		0.0	0.0	0.0	2.7	16.2	3.1
Ca_24958	-0.0109	0.0108	0.0153	Unknown protein		4.7	4.1	3.8	12.1	6.1	9.6
Ca_25100	-0.0126	0.0052	0.0136	Unknown protein		4.6	9.1	11.4	11.4	21.6	15.9
Ca_25242	-0.0125	0.0101	0.0161	Unknown protein		3.4	3.8	0.0	5.3	10.5	8.0
Ca_25282	-0.0126	0.0097	0.0159	Unknown protein		0.0	0.0	0.0	1.8	2.1	0.0
Ca_25395	-0.0107	0.0100	0.0147	Unknown protein		5.4	4.4	0.0	7.5	6.6	8.3
Ca_25498	-0.0121	0.0079	0.0145	Unknown protein		0.0	0.0	0.0	0.0	2.5	3.4
Ca_25568	-0.0155	0.0096	0.0183	Unknown protein		0.0	0.0	0.0	7.2	3.2	4.7
Ca_25603	-0.0130	0.0035	0.0135	Unknown protein		5.6	4.9	8.0	9.5	13.7	16.0
Ca_25695	-0.0131	0.0066	0.0147	Unknown protein		7.6	4.9	4.8	14.0	18.4	8.2
Ca_25741	-0.0141	0.0107	0.0177	Unknown protein		5.0	0.0	2.2	9.7	5.9	8.2
Ca_25757	-0.0137	0.0092	0.0165	Unknown protein		0.0	0.0	0.0	10.3	11.7	4.3
Ca_25976	-0.0130	0.0104	0.0167	Unknown protein		5.7	6.0	0.0	14.8	15.7	9.2
Ca_25995	-0.0133	0.0024	0.0135	Unknown protein		14.5	18.7	27.1	34.9	34.0	34.9
Ca_26277	-0.0125	0.0067	0.0142	Unknown protein		0.0	0.0	0.0	0.0	2.5	6.8
Ca_26310	-0.0148	0.0096	0.0177	Unknown protein		0.0	0.0	0.0	3.6	4.1	4.4
Ca_26348	-0.0124	0.0056	0.0136	Unknown protein		0.4	3.1	0.0	5.1	3.0	5.3

Ca_26588	-0.0133	0.0072	0.0151	Unknown protein		0.0	0.0	0.0	0.0	3.2	1.7
Ca_26611	-0.0135	0.0067	0.0151	Unknown protein		0.0	0.0	3.9	5.0	7.2	6.4
Ca_26700	-0.0134	0.0072	0.0152	Unknown protein		4.6	9.5	16.9	24.7	22.3	24.3
Ca_26719	-0.0149	0.0056	0.0159	Unknown protein		6.6	5.9	7.1	10.9	16.5	13.9
Ca_26833	-0.0182	0.0127	0.0222	Unknown protein		1.1	0.0	0.0	6.4	6.2	9.3
Ca_27111	-0.0132	0.0081	0.0155	Unknown protein		0.0	0.0	0.0	0.0	8.2	5.3
Ca_27113	-0.0118	0.0060	0.0132	Unknown protein		0.0	0.0	0.0	8.0	0.0	5.8
Ca_27201	-0.0111	0.0080	0.0137	Unknown protein		6.9	4.8	0.0	5.7	9.4	10.1
Ca_27349	-0.0129	0.0040	0.0135	Unknown protein		13.3	24.9	23.3	33.3	52.3	89.9
Ca_27446	-0.0099	0.0127	0.0161	Unknown protein		2.2	0.0	0.0	5.1	5.0	0.0
Ca_27500	-0.0149	0.0105	0.0182	Unknown protein		2.7	0.0	0.0	10.4	4.0	5.4
Ca_27591	-0.0130	0.0070	0.0147	Unknown protein		5.4	7.2	5.3	13.0	17.0	11.6
Ca_27818	-0.0145	0.0047	0.0153	Unknown protein		8.0	5.6	11.7	14.3	19.3	13.5
Ca_27824	-0.0114	0.0065	0.0131	Unknown protein		2.1	7.3	6.7	16.2	8.7	9.0
Ca_27839	-0.0081	0.0102	0.0130	Unknown protein		173.4	71.4	84.5	86.0	160.4	230.9
Ca_27898	-0.0133	0.0111	0.0173	Unknown protein		0.0	1.7	0.0	5.5	4.0	8.3
Ca_28050	-0.0136	0.0054	0.0146	Unknown protein		5.7	4.8	3.1	6.6	13.1	12.0
Ca_28148	-0.0111	0.0090	0.0142	Unknown protein		0.3	0.0	0.0	12.8	7.1	0.0
Ca_28208	-0.0125	0.0056	0.0136	Unknown protein		9.7	5.4	7.1	14.0	13.1	12.5
Ca_28285	-0.0129	0.0087	0.0156	Unknown protein		7.3	0.0	0.0	5.5	8.1	13.1
Ca_28426	-0.0123	0.0085	0.0149	Unknown protein		2.2	3.2	0.6	3.5	6.0	7.0
Ca_28772	-0.0159	0.0095	0.0185	Unknown protein		0.0	0.0	0.0	4.0	3.0	5.9
Ca_28953	-0.0123	0.0085	0.0150	Unknown protein		2.9	0.0	0.9	10.1	4.0	8.2
Ca_25515	-0.0029	0.0135	0.0138	UPF0392 protein RCOM_0530710-like [Glycine max]		62.4	41.0	37.0	44.7	46.6	53.9
Ca_16859	-0.0129	0.0028	0.0132	UPF0481 protein At3g47200-like [Glycine max]		26.1	35.3	30.1	33.4	81.7	92.1
Ca_27413	-0.0034	0.0128	0.0132	UPF0481 protein At3g47200-like [Glycine max]		212.8	84.3	40.7	126.1	89.3	122.3
Ca_29239	-0.0113	0.0071	0.0134	uracil dna glycosylase	GO:0004844 GO:0006281 GO:0006284	3.0	4.0	0.0	7.9	4.4	12.8
Ca_05535	-0.0156	0.0059	0.0167	vacuolar amino acid transporter 1-like [Glycine max]		25.1	31.8	36.8	52.7	70.1	72.5
Ca_12127	-0.0033	0.0140	0.0144	vacuolar cation/proton exchanger 5-like isoform X4 [Glycine max]		80.6	16.0	20.1	28.0	41.5	37.5
Ca_00518	-0.0094	0.0110	0.0145	vacuolar fusion MON1-like protein		1411.7	751.1	1245.9	2462.2	1147.8	1487.3
Ca_05963	-0.0134	0.0069	0.0151	vacuolar-processing enzyme-like [Glycine max]	GO:0004197 GO:0006508	9.4	13.2	18.2	21.2	50.5	27.2
Ca_29127	-0.0126	0.0051	0.0136	Virulence factor VirK n=3 Tax=Enterobacter aerogenes RepID=L8BEJ8_ENTAE		0.0	0.0	0.0	0.0	4.2	7.1
Ca_22668	-0.0136	0.0058	0.0148	VQ motif protein		12.3	24.2	11.4	24.1	46.0	31.0
Ca_02371	-0.0118	0.0095	0.0151	WD-40 repeat family protein	GO:0005515	1.4	0.0	3.0	8.4	7.3	5.4
Ca_01532	-0.0132	0.0105	0.0168	WD-repeat cell cycle regulatory protein [Glycine max]	GO:0005515	5.5	8.9	4.6	15.3	12.2	12.9
Ca_02864	-0.0159	0.0082	0.0179	WPP domain interacting protein 2		6.0	8.0	6.4	13.2	15.3	14.1
Ca_22014	-0.0141	0.0038	0.0146	WRKY family transcription factor	GO:0003700 GO:0006355 GO:0043565	140.2	151.8	292.7	420.3	224.4	439.9
Ca_06587	-0.0106	0.0124	0.0163	WRKY family transcription factor family protein	GO:0003700 GO:0006355 GO:0043565	816.9	592.4	305.6	785.3	1742.1	1241.1
Ca_22117	-0.0145	0.0125	0.0191	YABBY transcription factor		2.1	1.8	0.0	4.4	5.1	6.1
Ca_14164	-0.0067	0.0147	0.0162	zinc finger CCCH domain-containing protein 38-like isoform X4 [Glycine max]	GO:0046872	852.9	671.6	584.2	772.0	963.7	759.4
Ca_27181	-0.0131	0.0096	0.0163	zinc knuckle (CCHC-type) family protein	GO:0003676 GO:0008270	4.9	5.7	0.0	12.6	10.8	9.1
Ca_16963	-0.0139	0.0110	0.0177	Zinc-binding dehydrogenase family protein	GO:0005975 GO:0008184 GO:0008270 GO:0016491 GO:0016747 GO:0055114	107.2	119.4	98.4	132.8	176.8	161.6
Ca_08115	-0.0053	0.0120	0.0131	zinc-finger protein 2	GO:0003676 GO:0046872	54.6	55.3	17.7	48.1	57.3	44.6

High nodulation associated

Ca_15361	0.0148	-0.0037	0.0153	26S proteasome non-ATPase regulatory subunit 7 homolog A-like [Glycine max]	GO:0005515	867.6	679.0	773.5	560.6	569.7	544.0
Ca_17108	0.0146	-0.0030	0.0149	26S proteasome non-ATPase regulatory subunit 8 homolog A-like [Glycine max]	GO:0005838 GO:0006508	789.5	701.7	506.4	431.5	464.6	414.3
Ca_08925	0.0139	0.0014	0.0139	26S proteasome regulatory subunit 4 homolog A [Glycine max]	GO:0000166 GO:0005524 GO:0005737 GO:0016787 GO:0017111 GO:0030163	2184.9	1738.8	1573.6	1214.3	1113.1	1139.0
Ca_22207	0.0028	-0.0136	0.0139	2Fe-2S ferredoxin-like superfamily protein	GO:0009055 GO:0051536	92.5	180.3	206.7	149.0	110.7	109.1
Ca_14179	0.0141	-0.0045	0.0148	2-oxoglutarate (2OG) and Fe(II)-dependent oxygenase superfamily protein	GO:0016491 GO:0016706 GO:0055114	238.4	59.8	80.5	4.4	14.2	68.0
Ca_22636	0.0154	-0.0017	0.0155	2-oxoglutarate (2OG) and Fe(II)-dependent oxygenase superfamily protein	GO:0016491 GO:0016706 GO:0055114	305.4	88.2	75.7	21.2	20.6	27.8
Ca_21684	0.0139	-0.0004	0.0139	3-ketoacyl-CoA synthase 11	GO:0003824 GO:0006633 GO:0008152 GO:0008610 GO:0016020 GO:0016747	762.6	501.1	485.8	443.9	382.7	372.6
Ca_22437	0.0140	-0.0007	0.0140	4-coumarate:CoA ligase 2	GO:0003824 GO:0008152	251.7	160.2	137.0	120.8	117.7	100.9
Ca_08684	0.0086	-0.0129	0.0155	5'-3' exoribonuclease 3	GO:0003676 GO:0004527 GO:0004534 GO:0005622 GO:0005634 GO:0006139 GO:0008270	31.5	84.8	52.8	14.9	28.4	21.5
Ca_12143	0.0059	-0.0117	0.0131	8-amino-7-oxononanoate synthase-like protein	GO:0003824 GO:0008152 GO:0009058 GO:0016740 GO:0030170	78.9	90.8	73.4	75.3	42.1	57.9
Ca_14951	0.0138	-0.0013	0.0138	ABC transporter family protein	GO:0005524 GO:0016887	297.4	121.8	123.5	27.0	59.5	83.1
Ca_26287	0.0130	-0.0056	0.0141	acetylornithine aminotransferase	GO:0003824 GO:0006525 GO:0008483 GO:0030170	709.8	672.4	613.1	532.3	540.7	510.4
Ca_09219	0.0098	-0.0114	0.0151	acid phosphatase 1-like [Glycine max]	GO:0003993	0.0	2.7	34.9	0.0	0.0	0.0
Ca_13727	0.0092	-0.0102	0.0137	Acid phosphatase/vanadium-dependent haloperoxidase-related protein		156.6	115.7	146.7	126.0	112.7	119.5
Ca_10271	0.0077	-0.0125	0.0147	Acyl-[acyl-carrier-protein] desaturase n=2 Tax=Solanum RepID=K4C635_SOLLC	GO:0006631 GO:0016491 GO:0045300 GO:0055114	793.4	1193.0	959.1	740.1	888.6	464.9
Ca_00586	0.0190	-0.0150	0.0242	Adaptin ear-binding coat-associated protein 1 NECAP-1	GO:0006897 GO:0016020	122.7	231.6	223.1	8.5	12.7	17.5
Ca_08133	0.0097	-0.0102	0.0140	Adenine nucleotide alpha hydrolases-like superfamily protein	GO:0006950	5100.5	5124.6	4927.1	3779.2	3716.6	2673.1

Ca_09873	0.0121	-0.0071	0.0140	Adenine nucleotide alpha hydrolases-like superfamily protein	GO:0006950	576.1	660.2	520.9	409.7	448.2	361.7
Ca_14758	0.0139	-0.0077	0.0159	adenine phosphoribosyltransferase 5	GO:0003999 GO:0005737 GO:0006168 GO:0009116	1018.9	2016.4	872.1	544.0	643.8	525.7
Ca_02268	0.0003	-0.0137	0.0137	ADP-ribosylation factor 1	GO:0005525 GO:0005622 GO:0006886 GO:0007264 GO:0015031	181.5	263.9	310.4	248.1	193.3	254.3
Ca_04947	0.0111	-0.0087	0.0142	ADP-ribosylation factor 1	GO:0005525 GO:0005622 GO:0006886 GO:0007264 GO:0015031	4245.6	4084.1	4993.6	3854.7	3454.1	3410.5
Ca_07157	0.0146	-0.0015	0.0146	ADP-ribosylation factor 1	GO:0005525 GO:0005622 GO:0006886 GO:0007264 GO:0015031	812.7	774.3	410.9	325.1	342.1	380.9
Ca_08401	0.0049	-0.0132	0.0141	ADP-ribosylation factor 1	GO:0005525 GO:0005622 GO:0006886 GO:0007264 GO:0015031	182.1	347.9	246.5	191.2	132.9	141.7
Ca_22775	0.0132	-0.0032	0.0136	ADP-ribosylation factor 1	GO:0005525 GO:0005622 GO:0006886 GO:0007264	527.7	351.0	362.1	357.6	259.6	180.1
Ca_26280	0.0082	-0.0122	0.0147	agenet domain-containing protein		0.0	9.9	8.6	0.0	0.0	5.5
Ca_13967	0.0080	-0.0125	0.0149	alcohol dehydrogenase 1	GO:0008270 GO:0016491 GO:0055114	1542.5	2143.4	2310.8	1831.8	1691.7	1169.5
Ca_06117	0.0112	-0.0168	0.0202	aldo/keto reductase family oxidoreductase		10.6	48.0	36.5	9.2	9.5	12.7
Ca_16673	0.0158	-0.0026	0.0160	alpha N-terminal protein methyltransferase 1-like isoform X1 [Glycine max]	GO:0008168	413.8	331.0	306.0	193.9	236.3	187.8
Ca_26818	0.0122	-0.0047	0.0131	alpha-soluble NSF attachment protein 2	GO:0005515 GO:0006886	488.1	546.8	427.3	340.1	413.2	343.0
Ca_09486	0.0112	-0.0146	0.0184	Ankyrin repeat family protein	GO:0005515	0.0	6.5	8.8	0.0	0.9	0.0
Ca_04525	0.0141	-0.0090	0.0168	anthranilate synthase 2	GO:0000162 GO:0004049 GO:0009058 GO:0016833	372.6	406.0	384.5	283.0	290.4	252.5
Ca_10730	0.0115	-0.0108	0.0158	anthranilate synthase 2	GO:0000162 GO:0004049 GO:0009058 GO:0016833	917.4	1497.6	1244.6	883.5	1013.1	666.2
Ca_19950	0.0136	-0.0098	0.0168	anticodon-binding domain protein		438.2	316.7	419.1	287.0	262.5	202.2
Ca_15587	0.0107	-0.0077	0.0132	arabinogalactan peptide 16-like [Glycine max]		8963.7	12483.2	11414.4	9121.8	7957.5	6130.8
Ca_21227	0.0039	-0.0148	0.0153	arginine/serine-rich-splicing factor RSP41-like isoform X1 [Glycine max]	GO:0000166 GO:0003676	27.1	72.9	73.0	45.4	34.0	50.4
Ca_12970	0.0132	-0.0049	0.0141	asterix-like protein		1630.1	1673.2	1564.9	1367.1	1237.2	1176.5
Ca_11787	0.0144	-0.0096	0.0173	ATP binding/protein serine/threonine kinase [Glycine max]	GO:0004672 GO:0004674 GO:0005515 GO:0005524	520.1	527.8	470.0	382.7	435.7	439.4
Ca_18331	0.0123	-0.0069	0.0141	ATP binding/protein serine/threonine kinase [Glycine max]	GO:0004672 GO:0004674 GO:0005515 GO:0005524	1668.1	2072.6	1999.8	1400.6	1382.1	1406.2
Ca_26376	0.0101	-0.0091	0.0135	ATP binding/protein serine/threonine kinase [Glycine max]		865.1	1052.5	816.2	716.1	649.8	741.8
Ca_14236	0.0141	-0.0086	0.0165	ATPase family AAA domain-containing protein 1-like [Glycine max]	GO:0000166 GO:0005524 GO:0017111	73.3	37.9	64.3	4.9	5.1	49.8
Ca_05508	0.0139	-0.0059	0.0151	ATP-binding ABC transporter	GO:0000166 GO:0005524 GO:0016887 GO:0017111	744.4	884.4	701.6	678.2	633.4	597.1
Ca_10057	0.0124	-0.0055	0.0136	ATP-binding ABC transporter	GO:0000166 GO:0005524 GO:0016020 GO:0016887 GO:0017111	1104.3	773.3	991.0	988.2	418.6	287.8
Ca_02222	0.0141	-0.0043	0.0147	AUTOPHAGY 8E		809.5	596.2	735.5	530.5	590.9	472.1
Ca_24667	0.0092	-0.0127	0.0157	AUTOPHAGY 8E		1493.7	1330.0	1651.7	1380.1	1269.1	1208.5
Ca_22616	0.0141	-0.0086	0.0165	autophagy-related protein 8f-like isoform X4 [Glycine max]	GO:0005198	593.3	691.2	516.1	395.4	434.2	459.9
Ca_00218	0.0006	-0.0141	0.0141	Avr9/Cf-9 rapidly elicited protein		62.3	81.0	189.4	92.4	99.5	87.4
Ca_16941	0.0076	-0.0126	0.0147	B3 DNA-binding domain protein	GO:0003677	8.0	13.7	10.9	6.8	6.2	9.2
Ca_11420	0.0105	-0.0098	0.0144	B3 domain-containing transcription repressor VAL1-like isoform X4 [Glycine max]	GO:0008270	337.7	232.8	569.1	370.9	136.3	54.5
Ca_28947	0.0086	-0.0108	0.0138	Baseplate assembly protein W n=18 Tax=Enterobacteriaceae RepID=B5FS35_SALDC	GO:0005507 GO:0009308 GO:0048038	0.0	0.0	0.3	0.0	0.0	0.0
Ca_18899	0.0118	-0.0108	0.0160	beta-amyrin synthase isoform X1 [Glycine max]	GO:0003824 GO:0016866	105.0	183.6	148.4	138.7	75.8	46.1
Ca_24298	0.0145	-0.0071	0.0161	beta-xylosidase 2	GO:0004553 GO:0005975	714.1	855.1	718.8	569.2	623.9	567.7
Ca_18252	0.0146	-0.0054	0.0156	Bifunctional inhibitor/lipid-transfer protein/seed storage 2S albumin superfamily protein	GO:0006869 GO:0008289	1019.6	837.7	830.9	640.6	586.0	511.1
Ca_20247	0.0125	-0.0092	0.0155	Bifunctional orotate phosphoribosyltransferase/orotidine 5'-phosphate decarboxylase n=1 Tax=Blattabacterium sp. (Mastotermes darwiniensis) str. MADAR RepID=G7SPT8_9FLAO	GO:0003824 GO:0004588 GO:0004590	254.4	234.4	305.8	229.8	183.3	129.8
Ca_16206	0.0135	-0.0032	0.0139	blue copper protein-like [Glycine max]	GO:0005507 GO:0009055	1161.4	1720.5	1118.1	474.4	293.5	406.4
Ca_21778	0.0063	-0.0126	0.0140	BRCA1-associated protein-like [Glycine max]	GO:0005515 GO:0008270	72.2	142.2	106.3	66.4	51.4	81.2
Ca_12191	0.0096	-0.0090	0.0132	BTB/POZ domain-containing protein		142.8	149.9	116.0	88.1	98.4	86.9
Ca_03350	0.0077	-0.0125	0.0147	BTB-POZ and MATH domain 4	GO:0005515	135.4	163.0	145.6	131.2	106.5	115.8
Ca_09165	0.0138	-0.0031	0.0141	bZIP family transcription factor	GO:0003700 GO:0006355 GO:0043565	254.1	224.6	230.0	156.3	190.2	152.3
Ca_20515	0.0120	-0.0105	0.0159	c(\39S ribosomal protein L46\""		382.6	404.8	438.1	313.2	328.3	305.2
Ca_28593	0.0078	-0.0120	0.0143	c(\ABC-type proline/glycine betaine transport systems\""	GO:0005215 GO:0006810 GO:0016020	4.1	5.8	6.3	3.7	0.0	4.8
Ca_05159	0.0071	-0.0111	0.0132	c(\Actin-related protein Arp2/3 complex\""	GO:0005524 GO:0005856 GO:0005885 GO:0030833 GO:0034314	56.7	87.7	75.8	4.1	83.1	58.2
Ca_05481	0.0124	-0.0040	0.0130	c(\ADP\""	GO:0005471 GO:0005524 GO:0006810 GO:0016021	2139.7	1730.4	1772.7	1649.9	1581.3	1344.5
Ca_09643	0.0147	-0.0065	0.0161	c(\ATPase\""	GO:0015078 GO:0015991 GO:0033177 GO:0033179	1452.0	1149.9	1383.1	1123.7	1036.5	1040.3
Ca_22613	0.0108	-0.0083	0.0136	c(\cell cycle control protein\""	GO:0005634	781.8	646.4	837.5	673.6	685.2	487.4
Ca_09869	0.0129	-0.0023	0.0131	c(\complex 1 protein\""		111.8	66.2	58.9	35.7	28.1	37.5
Ca_12765	0.0138	-0.0074	0.0157	c(\crossover junction endodeoxyribonuclease\""		119.6	89.6	109.9	80.8	52.6	53.0
Ca_16881	0.0201	-0.0114	0.0231	c(\CRS2-associated factor 1\""	GO:0003723	109.4	83.0	79.5	4.8	7.0	9.6
Ca_13668	0.0123	-0.0046	0.0132	c(\cysteine desulfurase 2\""	GO:0003824 GO:0006534 GO:0008152 GO:0030170 GO:0031071	142.2	100.8	150.5	141.4	4.3	18.7
Ca_08231	0.0137	-0.0006	0.0137	c(\disease resistance protein (TIR-NBS-LRR class)\""	GO:0000166 GO:0005515 GO:0006952 GO:0007165 GO:0017111	237.0	170.2	163.2	88.9	96.5	85.7

Ca_16640	0.0109	-0.0109	0.0154	c(\DNA binding protein\""	GO:0003677 GO:0003700 GO:0005634 GO:0006355 GO:0043565	1.0	0.9	2.9	0.0	0.0	0.0
Ca_14580	0.0135	-0.0098	0.0167	c(\enoyl-CoA hydratase 2\""	GO:0008152 GO:0016491	506.9	313.2	355.4	15.5	251.2	178.4
Ca_02739	0.0115	-0.0064	0.0131	c(\external alternative NAD(P)H-ubiquinone oxidoreductase B2\""	GO:0005509 GO:0016491 GO:0050660 GO:0055114	359.3	438.9	407.3	415.1	172.5	67.6
Ca_03740	0.0129	-0.0024	0.0131	c(\F-box/kelch-repeat protein\""	GO:0005515	250.1	236.8	231.7	207.5	147.9	198.7
Ca_03813	0.0119	-0.0163	0.0202	c(\glucan endo-1\""		370.3	203.1	386.0	8.4	10.1	264.0
Ca_06874	0.0133	-0.0079	0.0154	c(\glucan endo-1\""	GO:0004553 GO:0005975	417.5	361.8	445.5	210.2	334.8	293.1
Ca_08804	0.0143	-0.0111	0.0181	c(\glucan endo-1\""	GO:0004553 GO:0005975	465.0	242.7	547.1	18.8	368.5	15.5
Ca_07943	0.0120	-0.0077	0.0142	c(\glutamate carboxypeptidase\""	GO:0006508 GO:0008233	890.6	707.1	779.5	651.1	371.5	447.7
Ca_08241	0.0130	-0.0019	0.0132	c(\glutamate carboxypeptidase\""	GO:0005634 GO:0006508 GO:0008233	912.6	686.2	722.0	554.6	656.9	412.8
Ca_16188	0.0027	-0.0139	0.0141	c(\inactive poly [ADP-ribose] polymerase SRO2\""	GO:0003950	35.2	54.7	52.9	41.5	40.8	34.6
Ca_18310	0.0102	-0.0082	0.0131	c(\mediator of RNA polymerase II transcription subunit 15a\""		2.5	8.3	0.0	0.0	0.0	0.0
Ca_19101	0.0133	-0.0017	0.0134	c(\mRNA splicing factor ATP-dependent RNA helicase\""	GO:0003676 GO:0004386 GO:0005524 GO:0008026	472.4	486.2	355.1	330.5	240.9	273.4
Ca_11864	0.0133	-0.0012	0.0133	c(\NADH-ubiquinone oxidoreductase B18 subunit\""	GO:0003954 GO:0005739 GO:0008137	2280.5	1147.4	1539.2	1199.4	983.1	891.0
Ca_00128	0.0113	-0.0069	0.0133	c(\Nse4\""	GO:0005634 GO:0006281 GO:0030915	842.9	638.5	590.4	528.6	503.9	443.1
Ca_14802	0.0043	-0.0125	0.0132	c(\nuclear factor Y\""	GO:0003700 GO:0005622 GO:0006355 GO:0016602 GO:0043565 GO:0046982	2457.8	2189.4	3355.9	2550.8	2266.1	2071.3
Ca_07861	0.0126	-0.0067	0.0143	c(\Nucleic acid-binding\""		49.5	50.5	31.2	23.6	10.3	12.1
Ca_20125	0.0069	-0.0142	0.0157	c(\Nucleic acid-binding\""		2124.4	2837.1	3406.2	2555.3	2121.4	2232.5
Ca_21356	0.0092	-0.0094	0.0131	c(\probable beta-1\""	GO:0006486 GO:0008378 GO:0016020 GO:0030246	169.4	1326.4	177.3	151.6	106.9	165.6
Ca_02337	0.0159	-0.0072	0.0175	c(\Protein of unknown function\""		2370.7	3249.2	1854.1	1378.8	1344.5	1161.2
Ca_04285	0.0138	-0.0004	0.0138	c(\Protein of unknown function\""		450.7	383.3	242.7	232.9	173.3	204.2
Ca_06619	0.0078	-0.0113	0.0137	c(\Protein of unknown function\""		284.6	436.5	366.8	278.1	325.8	309.1
Ca_01541	0.0149	-0.0013	0.0149	c(\Related to density-regulated protein\""	GO:0003743 GO:0006413	658.3	556.9	355.2	282.2	244.0	297.3
Ca_25986	0.0123	-0.0060	0.0136	c(\Retrotransposon protein\""	GO:0003676 GO:0015074	85.7	129.5	40.7	44.5	27.8	49.8
Ca_00336	0.0126	-0.0107	0.0165	c(\riboflavin biosynthesis protein\""	GO:0000166 GO:0003735	405.5	47.6	313.8	58.3	47.7	54.1
Ca_08578	0.0148	-0.0094	0.0175	c(\Signal transduction histidine kinase\""		1374.2	1257.0	1242.4	1052.9	926.7	762.7
Ca_04416	0.0019	-0.0142	0.0144	c(\small multi-drug export protein\""		166.7	345.5	249.7	208.2	211.9	217.5
Ca_15913	0.0111	-0.0073	0.0133	c(\stress enhanced protein 2\""		243.1	195.2	315.8	132.4	225.5	124.7
Ca_07805	0.0007	-0.0144	0.0144	c(\Structural constituent of ribosome\""	GO:0003735 GO:0005840 GO:0006412 GO:0019843	86.9	217.4	268.4	149.0	147.2	168.2
Ca_08676	0.0128	-0.0152	0.0199	c(\Structural constituent of ribosome\""	GO:0003735 GO:0005840 GO:0006412 GO:0015934	736.2	3065.1	1521.8	465.0	950.8	694.5
Ca_08971	0.0009	-0.0134	0.0135	c(\Sulfate/thiosulfate import ATP-binding protein cysA\""	GO:0000166 GO:0005524 GO:0016887 GO:0017111	76.3	153.6	133.1	80.8	103.6	135.4
Ca_10471	0.0119	-0.0072	0.0139	c(\thiamine thiazole synthase 2\""	GO:0006950 GO:0009228	2.1	4.5	0.0	0.0	0.0	0.0
Ca_08338	0.0112	-0.0108	0.0155	c(\threonyl-tRNA synthetase/threonine-tRNA ligase\""		3.4	3.6	0.0	0.0	0.0	0.0
Ca_14541	0.0133	0.0013	0.0134	c(\Transcription initiation factor IIF\""	GO:0003824 GO:0005524 GO:0005674 GO:0006367	417.8	366.0	238.5	200.5	164.0	193.6
Ca_21987	0.0132	-0.0007	0.0132	c(\transcription termination factor\""		455.5	335.4	330.3	255.3	261.2	242.3
Ca_28873	0.0100	-0.0122	0.0158	c(\Transcriptional regulator\""	GO:0003677 GO:0006355	10.6	6.8	3.8	0.0	0.0	5.3
Ca_03692	0.0128	-0.0132	0.0184	c(\transmembrane protein\""		0.0	1.3	0.9	0.0	0.0	0.0
Ca_05734	0.0034	-0.0126	0.0130	c(\transmembrane protein\""		249.3	457.2	284.9	289.6	202.6	253.5
Ca_09604	0.0127	-0.0079	0.0150	c(\transmembrane protein\""		233.8	302.3	272.0	246.2	128.3	158.1
Ca_17951	0.0074	-0.0133	0.0152	c(\transmembrane protein\""		275.5	318.4	344.3	292.4	238.0	273.1
Ca_22435	0.0105	-0.0101	0.0146	c(\transmembrane protein\""		1321.3	1963.1	1648.2	1176.0	1274.4	1192.7
Ca_10547	0.0128	-0.0029	0.0132	c(\tubulin alpha-6 chain\""		390.7	203.5	232.7	161.4	155.2	182.4
Ca_28542	0.0093	-0.0119	0.0151	c(\type VI secretion system effector\""		1.9	1.7	5.0	0.0	3.5	0.0
Ca_01700	0.0075	-0.0113	0.0135	c(\ubiquitin 1\""		18.8	11.4	9.2	7.8	2.5	5.6
Ca_26381	0.0143	-0.0021	0.0145	c(\Ulp1 protease family\""		157.3	85.0	61.0	11.7	10.9	31.3
Ca_29328	0.0114	-0.0074	0.0135	CAAX amino terminal protease family protein	GO:0016020	117.2	146.3	147.5	89.2	125.0	92.0
Ca_16869	0.0112	-0.0081	0.0138	caffeoyl-CoA 3-O-methyltransferase	GO:0008171	7731.8	6126.2	7339.9	4132.3	5605.1	6010.5
Ca_28415	0.0082	-0.0111	0.0138	Calcium/proton antiporter n=2 Tax=Alcaligenes RepID=M5IZ11_9BURK	GO:0016021 GO:0055085	2.0	25.3	8.1	4.6	4.8	5.7
Ca_21536	0.0140	-0.0059	0.0152	Calcium-binding EF-hand family protein	GO:0005509	195.8	137.4	180.9	124.7	81.3	84.9
Ca_15972	0.0093	-0.0146	0.0173	calmodulin-binding transcription activator 3-like isoform X1 [Glycine max]		18.2	51.4	44.2	6.5	4.6	40.9
Ca_05396	0.0142	-0.0013	0.0143	CASP-like protein 4 [Glycine max]		1100.5	722.1	517.1	286.4	396.6	490.1
Ca_05377	0.0077	-0.0111	0.0135	Cell wall protein EXP2 n=1 Tax=Mirabilis jalapa RepID=Q84L40_MIRJA	GO:0005576 GO:0009664	1858.0	1391.4	1999.4	1560.8	1186.6	1115.4
Ca_03475	0.0070	-0.0125	0.0143	Cell wall protein Exp4 n=1 Tax=Mirabilis jalapa RepID=Q84L38_MIRJA	GO:0005576 GO:0009664	235.0	325.3	319.2	271.3	252.7	226.2
Ca_08583	0.0153	-0.0030	0.0156	cell wall protein IFF6-like isoform X2 [Glycine max]		845.9	680.4	618.6	511.0	428.7	405.8
Ca_12839	0.0069	-0.0111	0.0130	Cellular nucleic acid-binding protein n=1 Tax=Medicago truncatula RepID=G7JSI2_MEDTR	GO:0003676 GO:0008270	4.2	8.0	6.8	0.0	1.6	7.8
Ca_11186	0.0132	-0.0065	0.0147	chalcone-flavanone isomerase family protein	GO:0009813 GO:0016872 GO:0045430	2522.5	2378.3	3937.8	3026.8	618.9	325.7
Ca_04027	0.0145	-0.0024	0.0147	Chaperone DnaJ-domain superfamily protein		294.5	310.2	239.0	236.2	160.3	169.0
Ca_07179	0.0046	-0.0140	0.0147	Chitinase family protein	GO:0004568 GO:0005975 GO:0006032 GO:0016998	121.1	164.3	182.2	90.4	126.8	112.6
Ca_21453	0.0153	-0.0081	0.0173	chromodomain-helicase-DNA-binding protein 1-like isoform X2 [Glycine max]	GO:0003676 GO:0003677	559.1	773.8	654.2	353.9	447.7	382.1
Ca_01425	0.0015	-0.0144	0.0145	chromosome transmission fidelity protein	GO:0000166 GO:0005524 GO:0017111	211.5	278.4	335.0	252.7	268.8	236.3
Ca_23782	0.0096	-0.0131	0.0163	chromosome-associated kinesin KIF4-like isoform X2 [Glycine max]	GO:0003777 GO:0005524 GO:0005871 GO:0007018 GO:0008017	277.0	20.5	174.4	5.2	23.7	15.3
Ca_03567	0.0013	-0.0138	0.0139	cinnamoyl coa reductase 1	GO:0003824 GO:0044237 GO:0050662	313.4	586.4	411.2	330.2	395.0	390.0
Ca_10582	0.0129	-0.0036	0.0134	cinnamyl alcohol dehydrogenase 5	GO:0008270 GO:0016491 GO:0016747 GO:0055114	1385.0	1227.2	1414.2	920.2	1167.7	970.5
Ca_08740	0.0143	0.0004	0.0143	cleavage and polyadenylation specificity factor 5	GO:0003729 GO:0005849 GO:0006378 GO:0016787	1595.4	1117.0	970.3	798.8	888.7	681.1

Ca_09462	0.0121	-0.0103	0.0159	coiled-coil domain-containing protein 94 homolog [Glycine max]		767.7	735.9	939.7	761.5	569.6	633.6
Ca_11644	0.0110	-0.0129	0.0169	condensin complex subunit 1	GO:0005488 GO:0005634 GO:0007067 GO:0007076 GO:0030261	2636.4	3106.8	3164.1	2284.9	2370.6	1911.0
Ca_28856	0.0098	-0.0091	0.0134	Conserved domain protein n=4 Tax=Klebsiella pneumoniae RepID=B5Y0D4_KLEP3		4.8	21.2	8.3	0.0	4.8	6.5
Ca_15046	0.0136	0.0009	0.0136	COP9 signalosome complex subunit 5	GO:0005515	1042.8	898.1	922.0	760.8	697.5	661.4
Ca_23392	0.0133	-0.0080	0.0156	Copper amine oxidase family protein	GO:0005507 GO:0008131 GO:0009308 GO:0048038 GO:0055114	1364.3	2830.6	1546.0	855.3	1204.5	1099.2
Ca_07656	0.0132	-0.0028	0.0135	copper/zinc superoxide dismutase 1	GO:0004784 GO:0006801 GO:0046872 GO:0055114	7863.4	6531.0	7285.8	5638.5	5818.3	5419.0
Ca_02304	0.0087	-0.0134	0.0159	Cox19-like CHCH family protein		12.2	17.2	17.2	9.5	4.0	11.9
Ca_18140	0.0132	0.0009	0.0132	cyclin-dependent kinases regulatory subunit [Glycine max]	GO:0007049 GO:0016538	1441.5	1058.9	1001.0	770.5	738.6	555.2
Ca_02157	0.0133	-0.0039	0.0138	cysteine proteinase inhibitor 1-like [Glycine max]	GO:0004869	913.0	689.7	414.5	508.7	269.1	344.4
Ca_01144	0.0111	-0.0101	0.0150	cysteine proteinase1	GO:0006508 GO:0008234	6939.8	6225.2	7161.6	5075.3	5718.7	5576.0
Ca_15460	0.0151	-0.0085	0.0173	cysteine-rich receptor-like protein kinase 10-like [Glycine max]		557.8	313.3	463.4	286.6	176.6	95.5
Ca_25020	0.0154	-0.0025	0.0156	cytochrome b5-like heme/steroid-binding domain protein	GO:0020037	1141.8	945.6	856.8	523.0	586.3	627.9
Ca_07415	0.0109	-0.0078	0.0134	cytochrome oxidase complex assembly protein		393.8	406.8	380.0	360.2	266.4	338.2
Ca_07045	0.0082	-0.0121	0.0146	Cytochrome P450 superfamily protein	GO:0005506 GO:0016705 GO:0020037 GO:0055114	4.1	15.7	5.7	0.0	3.4	6.9
Ca_19034	0.0109	-0.0100	0.0148	Cytochrome P450 superfamily protein	GO:0004497 GO:0005506 GO:0016705 GO:0020037 GO:0055114	5145.7	5351.5	5374.3	4168.3	5291.1	3108.3
Ca_20283	0.0035	-0.0127	0.0132	Cytochrome P450 superfamily protein	GO:0005506 GO:0016705 GO:0020037 GO:0055114	11.0	18.1	29.1	16.6	14.0	15.8
Ca_24971	0.0134	-0.0073	0.0153	Cytochrome P450 superfamily protein	GO:0005506 GO:0016705 GO:0020037 GO:0055114	3222.5	2532.6	2906.4	1542.8	1973.9	2025.1
Ca_06595	0.0087	-0.0111	0.0141	DEAD-box ATP-dependent RNA helicase	GO:0003676 GO:0005524 GO:0008026	4.1	6.8	8.5	4.4	4.5	0.0
Ca_07691	0.0139	-0.0010	0.0139	DEAD-box ATP-dependent RNA helicase	GO:0003676 GO:0004386 GO:0005524 GO:0008026	480.9	485.5	443.8	361.6	321.6	294.6
Ca_21580	0.0116	-0.0078	0.0140	DEAD-box ATP-dependent RNA helicase	GO:0003676 GO:0004386 GO:0005524 GO:0008026	3958.5	4822.8	4618.5	3637.7	3837.1	3236.7
Ca_14727	0.0100	-0.0085	0.0131	DEAD-box ATP-dependent RNA helicase-like protein	GO:0003676 GO:0004386 GO:0005524 GO:0008026	796.9	877.3	814.3	622.1	769.0	683.5
Ca_24794	0.0110	-0.0108	0.0154	dehydration-induced protein (ERD15)		412.8	422.4	600.8	562.0	40.7	39.8
Ca_03498	0.0047	-0.0130	0.0139	dehydration-induced protein (ERD15)		7982.9	5145.2	17241.1	5239.0	9488.9	6137.7
Ca_26205	0.0111	-0.0085	0.0140	delta subunit of Mt ATP synthase	GO:0015986 GO:0016020 GO:0046933	3957.1	3873.0	4682.4	3783.3	3599.0	3114.3
Ca_21283	0.0120	-0.0053	0.0131	dentin sialophosphoprotein-like isoform X2 [Glycine max]		255.1	302.7	279.7	216.6	214.9	223.8
Ca_02733	0.0141	-0.0049	0.0150	disease resistance protein (TIR-NBS-LRR class)	GO:0006952 GO:0043531	877.8	683.4	672.2	650.4	442.1	307.1
Ca_23843	0.0114	-0.0160	0.0197	disease-resistance response protein	GO:0006952 GO:0009607	0.0	45.8	88.4	0.0	10.8	7.2
Ca_30196	0.0115	-0.0119	0.0166	disease-resistance response protein	GO:0006952 GO:0009607	0.0	21.3	23.0	0.0	3.9	0.0
Ca_15405	0.0112	-0.0151	0.0188	DNA binding protein n=2 Tax=Arabidopsis lyrata RepID=D7L8N0_ARALL	GO:0000723 GO:0000784 GO:0003677 GO:0043047	11.0	52.9	52.4	4.5	30.6	7.7
Ca_02614	0.0117	-0.0058	0.0131	DNA ligase 1-like [Glycine max]		710.3	1026.2	847.8	876.7	340.3	234.7
Ca_26113	0.0096	-0.0143	0.0172	DNA/RNA polymerases superfamily protein n=1 Tax=Theobroma cacao RepID=UPI00042B3946		0.0	6.0	6.3	0.0	1.8	0.0
Ca_00802	0.0035	-0.0128	0.0133	DNA-binding and zinc-finger protein	GO:0005515 GO:0008270	414.4	671.0	680.1	685.6	441.4	396.4
Ca_03071	0.0096	-0.0137	0.0168	DNA-binding bromodomain-containing protein	GO:0005515	11.1	28.3	17.6	8.5	3.6	12.6
Ca_24624	0.0077	-0.0105	0.0130	DNA-binding storekeeper protein-related transcriptional regulator		534.2	593.8	693.3	532.6	560.1	454.1
Ca_05837	0.0087	-0.0107	0.0138	DNA-directed RNA polymerase-like protein	GO:0003677 GO:0003899 GO:0006351	626.6	722.2	736.8	639.5	588.6	460.8
Ca_22619	0.0149	-0.0050	0.0157	DNAJ homologue 3	GO:0003824	425.1	173.6	222.5	200.0	2.2	4.0
Ca_16342	0.0079	-0.0124	0.0147	DNAJ-like 20		394.5	364.0	403.1	353.7	280.7	311.4
Ca_05148	0.0046	-0.0135	0.0143	DOF zinc finger protein 1	GO:0003677 GO:0006355	81.4	147.9	100.9	82.1	70.8	95.8
Ca_02717	0.0017	-0.0133	0.0134	dof zinc finger protein DOF1.4 [Glycine max]	GO:0003677 GO:0006355	71.3	195.4	208.1	119.3	128.9	119.1
Ca_16653	0.0128	-0.0052	0.0138	double-stranded DNA-binding family protein	GO:0003677	1203.9	904.7	1069.3	899.4	793.2	766.6
Ca_04955	-0.0015	-0.0133	0.0134	DUF1645 family protein		159.2	170.4	285.3	211.3	172.3	170.8
Ca_21568	0.0038	-0.0140	0.0145	DUF4408 domain protein		303.4	471.0	1026.4	462.3	440.8	421.6
Ca_19142	0.0134	0.0019	0.0135	E3 ubiquitin-protein ligase CHIP-like [Glycine max]	GO:0000151 GO:0004842 GO:0005515 GO:0016567	323.6	161.6	146.5	105.3	87.7	116.7
Ca_21671	0.0107	-0.0081	0.0134	early nodulin-related		853.9	613.8	861.8	370.7	820.4	155.7
Ca_10367	0.0088	-0.0097	0.0131	emp24/gp25L/p24 family/GOLD family protein	GO:0006810 GO:0016021	1908.5	1867.3	2198.6	1869.7	1509.6	1396.0
Ca_17099	0.0051	-0.0122	0.0132	endonuclease III 2	GO:0003677 GO:0003824 GO:0006281 GO:0006284	17.1	54.1	40.0	27.3	26.9	18.9
Ca_04009	0.0142	0.0000	0.0142	endoplasmic reticulum-Golgi intermediate compartment protein 3-like [Glycine max]		849.3	741.1	702.6	567.4	567.0	555.1
Ca_13547	0.0134	-0.0027	0.0136	endoplasmic reticulum-Golgi intermediate compartment protein 3-like [Glycine max]		963.0	865.8	992.3	802.0	644.0	769.5
Ca_14627	0.0134	-0.0001	0.0134	ER membrane protein complex subunit-like protein	GO:0072546	1608.2	1453.4	1283.2	1034.3	1127.8	1075.9
Ca_07831	0.0090	-0.0098	0.0132	ethylene-responsive transcription factor 12 [Glycine max]	GO:0003677 GO:0003700 GO:0006355	79.1	89.2	56.7	45.6	38.8	39.3
Ca_09907	0.0060	-0.0128	0.0141	ethylene-responsive transcription factor 1B	GO:0003677 GO:0003700 GO:0006355	1565.0	1477.2	1953.5	1278.2	1593.9	1097.0
Ca_11327	0.0101	-0.0112	0.0151	Eukaryotic aspartyl protease family protein	GO:0004190 GO:0006508	597.4	1212.7	911.9	661.2	588.4	460.1

Ca_13844	0.0142	-0.0062	0.0155	Eukaryotic aspartyl protease family protein	GO:0004190 GO:0006508	311.5	143.4	137.4	154.8	28.1	43.4
Ca_00411	0.0161	-0.0064	0.0173	eukaryotic translation initiation factor 1A-like protein	GO:0003723 GO:0003743 GO:0006413	1208.3	1010.7	894.4	674.5	663.2	519.6
Ca_04313	0.0119	-0.0114	0.0165	eukaryotic translation initiation factor 3 subunit I	GO:0003743 GO:0005515 GO:0005737 GO:0005852	2130.6	2122.8	2513.7	2043.3	1832.2	1528.7
Ca_21835	0.0144	-0.0036	0.0149	eukaryotic translation initiation factor 5A	GO:0003723 GO:0003746 GO:0006452 GO:0008612 GO:0043022 GO:0045901 GO:0045905	12514.4	9519.3	11646.7	9064.3	8103.1	7306.0
Ca_18009	0.0160	-0.0111	0.0195	expansin B3	GO:0005576 GO:0019953	890.2	830.7	683.8	10.1	487.4	22.0
Ca_10653	0.0100	-0.0105	0.0144	FAD/NAD(P)-binding oxidoreductase	GO:0016491 GO:0055114	512.0	229.3	457.5	6.0	385.2	210.3
Ca_10225	0.0026	-0.0136	0.0138	Fatty acid hydroxylase superfamily	GO:0005506 GO:0006633 GO:0016491 GO:0055114	331.0	531.3	655.6	533.5	375.9	393.5
Ca_02822	-0.0002	-0.0130	0.0130	F-box/RNI-like superfamily protein	GO:0005515 GO:0016021	219.4	281.0	387.1	317.5	248.7	232.9
Ca_20525	0.0134	-0.0013	0.0135	Fe-S metabolism associated protein SufE		539.1	431.0	384.5	325.7	287.6	236.9
Ca_01361	0.0033	-0.0126	0.0131	fiber protein Fb15		46.3	79.0	59.9	65.7	25.8	55.3
Ca_11716	0.0077	-0.0106	0.0132	FKBP-like peptidyl-prolyl cis-trans isomerase family protein	GO:0006457	0.0	2.3	4.2	0.0	1.4	0.0
Ca_06194	0.0065	-0.0113	0.0130	Galactose oxidase/kelch repeat superfamily protein	GO:0005515	86.4	83.6	71.7	37.3	115.4	35.7
Ca_25115	0.0107	-0.0083	0.0136	Galactose oxidase/kelch repeat superfamily protein	GO:0005515	445.7	311.1	309.9	209.4	308.0	130.2
Ca_20611	0.0105	-0.0084	0.0134	GATA transcription factor 5	GO:0003677 GO:0003700 GO:0005634 GO:0006355 GO:0008270 GO:0043565 GO:0045893	342.8	416.1	325.0	271.0	275.8	245.7
Ca_04217	0.0121	-0.0136	0.0182	general regulatory factor 2	GO:0019904	15373.1	13822.2	16193.9	12783.1	12965.4	11337.6
Ca_11358	0.0047	-0.0140	0.0148	germin-like protein subfamily 3 member 2-like [Glycine max]	GO:0030145 GO:0045735	201.8	208.1	207.2	165.8	136.3	110.1
Ca_14509	0.0075	-0.0115	0.0138	glutamate receptor 3.3	GO:0004930 GO:0004970 GO:0005215 GO:0005234 GO:0006810 GO:0007186 GO:0016020	605.7	633.5	580.7	520.1	569.5	472.3
Ca_28368	0.0105	-0.0106	0.0149	Glutamine amidotransferase n=2 Tax=Enterobacter RepID=R9VWW0_9ENTR		3.5	14.9	3.1	0.0	0.0	5.7
Ca_22887	0.0141	-0.0018	0.0142	glutamine synthetase 1	GO:0003824 GO:0004356 GO:0006542 GO:0006807	3945.7	3494.0	3330.5	2749.1	2955.2	2674.1
Ca_27749	0.0106	-0.0081	0.0134	glycine-rich RNA-binding protein 3	GO:0000166 GO:0003676	1.0	3.9	0.0	0.0	0.0	0.0
Ca_06735	0.0140	-0.0029	0.0143	Glycosyltransferase family 29 (sialyltransferase) family protein	GO:0006486 GO:0008373	1359.1	933.3	979.1	830.2	798.8	690.9
Ca_08156	0.0096	-0.0117	0.0151	Golgi apparatus membrane protein tvp23 n=3 Tax=Aspergillus RepID=TVP23_ASPNC	GO:0016021	435.1	498.4	475.0	405.0	398.5	353.2
Ca_21732	0.0065	-0.0132	0.0147	GPI ethanolamine phosphate transferase 3-like isoform X3 [Glycine max]	GO:0003824 GO:0008152	59.8	100.8	106.9	16.9	114.0	18.3
Ca_24434	0.0078	-0.0112	0.0137	growth-regulating factor 5	GO:0005524 GO:0005634 GO:0006355 GO:0016818	0.0	2.6	23.2	5.6	0.0	0.0
Ca_06952	0.0164	-0.0090	0.0187	GTP-binding family protein n=1 Tax=Populus trichocarpa RepID=B9H9S8_POPTR	GO:0005525	274.8	377.0	264.0	202.8	170.9	175.7
Ca_07464	0.0146	-0.0042	0.0152	GTP-binding nuclear protein Ran-3 [Glycine max]		2438.1	2602.2	2100.6	1759.4	1811.8	1716.9
Ca_00976	0.0157	-0.0081	0.0177	GTP-binding nuclear protein Ran-3-like [Glycine max]	GO:0003924 GO:0005525 GO:0005622 GO:0006184 GO:0006886 GO:0006913 GO:0007165	441.4	535.2	335.3	237.9	290.6	193.8
Ca_14717	0.0139	-0.0049	0.0147	GTP-binding nuclear Ran-like protein	GO:0003924 GO:0005525 GO:0005622 GO:0006184 GO:0006886 GO:0006913 GO:0007165 GO:0007264 GO:0015031	829.0	1024.5	763.4	676.2	678.4	535.6
Ca_14719	0.0127	-0.0131	0.0183	GTP-binding nuclear Ran-like protein	GO:0003924 GO:0005525 GO:0005622 GO:0006184 GO:0006886 GO:0006913 GO:0007165 GO:0007264 GO:0015031	523.1	457.5	440.0	329.5	334.4	328.1
Ca_17897	0.0102	-0.0114	0.0153	GTP-binding nuclear Ran-like protein	GO:0003924 GO:0005525 GO:0005622 GO:0006184 GO:0006886 GO:0006913 GO:0007165	410.0	456.3	441.0	393.6	378.7	369.2
Ca_25427	0.0139	-0.0142	0.0199	GTP-binding nuclear Ran-like protein	GO:0003924 GO:0005525 GO:0005622 GO:0006184 GO:0006886 GO:0006913 GO:0007165 GO:0007264 GO:0015031	3712.6	3822.4	4145.8	3139.8	3307.1	2556.3
Ca_22841	0.0136	-0.0004	0.0136	guanine nucleotide-binding protein alpha-2 subunit-like isoform X1 [Glycine max]	GO:0003924 GO:0004871 GO:0007165 GO:0007186 GO:0019001 GO:0031683	819.4	786.4	620.0	543.2	579.1	574.0
Ca_09883	0.0155	-0.0025	0.0157	heat shock factor binding protein		2995.7	2352.6	1836.4	1524.3	1412.8	1144.8
Ca_26965	0.0161	-0.0045	0.0167	heat shock transcription factor A2	GO:0003700 GO:0005634 GO:0006355 GO:0009408 GO:0043565	57.4	63.5	34.6	25.0	17.4	21.1
Ca_06039	0.0137	0.0003	0.0137	high mobility group B1		6097.8	4396.2	3857.1	3411.9	3030.5	3193.4
Ca_20440	0.0146	-0.0013	0.0146	high mobility group B2		6052.9	4311.5	3900.4	3657.8	3262.0	2625.2
Ca_02373	0.0146	-0.0019	0.0148	histidine phosphotransfer protein 6	GO:0000160 GO:0004871	1227.8	1377.5	691.7	500.8	602.2	520.7
Ca_07649	0.0121	-0.0086	0.0148	histone H2A 2	GO:0000786 GO:0003677 GO:0005634 GO:0006334 GO:0046982	3378.0	3887.1	4140.8	2909.1	3266.1	2168.1
Ca_08729	0.0104	-0.0084	0.0133	histone H2A 2	GO:0000786 GO:0003677 GO:0005634 GO:0006334 GO:0046982	2076.4	1906.8	2503.1	1791.6	1871.0	1389.2
Ca_09646	0.0048	-0.0125	0.0134	histone H2A protein 9	GO:0000786 GO:0003677 GO:0005634 GO:0006334 GO:0046982	2452.3	2879.2	3605.5	2798.6	2852.0	2043.6
Ca_06009	0.0138	-0.0068	0.0154	Histone superfamily protein	GO:0000786 GO:0003677 GO:0006334 GO:0046982	78.0	154.3	127.0	28.5	12.3	30.2
Ca_12671	0.0131	-0.0003	0.0131	Histone superfamily protein	GO:0000786 GO:0003677 GO:0005634 GO:0006334 GO:0046982	2895.8	3916.7	1679.9	1259.6	1162.0	877.1
Ca_26907	0.0129	0.0024	0.0131	Histone superfamily protein	GO:0000786 GO:0003677 GO:0006334 GO:0046982	104.1	42.2	24.5	9.3	11.5	8.2
Ca_19784	0.0123	-0.0084	0.0149	histone-lysine N-methyltransferase ATX3-like isoform X2 [Glycine max]	GO:0005515 GO:0008270	474.1	568.7	385.3	426.0	265.9	216.0
Ca_21956	0.0137	-0.0050	0.0146	homeobox protein knotted-1-like 3-like isoform X3 [Glycine max]	GO:0003677 GO:0003700 GO:0005634 GO:0006355 GO:0043565	562.1	363.3	281.4	426.6	13.5	4.8
Ca_04401	0.0149	-0.0052	0.0158	HVA22 homologue C		202.9	165.5	139.6	72.1	69.8	81.4

Ca_15718	0.0009	-0.0131	0.0132	HXXXD-type acyl-transferase family protein	GO:0016747	353.4	437.3	550.5	357.2	391.9	312.6
Ca_12031	0.0095	-0.0099	0.0138	hypothetical protein		251.3	253.2	242.3	191.9	202.6	140.3
Ca_15577	0.0131	-0.0050	0.0140	hypothetical protein		19271.4	6439.4	7465.4	841.2	6807.3	4448.7
Ca_15925	0.0115	-0.0087	0.0144	hypothetical protein		552.8	375.8	344.4	307.8	297.6	284.3
Ca_16948	0.0128	0.0030	0.0132	hypothetical protein		763.1	476.3	369.5	301.2	352.3	250.9
Ca_19510	0.0113	-0.0068	0.0132	hypothetical protein		1728.4	1027.3	1482.0	902.0	1185.0	671.9
Ca_25870	0.0094	-0.0095	0.0134	hypothetical protein		0.0	2.2	0.0	0.0	0.0	0.0
Ca_26061	0.0089	-0.0106	0.0138	hypothetical protein		12.6	14.0	10.4	5.6	6.5	8.8
Ca_26867	0.0106	-0.0105	0.0149	hypothetical protein		7.8	47.8	12.8	8.8	4.2	5.0
Ca_26918	0.0080	-0.0136	0.0158	hypothetical protein		21.7	26.8	15.8	11.5	11.6	12.7
Ca_27462	0.0045	-0.0125	0.0133	hypothetical protein		5.8	12.4	15.0	11.6	5.6	8.6
Ca_27578	0.0042	-0.0130	0.0136	hypothetical protein		21.9	39.4	79.3	28.9	24.7	54.6
Ca_28151	0.0077	-0.0126	0.0148	hypothetical protein		1.4	6.9	16.4	4.1	5.7	0.0
Ca_13216	0.0129	-0.0050	0.0138	Hypoxia-responsive family protein		647.3	387.7	229.6	104.7	271.1	39.0
Ca_08526	0.0121	-0.0081	0.0146	indole-3-acetic acid inducible 14	GO:0005634 GO:0006355 GO:0046983	497.6	567.6	606.7	363.1	2.8	359.3
Ca_11212	0.0137	-0.0020	0.0138	Inosine triphosphate pyrophosphatase family protein	GO:0009143 GO:0016787 GO:0047429	244.2	249.4	173.3	149.5	103.4	110.0
Ca_28663	0.0096	-0.0088	0.0131	inosine-5'-monophosphate dehydrogenase	GO:0003824 GO:0003938 GO:0006164 GO:0055114	0.0	2.6	0.0	0.0	0.0	0.0
Ca_29642	0.0140	-0.0016	0.0141	Integral membrane family protein n=2 Tax=Malpighiales RepID=A9P8E6_POPTR	GO:0016021	260.3	201.6	152.8	93.5	93.8	115.1
Ca_27144	0.0118	-0.0061	0.0133	Integral membrane Yip1 family protein	GO:0016020	878.7	755.0	695.5	625.8	681.8	592.2
Ca_23086	0.0105	-0.0077	0.0130	Iron ion binding / oxidoreductase/ oxidoreductase protein n=4 Tax=Camelineae RepID=F4J938_ARATH	GO:0005506 GO:0016491 GO:0016706 GO:0055114	632.9	601.9	864.3	747.2	324.0	388.8
Ca_14767	0.0145	-0.0050	0.0153	kunitz trypsin inhibitor 1	GO:0004866	1826.4	1327.1	992.0	431.9	866.9	703.3
Ca_09857	0.0125	-0.0082	0.0150	late embryogenesis abundant protein	GO:0006950	1207.3	655.3	1166.1	687.6	626.9	544.4
Ca_02578	0.0058	-0.0118	0.0131	leguminosin group485 secreted peptide		50.8	195.7	107.8	29.1	149.0	43.7
Ca_20898	0.0134	-0.0023	0.0136	light harvesting-like protein		1197.0	899.0	998.2	801.3	693.6	633.4
Ca_27321	0.0049	-0.0137	0.0145	lipase 1		114.1	125.9	147.6	67.4	85.5	93.9
Ca_14000	0.0084	-0.0108	0.0137	Lipid transfer protein		1588.0	1385.1	1489.0	1142.9	1258.3	1193.3
Ca_18768	0.0135	-0.0022	0.0137	Lipid transfer protein		326.9	269.4	120.9	61.5	136.5	38.7
Ca_05826	0.0103	-0.0136	0.0170	LOB domain-containing protein 41		689.4	906.6	1038.0	554.8	779.7	424.4
Ca_17274	0.0111	-0.0162	0.0196	lysine histidine transporter 2		16.9	38.9	39.7	14.8	12.8	21.7
Ca_06732	0.0124	-0.0066	0.0141	MAP7 domain-containing protein 2-like [Glycine max]		726.1	975.9	656.2	649.7	564.9	425.3
Ca_16835	0.0081	-0.0121	0.0145	maternal effect embryo arrest 14		275.9	270.3	303.4	171.1	249.7	290.5
Ca_20464	0.0145	-0.0012	0.0146	Mechanosensitive ion channel family protein	GO:0016020 GO:0055085	304.4	113.3	92.8	26.7	52.9	47.7
Ca_18931	0.0086	-0.0121	0.0148	Mechanosensitive ion channel protein	GO:0016020 GO:0055085	0.0	9.4	6.7	0.0	2.2	3.6
Ca_10773	0.0114	-0.0077	0.0137	mediator of RNA polymerase II transcription subunit 36a	GO:0003723 GO:0006364 GO:0008033 GO:0008168	3956.0	7120.5	5659.5	5318.5	3180.4	2367.0
Ca_10868	0.0149	-0.0018	0.0150	membrane magnesium transporter		877.1	770.3	658.4	579.5	501.3	496.8
Ca_20662	0.0106	-0.0118	0.0159	methyl esterase 1		0.0	132.2	47.0	6.3	18.6	0.0
Ca_00476	0.0096	-0.0101	0.0139	methyl-CpG-binding domain protein	GO:0003677 GO:0005634	34.7	30.4	19.2	15.7	18.4	17.7
Ca_15671	0.0118	-0.0088	0.0147	Mitochondrial ATP synthase subunit G protein	GO:0000276 GO:0015078 GO:0015986	335.4	505.7	346.4	314.0	281.2	246.6
Ca_19230	0.0113	-0.0085	0.0141	multiple C2 and transmembrane domain-containing protein 2-like [Glycine max]	GO:0005515	1137.2	1315.3	1399.8	1115.7	919.1	1097.1
Ca_28393	0.0086	-0.0105	0.0136	MULTISPECIES: DNA-binding protein n=1 Tax=Vibrio RepID=UPI00030B9F0B	GO:0003677 GO:0043565	0.0	0.0	6.9	0.0	0.0	0.0
Ca_13986	0.0097	-0.0098	0.0138	myosin heavy chain-related	GO:0003774 GO:0016459	480.8	499.8	549.3	417.9	471.7	457.2
Ca_00682	0.0138	-0.0046	0.0146	NAC domain containing protein 89	GO:0003677 GO:0006355	815.4	479.7	624.1	485.5	418.2	369.2
Ca_04372	0.0126	-0.0063	0.0140	NAC domain protein 66	GO:0003677 GO:0006355	552.6	638.5	546.5	530.0	388.8	374.2
Ca_02930	0.0059	-0.0124	0.0138	NBS-LRR type disease resistance protein	GO:0043531	8.7	61.5	50.3	23.4	13.6	29.8
Ca_04493	0.0135	-0.0067	0.0150	NHL domain-containing protein	GO:0005515	683.7	571.7	639.8	570.2	389.2	338.7
Ca_08140	0.0146	-0.0062	0.0159	nicotianamine synthase 3	GO:0030410 GO:0030418	493.8	339.6	400.2	291.9	245.9	296.0
Ca_07755	0.0104	-0.0104	0.0147	Nuclear pore localisation protein NPL4		1523.9	1751.8	1915.9	1474.2	1361.3	1364.9
Ca_02151	0.0083	-0.0102	0.0131	nuclear transcription factor Y subunit B-7-like [Glycine max]	GO:0005622 GO:0043565 GO:0046982	1599.3	1404.5	1599.8	1506.1	1176.5	1241.8
Ca_05272	0.0094	-0.0090	0.0130	nucleolar complex-associated protein	GO:0005488	536.2	656.5	607.0	587.9	505.1	508.5
Ca_09875	0.0116	-0.0093	0.0149	nucleolar protein 16-like [Glycine max]		602.0	574.6	828.4	649.3	392.1	432.7
Ca_22916	0.0135	-0.0048	0.0143	nucleoporin seh1-like protein	GO:0005515	627.6	757.3	455.6	333.1	477.7	335.5
Ca_16332	0.0072	-0.0114	0.0135	nucleotide-sensitive chloride conductance regulator (ICln) family protein	GO:0000387	503.1	711.9	643.4	569.5	493.5	466.4
Ca_21898	0.0133	-0.0012	0.0133	oligopeptide transporter	GO:0055085	3116.7	3017.8	2430.5	2022.5	1956.4	1968.2
Ca_24144	0.0096	-0.0127	0.0159	Orotidine 5'-phosphate decarboxylase n=2 Tax=Schizosaccharomyces RepID=S9PQZ2_SCHOY	GO:0003824 GO:0004590 GO:0006207 GO:0008152	61.2	96.8	73.7	61.2	29.1	17.3
Ca_18787	0.0130	-0.0095	0.0161	Papain family cysteine protease	GO:0006508 GO:0008234	770.3	731.1	788.7	538.4	600.4	555.0
Ca_08280	0.0115	-0.0097	0.0150	Pectate lyase family protein		1683.4	1485.1	1432.6	1110.1	1256.0	1178.1
Ca_05311	0.0173	-0.0100	0.0199	pectinesterase/pectinesterase inhibitor 18-like [Glycine max]	GO:0004857 GO:0005618 GO:0030599 GO:0042545	5299.0	5585.4	5496.1	3788.0	3603.1	3868.7
Ca_17111	0.0105	-0.0080	0.0132	Pentatricopeptide repeat (PPR) superfamily protein		642.9	459.9	703.2	553.2	426.7	410.9
Ca_03178	0.0117	-0.0119	0.0167	Pentatricopeptide repeat (PPR) superfamily protein		5.1	0.0	5.3	0.0	0.0	0.0
Ca_02524	0.0095	-0.0113	0.0148	peptide transporter 1	GO:0005215 GO:0006810 GO:0006857 GO:0016020	703.4	431.7	570.1	483.9	327.4	212.9
Ca_08773	0.0106	-0.0126	0.0165	pfkB-like carbohydrate kinase family protein		369.8	329.3	349.0	204.3	282.4	277.4
Ca_02560	0.0011	-0.0145	0.0145	Phosphate-responsive 1 family protein		112.2	259.6	345.1	191.1	164.0	217.7
Ca_15012	0.0124	-0.0043	0.0131	phosphofructokinase 3	GO:0003872 GO:0005524 GO:0005945 GO:0006002 GO:0006096	1538.2	2156.7	1281.0	1221.0	1157.3	892.1
Ca_07644	0.0158	-0.0074	0.0175	Phosphoglycerate mutase family protein	GO:0003824 GO:0008152	364.7	291.9	185.0	156.3	132.7	126.4
Ca_29841	0.0092	-0.0102	0.0137	phosphoinositide phosphatase SAC1-like isoform X1 [Glycine max]	GO:0005515 GO:0042578	0.0	11.6	8.8	0.0	3.8	2.1
Ca_30220	0.0017	-0.0139	0.0140	phosphopantothenoylcysteine decarboxylase subunit SIS2-like [Glycine max]		48.5	106.9	99.5	75.3	61.1	77.2
Ca_08904	0.0024	-0.0132	0.0134	plant/MNJ8-150 protein		93.1	90.3	113.3	75.1	99.7	73.3

Ca_09144	0.0160	-0.0054	0.0169	plant-specific B3-DNA-binding domain protein	GO:0003677	962.9	1053.7	844.0	727.3	651.9	573.9
Ca_09665	0.0137	-0.0070	0.0154	Plastid-lipid associated protein PAP / fibrillin family protein	GO:0005198 GO:0009507	105.5	125.8	118.8	69.8	91.9	60.5
Ca_11935	0.0046	-0.0139	0.0146	Plastid-lipid associated protein PAP / fibrillin family protein	GO:0005198 GO:0009507	742.7	797.3	1194.3	856.5	780.1	763.3
Ca_13288	0.0138	-0.0028	0.0141	Pollen Ole e 1 allergen and extensin family protein		9205.1	6364.9	7128.2	4691.5	5374.0	4855.8
Ca_06149	0.0136	-0.0068	0.0152	poly(A)-specific ribonuclease PARN-like [Glycine max]	GO:0003676 GO:0005634	239.2	281.4	184.8	160.0	161.8	170.1
Ca_09005	0.0032	-0.0134	0.0138	poly(rC)-binding protein 1-like [Glycine max]	GO:0003723	1182.7	1347.3	1690.4	1353.9	1274.1	988.0
Ca_11128	0.0076	-0.0136	0.0155	polygalacturonase-like [Glycine max]	GO:0004650 GO:0005975	11.6	31.7	40.8	13.7	13.4	21.9
Ca_27803	0.0060	-0.0126	0.0139	Polyprotein n=1 Tax=Citrus endogenous pararetrovirus RepID=V9QEM3_9RETR		33.3	43.3	31.5	14.8	27.4	32.2
Ca_22380	0.0131	-0.0071	0.0149	post-GPI attachment-like factor-protein		892.6	868.2	938.8	708.9	612.5	773.3
Ca_09726	0.0046	-0.0138	0.0145	PPPDE putative thiol peptidase family protein		187.8	237.1	233.3	121.9	186.1	128.7
Ca_12204	0.0115	-0.0115	0.0162	prefoldin 2	GO:0006457 GO:0016272 GO:0051082	1026.6	873.9	1063.8	888.8	818.5	694.9
Ca_17340	0.0140	-0.0026	0.0143	pre-mRNA-processing protein 40C-like isoform X2 [Glycine max]	GO:0005515	264.4	132.9	113.8	128.4	10.3	5.0
Ca_05422	0.0186	-0.0073	0.0200	pre-mRNA-splicing factor SF2-like isoform X3 [Glycine max]	GO:0000166 GO:0003676	247.0	112.1	111.3	8.5	9.6	15.8
Ca_29771	0.0020	-0.0133	0.0134	probable calcium-binding protein CML20 [Glycine max]	GO:0005509	1011.1	1679.8	1700.8	1354.3	1270.5	1099.0
Ca_22949	0.0016	-0.0130	0.0131	probable calcium-binding protein CML27-like [Glycine max]	GO:0005509	1800.7	1176.8	2454.2	1735.7	1140.8	1340.2
Ca_21444	0.0153	-0.0064	0.0166	probable galacturonosyltransferase 11-like [Glycine max]	GO:0016757	1945.3	2041.2	1758.5	1466.7	1399.7	1156.0
Ca_14764	0.0083	-0.0104	0.0133	probable glycosyltransferase At5g03795-like [Glycine max]		56.5	74.8	54.8	31.9	55.8	41.8
Ca_10148	0.0115	-0.0091	0.0147	probable membrane-associated kinase regulator 5-like [Glycine max]		195.6	214.5	240.4	193.6	120.0	178.1
Ca_16821	0.0017	-0.0144	0.0145	probable phosphoinositide phosphatase SAC9-like [Glycine max]	GO:0005515 GO:0042578	137.2	231.4	369.6	141.5	151.0	207.2
Ca_08793	0.0100	-0.0093	0.0137	probable signal peptidase complex subunit 1-like isoform X2 [Glycine max]	GO:0005787 GO:0006465 GO:0008233 GO:0016021	468.6	481.4	499.9	375.5	384.0	319.9
Ca_15261	0.0139	-0.0010	0.0140	probable sugar phosphate/phosphate translocator [Glycine max]		434.1	309.0	73.2	63.5	5.9	48.7
Ca_12282	0.0128	-0.0025	0.0131	prohibitin 1	GO:0016020	583.0	548.5	574.5	463.2	444.0	385.9
Ca_06980	0.0121	-0.0056	0.0134	proteasome beta type-3 subunit	GO:0004175 GO:0004298 GO:0005839 GO:0051603	1163.1	1618.2	1246.8	994.8	1022.1	839.3
Ca_13351	0.0151	-0.0018	0.0152	proteasome subunit beta type-7-A protein	GO:0004175 GO:0004298 GO:0005839 GO:0051603	1126.9	1036.3	1013.8	793.4	803.4	758.0
Ca_29151	0.0088	-0.0108	0.0139	Protein containing DUF1203 n=2 Tax=Pseudovibrio RepID=G8PMS2_PSEUV		2.8	13.6	6.1	4.2	0.0	6.2
Ca_23694	0.0106	-0.0110	0.0153	Protein kinase protein with adenine nucleotide alpha hydrolases-like domain	GO:0016772	0.0	0.0	0.6	0.0	0.0	0.0
Ca_00488	0.0149	-0.0006	0.0149	Protein kinase superfamily protein	GO:0004672 GO:0004674 GO:0005524 GO:0006468 GO:0016772	261.6	56.8	45.4	13.6	11.9	14.4
Ca_01942	0.0106	-0.0080	0.0133	Protein kinase superfamily protein	GO:0004672 GO:0004674 GO:0005524 GO:0006468 GO:0016772	452.3	449.6	953.0	341.4	328.7	336.2
Ca_02352	0.0119	-0.0087	0.0148	Protein kinase superfamily protein	GO:0004672 GO:0004674 GO:0005524 GO:0006468 GO:0016772	54.9	82.1	70.6	18.1	85.2	10.2
Ca_03187	0.0111	-0.0083	0.0138	Protein kinase superfamily protein	GO:0004672 GO:0005524 GO:0006468 GO:0016772	255.3	253.0	213.4	198.5	163.3	150.6
Ca_15990	0.0085	-0.0107	0.0137	Protein kinase superfamily protein	GO:0004672 GO:0005524 GO:0006468 GO:0016772	0.0	18.9	10.4	6.8	4.3	0.0
Ca_20813	0.0142	0.0000	0.0142	Protein kinase superfamily protein	GO:0004672 GO:0004674 GO:0005524 GO:0006468 GO:0016772	1339.0	1226.9	962.4	789.6	861.2	814.1
Ca_27127	0.0110	-0.0085	0.0139	Protein kinase superfamily protein	GO:0004672 GO:0004674 GO:0005524 GO:0006468 GO:0016772	297.5	423.4	459.2	363.0	237.4	230.2
Ca_03043	0.0139	-0.0083	0.0162	protein LATERAL ROOT PRIMORDIUM 1-like isoform X3 [Glycine max]		1712.9	1383.5	1624.6	1425.5	1051.9	1102.7
Ca_11121	0.0129	-0.0035	0.0134	Protein of unknown function (DUF167)		138.7	64.4	85.5	63.1	41.2	48.7
Ca_15851	0.0113	-0.0087	0.0142	Protein of unknown function (DUF506)		946.4	431.8	865.4	360.7	477.0	292.8
Ca_18995	0.0023	-0.0141	0.0143	Protein of unknown function (DUF789)		94.1	94.1	120.8	70.0	82.4	89.6
Ca_09302	0.0101	-0.0127	0.0163	Protein phosphatase 2C family protein	GO:0003824 GO:0004722 GO:0006470	0.4	3.2	7.4	2.6	0.0	0.0
Ca_03544	0.0148	-0.0080	0.0168	protein transport protein Sec61 subunit gamma [Glycine max]	GO:0006605 GO:0006886 GO:0015031 GO:0015450 GO:0016020	987.8	1215.8	1073.5	760.2	832.2	669.0
Ca_18037	0.0119	-0.0126	0.0173	protein ULTRAPETALA 1-like [Glycine max]	GO:0003677	466.3	562.0	517.5	388.0	354.9	388.9
Ca_04571	0.0145	-0.0055	0.0155	protein YIF1B-like isoform X3 [Glycine max]		893.9	722.4	765.4	585.7	603.5	536.0
Ca_04522	0.0110	-0.0092	0.0143	protein yippee-like isoform X2 [Glycine max]		0.0	10.6	4.5	0.0	0.0	0.0
Ca_04704	0.0121	-0.0079	0.0144	protein YLS7-like [Glycine max]		445.2	272.7	360.3	8.7	271.1	211.7
Ca_16949	0.0148	-0.0064	0.0162	purin-rich alpha 1		429.8	247.7	188.3	7.9	10.3	188.1
Ca_01555	0.0110	-0.0160	0.0194	purple acid phosphatase 28	GO:0016787	107.7	93.5	162.8	12.7	7.4	115.8
Ca_07336	0.0092	-0.0117	0.0149	putative disease resistance protein RGA1-like [Glycine max]		0.0	0.0	17.4	0.0	0.0	0.0
Ca_24663	0.0157	-0.0093	0.0183	putative L-ascorbate peroxidase 6-like [Glycine max]		183.2	94.7	158.4	0.0	37.2	68.8
Ca_05940	0.0076	-0.0131	0.0152	putative nuclear matrix constituent protein 1-like protein-like [Glycine max]		0.0	7.3	7.6	0.0	4.3	1.0
Ca_00023	0.0122	-0.0085	0.0148	pyruvate decarboxylase-2	GO:0003824 GO:0030976	286.5	336.3	355.4	167.4	266.4	168.0
Ca_18196	0.0017	-0.0131	0.0132	pyruvate decarboxylase-2	GO:0000287 GO:0003824 GO:0030976	228.6	357.9	390.5	252.7	350.1	288.1
Ca_05684	0.0131	-0.0122	0.0179	pyruvate dehydrogenase kinase	GO:0005524 GO:0016310 GO:0016772	350.4	382.7	398.3	284.5	311.3	329.6
Ca_04989	0.0102	-0.0092	0.0138	Pyruvate kinase family protein	GO:0000287 GO:0003824 GO:0004743 GO:0006096 GO:0030955	2906.1	4051.4	3836.1	2444.0	3119.9	2500.6
Ca_21269	0.0081	-0.0131	0.0154	quinolinate synthase	GO:0008987 GO:0009435	1081.8	801.5	1139.7	867.1	659.0	752.2
Ca_16204	0.0152	-0.0041	0.0157	RAB GDP dissociation inhibitor 2	GO:0005093 GO:0015031	7629.9	6329.2	6818.4	5755.0	5306.9	4710.3

Ca_10190	0.0129	-0.0020	0.0131	RAN binding protein 1	GO:0046907	2437.8	2661.7	2399.2	1944.5	2170.2	1797.1
Ca_06213	0.0132	-0.0133	0.0188	receptor-like kinase	GO:0016772	0.0	5.5	3.9	0.0	0.0	0.0
Ca_08737	0.0150	-0.0034	0.0154	Remorin family protein		3287.6	2308.8	2399.5	1805.3	1752.8	1577.1
Ca_13600	0.0143	-0.0016	0.0144	Rho GDP-dissociation inhibitor-like protein	GO:0005094 GO:0005737	349.5	316.3	204.0	170.3	173.0	180.7
Ca_09061	0.0121	-0.0069	0.0139	Rho GTPase		418.4	370.0	317.0	305.1	222.8	182.6
Ca_13314	0.0054	-0.0119	0.0130	Rhodanese/Cell cycle control phosphatase superfamily protein		416.3	439.0	407.1	413.8	308.5	296.6
Ca_16344	0.0139	-0.0031	0.0143	rhodanese/cell cycle control phosphatase superfamily protein		285.6	213.5	233.5	209.4	156.0	138.4
Ca_23345	0.0125	-0.0043	0.0132	rhodanese-related sulfurtransferase		88.1	115.7	50.4	27.7	51.4	29.7
Ca_20883	0.0123	-0.0099	0.0158	ribose-phosphate pyrophosphokinase	GO:0000287 GO:0004749 GO:0009156 GO:0009165 GO:0044249	342.6	374.2	373.3	281.8	300.6	253.3
Ca_16459	0.0033	-0.0127	0.0131	Ribosomal RNA large subunit methyltransferase F n=1 Tax=Pseudomonas alcaligenes OT 69 RepID=U3H9W9_PSEAC	GO:0008168	81.7	75.9	108.1	91.4	48.7	74.6
Ca_00527	0.0096	-0.0114	0.0149	RING/U-box superfamily protein	GO:0008270	6.9	8.2	14.7	3.1	1.3	4.8
Ca_12384	0.0137	-0.0012	0.0137	RING/U-box superfamily protein	GO:0005515 GO:0008270	905.0	447.5	357.0	226.9	283.0	185.0
Ca_15815	0.0072	-0.0122	0.0141	RING-H2 finger protein 2B	GO:0005515 GO:0008270	686.7	461.6	659.3	453.6	443.7	412.7
Ca_07460	0.0132	-0.0020	0.0133	RNA-binding (RRM/RBD/RNP motifs) family protein	GO:0000166 GO:0003676	862.5	879.0	743.6	687.9	532.6	515.3
Ca_20567	0.0117	-0.0081	0.0142	RNA-binding (RRM/RBD/RNP motifs) family protein	GO:0000166 GO:0003676	1214.8	1015.3	1330.4	983.3	1086.5	827.1
Ca_24135	0.0089	-0.0105	0.0138	RNA-binding KH domain-containing protein	GO:0003723	0.0	0.0	2.7	0.0	0.0	0.0
Ca_18811	0.0134	-0.0116	0.0177	RNA-binding protein 1	GO:0000166 GO:0003676	3408.2	3422.3	3716.4	2526.4	2708.9	1891.7
Ca_22359	0.0018	-0.0143	0.0144	RNA-binding protein 1	GO:0000166 GO:0003676	115.8	287.2	316.8	248.0	135.1	173.9
Ca_23966	0.0109	-0.0097	0.0146	RNA-binding protein 28-like isoform X2 [Glycine max]		2.1	17.1	6.3	0.0	0.0	0.0
Ca_04430	0.0118	-0.0093	0.0150	RNA-directed DNA polymerase homolog [Glycine max]	GO:0003723 GO:0003964 GO:0006278	86.6	138.9	97.9	73.6	69.1	76.6
Ca_13619	0.0134	-0.0052	0.0144	Rubber elongation factor protein (REF)		589.1	442.2	522.0	333.9	382.0	386.2
Ca_17495	0.0120	-0.0082	0.0145	SAUR-like auxin-responsive protein family		0.7	0.8	0.0	0.0	0.0	0.0
Ca_15997	0.0136	-0.0057	0.0148	Sec14p-like phosphatidylinositol transfer family protein	GO:0006810 GO:0016021	2704.8	2177.7	1984.9	1597.3	1401.8	1734.6
Ca_18035	0.0131	-0.0024	0.0133	Secretory carrier membrane protein (SCAMP) family protein	GO:0015031 GO:0016021	1380.4	986.5	1102.2	901.2	926.1	818.5
Ca_22442	0.0118	-0.0122	0.0170	Secretory carrier membrane protein (SCAMP) family protein	GO:0015031 GO:0016021	848.3	1004.5	930.4	651.9	762.2	778.3
Ca_11442	0.0063	-0.0118	0.0133	seed maturation protein	GO:0009790	12.9	13.2	37.8	8.3	19.3	7.4
Ca_22654	0.0041	-0.0136	0.0142	serine/threonine-protein kinase SAPK3-like [Glycine max]	GO:0004672 GO:0004674 GO:0005524 GO:0006468 GO:0016772	93.9	207.7	172.0	125.1	96.6	117.1
Ca_00652	0.0086	-0.0100	0.0132	serine/threonine-protein phosphatase 7 long form homolog [Glycine max]		9.0	13.8	15.9	10.6	4.9	9.9
Ca_04114	0.0109	-0.0097	0.0146	serine/threonine-protein phosphatase 7 long form homolog [Glycine max]		2.0	11.8	0.0	0.0	0.0	0.0
Ca_25348	0.0083	-0.0118	0.0144	serine/threonine-protein phosphatase 7 long form homolog [Glycine max]		0.0	0.0	5.7	0.0	0.0	0.0
Ca_27068	0.0102	-0.0139	0.0172	serine/threonine-protein phosphatase 7 long form homolog [Glycine max]		28.8	17.8	27.7	11.4	7.5	14.0
Ca_15786	0.0131	-0.0022	0.0133	short-chain dehydrogenase/reductase	GO:0008152 GO:0016491	94.8	103.6	63.8	46.7	28.0	27.8
Ca_16057	0.0103	-0.0111	0.0151	sieve element occlusion protein		3.2	2.7	1.7	0.0	1.4	0.0
Ca_24230	0.0139	-0.0061	0.0151	SLL1 protein		1789.7	1711.6	1636.6	1164.3	1396.2	1102.0
Ca_15242	0.0128	-0.0031	0.0132	Small nuclear ribonucleoprotein family protein	GO:0008380 GO:0030532	1886.1	2198.8	1508.9	1464.9	1093.5	1039.5
Ca_10111	0.0076	-0.0110	0.0134	SNARE associated Golgi protein family		628.2	771.2	746.3	725.1	574.0	478.1
Ca_04488	0.0129	-0.0031	0.0133	spermidine synthase 1	GO:0003824	914.1	1083.3	794.3	635.8	713.2	611.9
Ca_02676	0.0112	-0.0075	0.0135	sphingolipid delta desaturase	GO:0006629	457.8	1109.6	348.5	353.8	305.8	210.1
Ca_04475	0.0112	-0.0069	0.0131	Sterile alpha motif (SAM) domain-containing protein	GO:0005515	526.5	475.5	584.3	449.8	425.3	344.2
Ca_21881	0.0130	0.0012	0.0130	Stress responsive alpha-beta barrel domain protein		466.2	211.2	243.4	178.0	155.4	132.1
Ca_03191	0.0121	-0.0055	0.0133	Structural constituent of ribosome n=1 Tax=Zea mays RepID=B6TUI1_MAIZE	GO:0003735 GO:0005622 GO:0005840 GO:0006412	225.8	207.6	218.1	167.1	160.2	95.9
Ca_25647	0.0055	-0.0139	0.0149	subtilisin-like protease-like isoform X2 [Glycine max]	GO:0004252 GO:0006508	78.4	79.7	174.8	57.2	88.2	50.5
Ca_20061	0.0083	-0.0130	0.0154	subtilisin-like serine protease 2	GO:0004252 GO:0006508	27.6	77.8	44.9	16.9	25.0	36.2
Ca_23968	0.0108	-0.0085	0.0137	sugar transport protein 10-like [Glycine max]	GO:0005215 GO:0006810 GO:0016020 GO:0016021 GO:0022857 GO:0022891 GO:0055085	5.4	23.9	3.8	0.0	1.6	0.0
Ca_15866	0.0139	-0.0030	0.0142	Sugar transporter SWEET n=3 Tax=Phaseoleae RepID=C6TC24_SOYBN	GO:0016021	1822.2	1761.1	957.8	824.1	684.8	912.3
Ca_14668	0.0016	-0.0132	0.0133	SWIM zinc finger family protein / mitogen-activated protein kinase kinase kinase (MAPKKK)-related	GO:0005515 GO:0008270	183.6	148.6	311.4	194.3	179.8	135.7
Ca_12520	0.0137	-0.0068	0.0153	syntaxin of plants 51	GO:0005515	713.8	663.0	671.4	571.5	613.2	575.9
Ca_05669	0.0136	0.0013	0.0136	T-complex protein 1 subunit beta-like [Glycine max]	GO:0005524 GO:0006457	1078.7	934.1	776.8	576.2	646.3	530.5
Ca_20841	0.0075	-0.0114	0.0137	telomere repeat-binding factor 2-like isoform X2 [Glycine max]	GO:0000786 GO:0003677 GO:0003682 GO:0005634 GO:0006334	26.6	38.7	24.3	18.0	16.8	23.7
Ca_22847	0.0034	-0.0133	0.0137	Tetratricopeptide repeat (TPR)-like superfamily protein	GO:0005515	302.6	382.6	462.4	404.9	300.3	320.8
Ca_02286	0.0130	-0.0062	0.0144	thioredoxin-dependent peroxidase 1	GO:0016491	538.4	528.3	618.7	490.5	438.6	384.0
Ca_25779	0.0109	-0.0117	0.0160	Threonyl-tRNA synthetase	GO:0000166 GO:0004812 GO:0004829 GO:0005524 GO:0005737 GO:0006418 GO:0006435	8.8	21.6	16.1	4.4	8.7	11.6
Ca_13335	0.0135	0.0014	0.0136	THUMP domain-containing protein	GO:0003723	1116.5	812.3	729.4	643.9	547.8	516.6
Ca_23009	0.0136	-0.0057	0.0147	trafficking protein particle complex subunit-like protein	GO:0005801 GO:0006810 GO:0006888	719.1	573.5	674.7	487.0	537.0	420.0
Ca_14670	0.0130	-0.0017	0.0131	transcription factor bHLH93-like [Glycine max]	GO:0046983	1914.3	816.4	649.2	452.3	619.2	461.4

Ca_05685	0.0126	-0.0051	0.0136	transcription factor GTE8-like isoform X2 [Glycine max]	GO:0005515	1757.6	1136.1	1369.6	1089.0	1064.2	940.6
Ca_00998	0.0107	-0.0112	0.0155	transcription factor HBP-1b(c1)-like isoform X2 [Glycine max]	GO:0006351 GO:0043565	47.0	76.5	34.4	35.7	17.4	22.8
Ca_06948	0.0136	-0.0036	0.0141	transcription factor IIB	GO:0006352 GO:0006355 GO:0008270 GO:0017025	597.2	587.4	573.0	539.8	417.3	303.8
Ca_05114	0.0074	-0.0121	0.0142	transcription factor IIIB 90 kDa subunit-like isoform X2 [Glycine max]	GO:0005634 GO:0006352 GO:0006355 GO:0008270 GO:0045893	13.1	27.9	52.7	16.9	21.2	17.5
Ca_15186	0.0109	-0.0073	0.0131	transcription factor RADIALIS-like [Glycine max]	GO:0003677 GO:0003682	2.9	22.2	11.5	0.0	5.0	0.0
Ca_14571	0.0119	-0.0081	0.0144	Transducin/WD40 repeat-like superfamily protein	GO:0005515	262.1	330.6	270.9	227.0	227.2	229.0
Ca_18117	0.0139	0.0000	0.0139	Translation initiation factor SUI1 family protein	GO:0003743 GO:0006413	813.0	560.4	541.9	399.5	349.8	387.4
Ca_12569	0.0134	-0.0084	0.0158	transmembrane amino acid transporter family protein		221.1	94.4	144.5	62.5	92.3	64.1
Ca_18042	0.0094	-0.0127	0.0158	tubulin beta chain 2	GO:0003924 GO:0005200 GO:0005525 GO:0005874	4600.3	5150.0	5529.9	4290.2	4611.5	3340.6
Ca_01987	0.0017	-0.0135	0.0136	tubulin beta chain 4	GO:0003924	3833.2	4622.7	5257.4	4060.9	4800.2	3837.2
Ca_11939	0.0135	0.0000	0.0135	type II superfamily restriction endonuclease	GO:0003677 GO:0004518	257.3	232.3	185.8	170.1	132.2	135.9
Ca_17740	0.0091	-0.0123	0.0153	UBA and UBX domain-containing protein At4g15410-like [Glycine max]	GO:0005515	0.0	0.0	10.0	0.0	0.0	0.0
Ca_15827	0.0147	-0.0019	0.0148	ubiquitin carboxyl-terminal hydrolase	GO:0006511	583.2	399.6	327.6	315.4	255.2	242.2
Ca_24848	0.0132	0.0008	0.0132	ubiquitin ligase SINAT3	GO:0004842 GO:0005515 GO:0005634 GO:0006511 GO:0007275 GO:0008270 GO:0016567	422.1	252.1	271.5	182.4	154.6	154.8
Ca_18445	0.0150	-0.0038	0.0155	ubiquitin-conjugating enzyme 13	GO:0016881	2462.2	2065.3	2034.9	1762.1	1379.7	1457.7
Ca_24777	0.0110	-0.0100	0.0148	ubiquitin-conjugating enzyme 3	GO:0016881 GO:0019789	554.7	456.3	617.1	400.2	438.7	344.3
Ca_15161	0.0142	-0.0018	0.0144	ubiquitin-conjugating enzyme 34	GO:0016881	650.4	575.9	513.6	388.2	488.7	395.6
Ca_09356	0.0093	-0.0124	0.0155	U-box domain-containing protein 4 [Glycine max]	GO:0005488 GO:0005515	22.1	55.7	55.5	21.7	26.9	24.8
Ca_21597	0.0039	-0.0141	0.0146	ultraviolet-B-repressible protein	GO:0009523 GO:0015979 GO:0016020	62.9	532.1	438.4	234.1	119.7	159.8
Ca_26642	0.0078	-0.0137	0.0158	uncharacterized mitochondrial protein AtMg00810-like [Glycine max]		37.4	48.7	38.6	29.8	28.3	30.1
Ca_18951	0.0083	-0.0118	0.0144	uncharacterized mitochondrial protein AtMg00810-like [Glycine max]		0.0	0.0	5.7	0.0	0.0	0.0
Ca_26542	0.0110	-0.0077	0.0135	uncharacterized mitochondrial protein AtMg00810-like [Glycine max]		2.1	7.1	0.0	0.0	0.0	0.0
Ca_10875	0.0025	-0.0146	0.0149	uncharacterized protein At4g15970-like isoform X1 [Glycine max]		223.1	330.1	377.8	282.4	217.2	209.8
Ca_02140	0.0035	-0.0140	0.0145	uncharacterized protein At5g39865-like [Glycine max]	GO:0009055 GO:0015035 GO:0045454	88.9	103.5	120.0	103.0	71.0	79.8
Ca_19134	0.0089	-0.0131	0.0158	uncharacterized protein LOC100777580 isoform X7 [Glycine max]		5.0	11.4	11.4	7.8	2.9	5.6
Ca_17140	0.0076	-0.0113	0.0136	uncharacterized protein LOC100777625 isoform X5 [Glycine max]		15.3	24.7	40.2	23.5	15.7	8.2
Ca_25220	0.0127	-0.0050	0.0136	uncharacterized protein LOC100778822 isoform X6 [Glycine max]		158.2	159.9	160.4	132.8	97.2	111.0
Ca_16240	0.0109	-0.0091	0.0142	uncharacterized protein LOC100778886 [Glycine max]		313.9	220.4	342.0	306.2	158.4	145.4
Ca_24421	0.0088	-0.0121	0.0150	uncharacterized protein LOC100782697 [Glycine max]		290.8	233.9	350.8	304.5	89.2	83.5
Ca_14741	0.0132	-0.0044	0.0139	uncharacterized protein LOC100789230 isoform X2 [Glycine max]		207.9	216.4	128.3	86.6	100.0	102.5
Ca_25872	0.0010	-0.0145	0.0145	uncharacterized protein LOC100791744 [Glycine max]		189.8	210.9	255.8	194.2	203.6	177.3
Ca_13030	0.0112	-0.0067	0.0130	uncharacterized protein LOC100792233 [Glycine max]		923.3	721.7	710.6	605.5	622.6	616.2
Ca_02331	0.0120	-0.0099	0.0155	uncharacterized protein LOC100792354 isoform X1 [Glycine max]		165.5	242.1	236.8	42.7	158.9	163.6
Ca_21436	0.0052	-0.0135	0.0145	uncharacterized protein LOC100792679 isoform X2 [Glycine max]		45.5	109.9	79.7	49.4	73.6	44.9
Ca_13072	0.0117	-0.0111	0.0161	uncharacterized protein LOC100793067 isoform X3 [Glycine max]		289.4	285.6	322.6	228.4	262.9	187.2
Ca_20028	0.0130	-0.0030	0.0134	uncharacterized protein LOC100798943 [Glycine max]		391.4	281.4	251.3	206.4	220.9	183.1
Ca_24596	0.0130	-0.0096	0.0162	uncharacterized protein LOC100800595 isoform X4 [Glycine max]		1407.1	1326.0	1280.4	157.9	675.9	1158.2
Ca_26236	0.0083	-0.0118	0.0144	uncharacterized protein LOC100806437 [Glycine max]		0.0	0.0	5.7	0.0	0.0	0.0
Ca_16419	0.0057	-0.0120	0.0133	uncharacterized protein LOC100807379 isoform X3 [Glycine max]		80.4	145.0	114.4	114.5	69.3	73.3
Ca_24942	0.0107	-0.0155	0.0189	uncharacterized protein LOC100809313 [Glycine max]		8.2	10.6	11.2	0.0	6.3	5.8
Ca_23453	0.0044	-0.0125	0.0133	uncharacterized protein LOC100812827 [Glycine max]		25.6	65.4	61.5	36.9	29.2	39.7
Ca_21129	0.0144	-0.0020	0.0145	uncharacterized protein LOC100819690 isoform X2 [Glycine max]		1190.6	852.7	927.6	799.8	647.0	636.3
Ca_01973	0.0114	-0.0145	0.0185	uncharacterized protein LOC100820019 isoform X5 [Glycine max]		16.4	30.5	18.4	6.8	9.4	10.3
Ca_15402	0.0029	-0.0128	0.0132	uncharacterized protein LOC100820022 [Glycine max]		30.1	43.8	47.4	24.7	41.0	37.4
Ca_01349	0.0117	-0.0063	0.0133	uncharacterized protein LOC100820117 [Glycine max]		847.8	1375.9	617.4	434.8	826.5	460.6
Ca_19894	0.0092	-0.0125	0.0155	uncharacterized protein LOC100820117 [Glycine max]		0.0	0.0	1.6	0.0	0.0	0.0
Ca_04375	0.0112	-0.0093	0.0145	uncharacterized protein LOC102659397 [Glycine max]		16.9	2.7	12.7	5.9	0.0	0.0
Ca_16166	0.0088	-0.0133	0.0159	uncharacterized protein LOC102662319 [Glycine max]		47.2	97.9	104.5	34.2	31.9	93.6

Ca_12047	0.0052	-0.0122	0.0132	uncharacterized protein LOC102663500 [Glycine max]		9.3	20.8	11.9	7.4	8.3	12.0
Ca_24738	0.0094	-0.0095	0.0134	uncharacterized protein LOC102663815 [Glycine max]		0.0	2.2	0.0	0.0	0.0	0.0
Ca_29926	0.0082	-0.0117	0.0143	uncharacterized protein LOC102664551 [Glycine max]		0.0	4.2	7.2	0.0	2.2	0.0
Ca_27353	0.0097	-0.0093	0.0135	uncharacterized protein LOC102664679 isoform X1 [Glycine max]		0.8	10.5	10.1	0.0	4.1	0.0
Ca_20146	0.0107	-0.0109	0.0153	uncharacterized protein LOC102664679 isoform X2 [Glycine max]		482.4	554.8	554.9	406.5	364.8	440.4
Ca_27182	0.0077	-0.0111	0.0135	uncharacterized protein LOC102664679 isoform X2 [Glycine max]		0.0	0.0	5.4	0.0	0.0	0.0
Ca_03246	0.0117	-0.0144	0.0186	uncharacterized protein LOC102664679 isoform X2 [Glycine max]		2.4	2.4	8.1	0.0	2.5	0.0
Ca_27565	0.0108	-0.0083	0.0136	uncharacterized protein LOC102670328 [Glycine max]		15.7	7.9	7.5	0.0	3.8	0.0
Ca_19751	0.0129	-0.0019	0.0130	uncharacterized protein LOC547764 isoform X3 [Glycine max]	GO:0000776 GO:0019237 GO:0051382	370.9	417.1	289.0	193.2	178.7	233.3
Ca_01268	0.0127	-0.0035	0.0132	unknown protein		221.1	191.7	172.0	133.2	126.6	140.3
Ca_01279	0.0076	-0.0113	0.0136	Unknown protein		1.5	1.7	25.9	0.0	0.0	4.0
Ca_01484	0.0077	-0.0154	0.0172	Unknown protein		13.0	16.3	26.9	8.8	9.5	6.9
Ca_02668	0.0116	-0.0080	0.0141	unknown protein		289.5	448.5	294.7	269.1	195.2	127.5
Ca_03486	0.0119	-0.0075	0.0140	Unknown protein		79.8	50.4	72.6	29.1	36.3	41.7
Ca_03990	0.0134	-0.0037	0.0139	unknown protein		548.8	289.3	344.2	238.5	241.5	205.7
Ca_04030	0.0156	-0.0015	0.0157	unknown protein		847.0	594.4	559.5	426.5	425.4	426.0
Ca_04378	0.0090	-0.0120	0.0150	Unknown protein		2.0	1.3	5.9	0.0	3.0	0.0
Ca_06121	0.0103	-0.0105	0.0147	Unknown protein		1.8	6.3	0.0	0.0	0.0	0.0
Ca_06414	0.0128	-0.0158	0.0204	Unknown protein		0.0	0.5	0.9	0.0	0.0	0.0
Ca_08236	0.0109	-0.0094	0.0144	Unknown protein		4.4	9.3	0.0	0.0	0.0	0.0
Ca_09365	0.0105	-0.0126	0.0164	Unknown protein		24.6	64.0	3.6	0.0	1.2	4.1
Ca_09430	0.0025	-0.0134	0.0137	Unknown protein		9.4	29.4	20.7	13.9	10.9	24.3
Ca_09769	0.0101	-0.0083	0.0131	Unknown protein		1.5	20.6	4.9	0.0	0.0	0.0
Ca_10564	0.0090	-0.0126	0.0155	unknown protein		520.9	229.8	928.8	72.3	128.1	387.4
Ca_11177	0.0132	-0.0008	0.0132	Unknown protein		171.4	142.2	88.7	47.2	77.1	35.9
Ca_12037	0.0109	-0.0117	0.0159	Unknown protein		1.6	10.8	3.5	0.0	3.3	0.0
Ca_12610	0.0102	-0.0082	0.0131	Unknown protein		38.5	45.4	28.5	14.9	21.1	15.4
Ca_12833	0.0119	-0.0118	0.0168	Unknown protein		0.0	19.4	11.0	1.0	0.0	0.0
Ca_14992	0.0124	-0.0073	0.0143	unknown protein	GO:0005515	399.0	304.5	296.6	225.0	248.8	190.4
Ca_15579	0.0066	-0.0141	0.0155	unknown protein		61.0	37.6	57.1	22.8	39.0	34.6
Ca_16625	0.0045	-0.0135	0.0142	Unknown protein		4.8	7.3	14.8	5.7	3.5	9.4
Ca_17232	0.0093	-0.0121	0.0153	Unknown protein		0.0	0.0	4.3	0.0	0.0	0.0
Ca_17819	0.0073	-0.0108	0.0131	Unknown protein		0.0	0.0	4.2	0.0	0.0	0.0
Ca_17834	0.0064	-0.0113	0.0130	Unknown protein		0.0	4.1	4.6	0.0	2.7	0.0
Ca_18101	0.0109	-0.0098	0.0146	Unknown protein		54.0	79.3	35.7	15.3	15.3	16.9
Ca_18213	0.0081	-0.0121	0.0146	Unknown protein		4.6	11.8	20.3	5.4	8.9	6.1
Ca_19773	0.0129	-0.0046	0.0137	Unknown protein		166.8	426.5	58.9	17.0	49.7	16.6
Ca_19838	0.0126	-0.0140	0.0188	Unknown protein		0.0	1.3	1.3	0.0	0.0	0.0
Ca_19926	0.0074	-0.0118	0.0140	Unknown protein		6.7	4.3	9.0	0.0	5.5	6.9
Ca_19953	0.0105	-0.0088	0.0137	Unknown protein		1.6	8.1	0.0	0.0	0.0	0.0
Ca_20179	0.0075	-0.0109	0.0132	Unknown protein		0.0	0.0	19.9	0.0	0.0	0.0
Ca_20199	0.0080	-0.0125	0.0148	Unknown protein		55.5	45.3	51.9	42.6	14.8	23.8
Ca_20531	0.0132	-0.0130	0.0186	Unknown protein		12.4	13.5	15.0	4.3	0.0	6.7
Ca_22418	0.0110	-0.0099	0.0148	Unknown protein		3.6	5.5	0.0	0.0	0.0	0.0
Ca_23635	0.0104	-0.0106	0.0149	Unknown protein		1.5	0.0	7.3	0.0	0.0	0.0
Ca_23955	0.0122	-0.0076	0.0144	Unknown protein		50.3	47.7	61.4	61.2	6.4	0.0
Ca_24214	0.0107	-0.0076	0.0131	Unknown protein		6.9	13.1	0.6	0.0	0.0	0.0
Ca_25196	0.0089	-0.0113	0.0144	Unknown protein		6.1	22.6	10.7	6.2	5.3	7.5
Ca_25277	0.0045	-0.0123	0.0131	Unknown protein		45.9	29.5	73.2	28.8	49.0	26.9
Ca_25637	0.0138	-0.0018	0.0139	Unknown protein		79.0	69.1	25.7	30.0	6.1	8.3
Ca_25649	0.0092	-0.0108	0.0142	Unknown protein		10.5	4.0	10.8	5.3	3.6	0.0
Ca_25773	0.0089	-0.0110	0.0141	Unknown protein		0.0	0.0	7.3	0.0	0.0	0.0
Ca_25812	0.0083	-0.0118	0.0144	Unknown protein		0.0	0.0	5.7	0.0	0.0	0.0
Ca_26260	0.0036	-0.0139	0.0144	Unknown protein		4.7	14.2	14.8	4.8	10.6	11.2
Ca_26557	0.0052	-0.0122	0.0133	Unknown protein		70.2	81.0	90.7	56.6	46.9	51.6
Ca_26601	0.0086	-0.0108	0.0138	Unknown protein		0.0	0.0	0.3	0.0	0.0	0.0
Ca_27242	0.0088	-0.0099	0.0132	Unknown protein		3.2	0.0	5.5	2.4	0.0	0.0
Ca_27358	0.0100	-0.0162	0.0190	Unknown protein		7.8	21.2	13.7	4.3	2.1	13.1
Ca_29300	0.0057	-0.0131	0.0143	unknown protein		16.5	28.9	27.0	20.3	16.7	20.7
Ca_16485	0.0116	-0.0061	0.0130	urease accessory protein G	GO:0003924 GO:0005524 GO:0005737 GO:0006184 GO:0016151 GO:0016530 GO:0042803	167.8	68.4	88.1	66.2	42.9	67.3
Ca_19417	0.0140	-0.0092	0.0168	Vesicle transport protein SEC22 n=1 Tax=Rhodosporidium toruloides (strain NP11) RepID=M7XPL5_RHOT1	GO:0006810 GO:0016021 GO:0016192	615.3	509.7	533.2	394.8	451.1	436.8
Ca_04351	0.0131	-0.0027	0.0134	Vesicle transport v-SNARE family protein	GO:0005515 GO:0006886 GO:0016020 GO:0016192	240.4	313.6	153.8	128.9	112.3	169.4
Ca_06376	0.0132	-0.0014	0.0133	vesicle-associated membrane protein 725	GO:0006810 GO:0016021 GO:0016192	553.4	434.3	462.7	356.7	413.6	332.5
Ca_16724	0.0024	-0.0138	0.0140	VQ motif-containing protein		153.9	272.1	227.5	190.5	178.4	189.0
Ca_18833	0.0103	-0.0116	0.0155	V-type proton ATPase 16 kDa proteolipid subunit-like [Glycine max]	GO:0015078 GO:0015991 GO:0033177 GO:0033179	5282.0	5892.7	5396.1	4165.8	4356.4	4729.4
Ca_02002	0.0144	-0.0030	0.0147	WD repeat-containing protein 3-like isoform X1 [Glycine max]	GO:0005515	216.6	195.2	184.8	158.4	163.5	129.4
Ca_14206	0.0048	-0.0127	0.0136	WD repeat-containing protein 44-like [Glycine max]	GO:0005515	72.8	153.1	106.3	95.4	76.8	87.5
Ca_03193	0.0131	0.0022	0.0132	WD repeat-containing protein 5-like [Glycine max]	GO:0005515	1188.8	1054.0	817.5	807.4	753.3	700.0
Ca_15499	0.0078	-0.0111	0.0136	WD repeat-containing protein 61-like [Glycine max]	GO:0005515	0.0	0.0	10.6	0.0	0.0	1.2
Ca_01061	0.0139	-0.0058	0.0150	WD-40 repeat family protein	GO:0005515	971.8	1129.1	1078.4	792.3	856.7	797.2
Ca_00646	0.0047	-0.0140	0.0148	wound-responsive family protein		202.0	204.7	224.9	140.7	180.6	98.1
Ca_00661	0.0148	-0.0077	0.0167	wound-responsive family protein		261.8	218.5	188.1	77.0	138.0	75.9

Ca_24469	0.0079	-0.0110	0.0135	YABBY-like transcription factor CRABS CLAW-like protein		5.5	26.8	15.3	7.8	10.0	9.2
Ca_29046	0.0098	-0.0101	0.0141	YbjP n=35 Tax=Enterobacter RepID=G8LGU5_ENTCL		0.0	1.3	3.6	0.0	0.0	0.0
Ca_16449	0.0140	-0.0041	0.0146	Y-family DNA polymerase H	GO:0003684 GO:0003887 GO:0006281	469.6	499.6	380.9	403.4	122.0	151.7
Ca_23600	0.0143	-0.0092	0.0169	Zinc finger (C3HC4-type RING finger) family protein	GO:0005515 GO:0008270	839.4	802.1	820.3	718.1	695.6	518.1
Ca_08419	0.0048	-0.0127	0.0135	zinc finger HIT domain-containing protein 2-like isoform X3 [Glycine max]		4.8	14.7	15.7	10.8	5.1	5.8
Ca_06714	0.0057	-0.0135	0.0146	zinc finger protein CONSTANS-LIKE 15 [Glycine max]	GO:0005515 GO:0005622 GO:0008270	658.3	678.5	681.4	571.6	539.4	632.6
Ca_21388	0.0120	-0.0147	0.0190	zinc-finger protein 2	GO:0046872	99.0	156.3	104.8	52.8	60.2	73.5

Supplemental Table SM5 List of chickpea genes and annotations in known pathways for early signalling and responses to rhizobia. Annotations were assigned based on either protein BLAST to known Medicago trunclatula or Arabidopsis homologues if indicated, or gene assignments based on the Cicer arietinum (ICC4958) genome. * taken from Plett et al., 2016 **genes identified based on Gifford et al., 2018

Chickpea Gene ID	Annotation	Common Abbreviation	Homologue
	**Common Symbiotic Signalling Pathway**		
Ca_02780	Doesn't make infections1	DMI1	*
Ca_10338	Nuclear pore complex protein85	NUP85	*
Ca_03175	Nuclear pore complex protein133	NUP133	*
Ca_22916	Nucleoporin	NENA	*
Ca_02929	Nodule signalling pathway 1	NSP1	*
Ca_13244	Nodule signalling pathway 2	NSP2	*
Ca_24300	Doesn't make infections1	DMI2	*
Ca_20398	Doesn't make infections1	DMI3	*
Ca_13802	Cyclops	CYCLOPS	Medtr5g026850
Ca_13508	Nod factor perception1	NFR1	Medtr5g086130
Ca_22860	Nod factor perception5	NFR5	Medtr5g019040
Ca_05073	Ethylene responsive transcription factor	ERN1	Medtr7g085810
Ca_03225	Nodule Inception	NIN1	Medtr5g099060
	**Nitrogen transport**		
Ca_18399	High affinity nitrate transporter	NRT2.1	
Ca_18230	High affinity nitrate transporter	NRT1.2	
Ca_19740	High affinity nitrate transporter	NRT1.2	
Ca_21340	High affinity nitrate transporter	NRT1.2	
Ca_02467	High affinity nitrate transporter	NRT1.2	
Ca_03334	High affinity nitrate transporter	NRT1.2	
Ca_07682	High affinity ammonium importer	AMT1	
Ca_07428	High affinity ammonium importer	AMT1	
Ca_08062	High affinity ammonium importer	AMT1	
Ca_08902	High affinity ammonium importer	AMT1	
Ca_12383	High affinity ammonium importer	AMT1	
Ca_15601	Ammonium importer	AMT2	
Ca_05448	Ammonium importer	AMT2	
Ca_08260	Ammonium importer	AMT2	
Ca_20569	Ammonium importer	AMT3	
	**Autoregulation of nodulation pathway**		
Ca_17128	CLE12/13	CLE12/13	Medtr4g079630/Medtr4g079610
Ca_06814	CLE12/13	CLE12/13	Medtr4g079630/Medtr4g079610
Ca_03248	Root determined nodulation1	RDN1	Medtr5g089520
Ca_19863	Root determined nodulation1	RDN1	Medtr5g089520
Ca_18781	CLAVATA1/NARK	CLAVATA1/NARK	At1g75820
Ca_18331	CLAVATA1/NARK	CLAVATA1/NARK	At1g75820
Ca_22714	CLAVATA1/NARK	CLAVATA1/NARK	At1g75820
Ca_02657	CLAVATA1/NARK	CLAVATA1/NARK	At1g75820
Ca_03053	CLAVATA1/NARK	CLAVATA1/NARK	At1g75820
Ca_24585	Too Much Love	TML	Medtr7g029290/At3g27150
	**Cytokinin responses**		
Ca_15433	Cytokinin response1	CRE1	Medtr8g106150
Ca_15232	Cytokinin response1	CRE1	Medtr8g106150

Ca_03183	Cytokinin response1	CRE1	Medtr8g106150
Ca_16374	Cytokinin response1	CRE1	Medtr8g106150
Ca_21371	Response regulator	RR4/ARR5	Medtr5g036480
Ca_21195	Response regulator	RR4/ARR5	Medtr5g036480
Ca_13482	Response regulator	RR4/ARR5	Medtr5g036480

Auxin responses

Ca_21553	Auxin efflux carrier	PIN1/2	AT1G73590.1
Ca_29550	Auxin efflux carrier	PIN1/3	AT5G57090.1
Ca_06816	Auxin efflux carrier	PIN1/4	
Ca_10813	Auxin efflux carrier	PIN1/5	
Ca_13628	Auxin efflux carrier	PIN1/6	
Ca_15407	Auxin efflux carrier	PIN1/7	
Ca_16980	Auxin efflux carrier	PIN1/8	
Ca_04205	GH3- auxin dependent promoter		
Ca_22765	GH3- auxin dependent promoter		
Ca_20291	GH3- auxin dependent promoter		
Ca_25795	GH3- auxin dependent promoter		
Ca_09884	GH3- auxin dependent promoter		
Ca_20578	GH3- auxin dependent promoter		
Ca_20579	GH3- auxin dependent promoter		
Ca_20580	GH3- auxin dependent promoter		
Ca_26224	GH3- auxin dependent promoter		
Ca_00633	GH3- auxin dependent promoter		
Ca_03961	GH3- auxin dependent promoter		
Ca_07546	GH3- auxin dependent promoter		
Ca_07505	GH3- auxin dependent promoter		
Ca_07255	GH3- auxin dependent promoter		
Ca_17970	GH3- auxin dependent promoter		

Flavonoid Pathways

Flavenoid biosynthesis

Ca_13666	chalcone synthase [Glycine max]
Ca_17280	chalcone synthase-like [Glycine max]
Ca_22255	chalcone synthase 7 [Glycine max]
Ca_22256	chalcone synthase 7 [Glycine max]
Ca_22257	chalcone synthase 7 [Glycine max]
Ca_24067	chalcone synthase 7 [Glycine max]
Ca_25111	chalcone synthase-like [Glycine max]
Ca_25926	chalcone synthase-like [Glycine max]
Ca_30233	chalcone synthase 7 [Glycine max]
Ca_01020	Chalcone-flavanone isomerase family protein
Ca_01035	Chalcone-flavanone isomerase family protein
Ca_08417	Chalcone-flavanone isomerase family protein
Ca_11186	chalcone-flavanone isomerase family protein
Ca_11187	chalcone-flavanone isomerase family protein
Ca_14086	Chalcone-flavanone isomerase family protein
Ca_01100	flavonol synthase [Glycine max]
Ca_03593	flavonol synthase [Glycine max]
Ca_03595	flavonol synthase [Glycine max]
Ca_08253	Flavonol_synthase/flavanone_3-hydroxylase
Ca_13198	Flavonol_synthase/flavanone_3-hydroxylase
Ca_13212	Flavonol_synthase/flavanone_3-hydroxylase
Ca_14179	Flavonol_synthase/flavanone_3-hydroxylase
Ca_19467	Flavonol_synthase/flavanone_3-hydroxylase
Ca_22636	Flavonol_synthase/flavanone_3-hydroxylase

Ca_29502	Flavonol_synthase/flavanone_3-hydroxylase
Ca_02924	Flavonoid_3'-monooxygenase
Ca_15918	Flavonoid_3'-monooxygenase
Ca_21382	Flavonoid_3'-monooxygenase
Ca_25261	Flavonoid_3'-monooxygenase
Ca_12872	Flavonoid_3',5'-hydroxylase
**Flavone Biosynthesis**	
Ca_24494	Flavonoid_4'-O-methyltransferase
Ca_20596	Flavone_synthase
**Flavonol biosynthesis**	
Ca_21490	Flavonoid_3-O-glucosyltransferase
Ca_21491	Flavonoid_3-O-glucosyltransferase
**Isoflavonoid biosynthesis**	
Ca_10540	2-hydroxyisoflavanone_dehydratase
Ca_10541	2-hydroxyisoflavanone_dehydratase
Ca_12526	2-hydroxyisoflavanone_dehydratase
Ca_16785	2-hydroxyisoflavanone_dehydratase
Ca_22194	2-hydroxyisoflavanone_dehydratase
Ca_22195	2-hydroxyisoflavanone_dehydratase
Ca_27834	2-hydroxyisoflavanone_dehydratase
Ca_20595	Naringenin,2-oxoglutarate_3-dioxygenase
Ca_23335	Naringenin,2-oxoglutarate_3-dioxygenase
Ca_12738	Isoflavone_2'-hydroxylase
Ca_12739	Isoflavone_2'-hydroxylase
Ca_16420	Isoflavone_2'-hydroxylase
Ca_16421	Isoflavone_2'-hydroxylase
Ca_16422	Isoflavone_2'-hydroxylase
Ca_22769	Isoflavone_2'-hydroxylase
Ca_24656	Isoflavone_2'-hydroxylase
Ca_24971	Isoflavone_2'-hydroxylase
Ca_29720	Isoflavone_2'-hydroxylase
Ca_01757	Isoflavone_2'-hydroxylase
Ca_12751	Isoflavone_7-O-glucosyltransferase_1
Ca_19328	Isoflavone_7-O-glucosyltransferase_2
Ca_22348	Isoflavone_7-O-glucosyltransferase_3
Ca_22349	Isoflavone_7-O-glucosyltransferase_4
Ca_22394	Isoflavone_7-O-glucosyltransferase_5
Ca_23183	Isoflavone_7-O-glucosyltransferase_6
Ca_23184	Isoflavone_7-O-glucosyltransferase_7
Ca_23216	Isoflavone_7-O-glucosyltransferase_8
Ca_24348	Isoflavone_7-O-glucosyltransferase_9
Ca_24349	Isoflavone_7-O-glucosyltransferase_10
Ca_24350	Isoflavone_7-O-glucosyltransferase_11
Ca_25266	Isoflavone_7-O-glucosyltransferase_12
Ca_11922	Isoflavone_7-O-glucosyltransferase_13
Ca_16352	Isoflavone_4'-O-methyltransferase
Ca_16353	Isoflavone_4'-O-methyltransferase
Ca_16354	Isoflavone_4'-O-methyltransferase

Ca_16357	Isoflavone-7-O-methyltransferase_9
